# Impact of genetic variants linked to liver fat and liver volume on MRI-mapped body composition

**DOI:** 10.1016/j.jhepr.2025.101468

**Published:** 2025-06-02

**Authors:** Shafqat Ahmad, Germán D. Carrasquilla, Taro Langner, Uwe Menzel, Nouman Ahmad, Sergi Sayols-Baixeras, Koen F. Dekkers, Beatrice Kennedy, Filip Malmberg, Ulf Hammar, María J. Romero-Lado, Jenny C. Censin, Diem Nguyen, Andrés Martínez Mora, Tuomas O. Kilpeläinen, Lars Lind, Jan W. Eriksson, Robin Strand, Joel Kullberg, Håkan Ahlström, Tove Fall

**Affiliations:** 1 Molecular Epidemiology, Department of Medical Sciences, Uppsala University, Uppsala, Sweden; 2 Preventive Medicine Division, Harvard Medical School, Brigham and Women's Hospital, Boston, MA, United States; 3 School of Natural Sciences, Technology and Environmental Studies, Södertörn University, Huddinge, Sweden; 4 Novo Nordisk Foundation Center for Basic Metabolic Research, Faculty of Health and Medical Sciences, University of Copenhagen, Copenhagen, Denmark; 5 Novo Nordisk Foundation Center for Genomic Mechanisms of Disease, Broad Institute of MIT and Harvard, Cambridge, MA, United States; 6 Radiology, Department of Surgical Sciences, Uppsala University, Uppsala, Sweden; 7 Antaros Medical AB, Mölndal, Sweden; 8 Medical Epidemiology, Department of Surgical Sciences, Uppsala University, Uppsala, Sweden; 9 CIBER Cardiovascular Diseases (CIBERCV), Instituto de Salud Carlos III, Madrid, Spain; 10 Department of Information Technology, Uppsala University, Uppsala, Sweden; 11 Big Data Institute at the Li Ka Shing Centre for Health Information and Discovery, University of Oxford, Oxford, UK; 12 Wellcome Centre for Human Genetics, University of Oxford, Oxford, UK; 13 Clinical Epidemiology, Department of Medical Sciences, Uppsala University, Uppsala, Sweden; 14 Clinical Diabetology and Metabolism, Department of Medical Sciences, Uppsala University, Uppsala, Sweden

**Keywords:** Genetic variation, Metabolic dysfunction-associated steatotic liver disease, Chronic liver disease, Metabolic disease

## Abstract

**Background & Aims:**

A quarter of the world population is estimated to have metabolic dysfunction-associated steatotic liver disease. Here, we aim to understand the impact of liver trait-associated genetic variants on fat content and tissue volume across organs and body compartments and on a large set of biomarkers.

**Methods:**

Genome-wide association analyses were performed on liver fat and liver volume estimated with magnetic resonance imaging in up to 27,243 unrelated European participants from the UK Biobank. Identified variants were assessed for associations with fat fraction and tissue volume in >2 million ‘Imiomics’ image elements in 22,261 individuals and with circulating biomarkers in 310,224 individuals.

**Results:**

We confirmed four liver fat and nine liver volume previously reported genetic variants (*p* values <5 × 10^-8^). We further found evidence suggestive of a novel liver volume locus, *ADH4*, where each additional T allele increased liver volume by 0.05 SD (SE = 0.01, *p* value = 3.3 × 10^-8^). The Imiomics analyses showed that liver fat-increasing variants were specifically associated with fat fraction of the liver tissue (*p* values <2.8 × 10^-3^) and with higher inflammation, liver and renal injury biomarkers, and lower lipid levels. Associations of liver volume variants with fat content, tissue volume, and biomarkers were more heterogeneous, for example the liver volume-increasing alleles at *CENPW* and *PPP1R3B* were associated with higher skeletal muscle volumes and were more pronounced in men, whereas the *GCKR* variant was negatively associated with lower skeletal muscle volumes in women (*p* values <2.8 × 10^-3^).

**Conclusions:**

Liver fat-increasing variants were mostly linked to fat fraction of the liver and were positively associated with some adverse metabolic biomarkers and negatively with lipids. In contrast, liver volume-associated variants showed a less consistent pattern across organs and biomarkers.

**Impact and implications:**

Liver fat and liver volume are common metabolic traits with a strong genetic component, yet the extent to which they exert organ-specific *vs.* systemic effects remains poorly defined. By integrating genome-wide association analyses and high-resolution neck-to-knee magnetic resonance imaging data through the Imiomics framework, this study reveals distinct genetic architectures for liver fat and liver volume, including sex-specific effects. These findings provide new insights into the biological, organ-level, tissue-specific, and systemic characteristics of steatotic liver disease and its genetic determinants. The results may inform the development of precision imaging genetic approaches, biomarker discovery, and stratified risk assessment strategies, while reinforcing the importance of incorporating sex-specific analyses in future research and clinical applications.

## Highlights

• MRI-based GWAS identified four liver fat and 10 liver volume-associated variants.
• Liver fat variants had localized effects on the liver, with limited effects in other organs, as shown by Imiomics voxel-wise mapping.
• Imiomics analysis revealed heterogeneous and sex-specific effects of liver volume variants across organs and tissues.
• We found evidence suggestive of a novel liver volume locus, *ADH4.*
• Liver fat variants were linked to increased markers of inflammation, liver injury, and lower lipid levels.

## Graphical abstract

**Figure:**
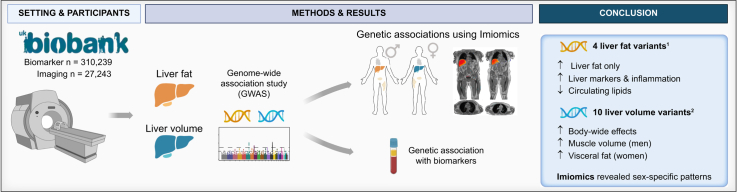

## Introduction

Metabolic dysfunction-associated steatotic liver disease (MASLD) is the most common chronic liver disease, with its global prevalence estimated to have increased from 25% to 38% over the past three decades – a trend that is expected to continue.^1^ MASLD is characterized by abnormal liver fat accumulation and can lead to severe liver conditions such as cirrhosis, liver failure, and hepatocellular carcinoma.^2^^,^^3^ It is also an independent risk factor for extrahepatic complications such as cardiovascular disease, type 2 diabetes, and chronic kidney disease.^4^ Magnetic resonance imaging (MRI) is considered the non-invasive gold standard method for liver fat quantification given its accuracy and reproducibility, surpassing traditional biopsy methods in safety and reliability.^5^^,^^6^ MRI provides precise and reproducible quantitative volumetric assessments of hepatic steatosis^5^ and can be used to measure liver volume. Increased liver volume has been observed in the early stages of MASLD and is often reduced in cirrhosis.^7^^,^^8^

Understanding the genetic architecture of MASLD phenotypes can enhance insights into pathophysiology and aid in discovering new molecular targets. Twin and family studies have estimated the heritability of MASLD to be 22–70%.^9^ Although genetic studies on MASLD and hepatic function biomarkers are expanding,[10], [11], [12] most research is based on the UK Biobank, and the largest analysis for MRI-derived liver phenotypes includes approximately 37,000 participants.^3^^,^[13], [14], [15], [16] Studies of the impact of liver fat- and liver volume-independent variants across hepatic and extrahepatic neck-to-knee regions are still lacking.

Epidemiological evidence shows clear sex differences in ectopic fat distribution,^17^ but most genetic studies combine the analysis for both sexes, overlooking such differences. To address these gaps, our study employed neck-to-knee 3D voxel-based maps (‘Imiomics’) to provide spatially detailed information about organ morphology, fat content, and tissue volume across different sex-specific body maps based on MRI data using the UK Biobank.^18^ Thus, we aimed to investigate the associations between liver fat- and liver volume-associated genetic variants and detailed measurements of fat content and tissue volume across various body compartments and organs. Additionally, we explored how these genetic variants were associated with clinical and cardiometabolic biomarkers.

## Materials and methods

### Ethics statement

This research was conducted using the UK Biobank resource under application number 14237 and written informed consent from all participants was provided before the study. The UK Biobank has ethical approval from the Northwest Multi-Centre Research Ethics Committee (ref: 11/NW/0382) and the current study was further approved by the Swedish Ethical Review Authority (DNR 2019-03073).

### Study sample and measurements

The UK Biobank is a prospective epidemiological cohort with deep phenotypic and genetic data collected for 502,655 individuals aged 40–69 years at recruitment 2006–2010 across 22 research centers in the UK.^19^^,^^20^ At the baseline assessment, participants answered questions about lifestyle and sociodemographic parameters, provided biological samples, and underwent clinical measurements. Details on the baseline assessment and biochemical assays have been published previously.[19], [20], [21] A summary of the phenotypes relevant to this study, including anthropometric and biomarkers measures, alcohol intake, and clinical diagnosis of MASLD and chronic liver disease (CLD), is found in the Supplementary Materials and methods and Table S1.

### Assessment of liver fat

Starting in 2014, a randomly selected subset of UK Biobank participants, up to 100,000 participants, are continuously invited to a neck-to-knee MRI. For the current analysis, imaging data was extracted for 38,949 participants in late 2019.^22^ Neck-to-knee body MRI scans (Data-Field 20201) were acquired using a Siemens 1.5T MAGNETOM Aera (Siemens Healthcare, Erlangen, Bavaria, Germany) with a dual-echo Dixon technique. Liver fat content was estimated using the proton density fat fraction (PDFF), which calculates liver fat as the proportion of fat to total tissue signal (fat + water). At the time of analysis, direct PDFF measurements were available for 9,893 participants.^23^ To extend liver fat estimation to a larger group, a neural network trained by Langner *et al.*^24^ was applied to the MRI images. This deep learning model was trained using reference liver fat measurements and validated through a 10-fold cross-validation process. After validation, the model was applied to infer liver fat content in the remaining imaging data cohort. This resulted in a high-quality dataset of 38,949 participants with measured or inferred liver fat. This method achieved a strong regression fit in cross-validation (R^2^ = 0.94).

### Assessment of liver volume

We quantified liver volume using image segmentation of the water MRI images from the abdominal scans. The liver segmentation model was trained on a dataset of 97 manually segmented livers, and subsequently applied to segment the livers of all participants. The model used was a modified 2.5D U-Net^25^ with a ResNet encoder pre-trained on ImageNet.^26^ It was trained and applied slice-by-slice on axial slices and in addition, included the closest neighboring slices as input data. From the liver segmentation method, the liver volume was derived by multiplying the number of voxels segmented with the known volume of each voxel. The segmentation approach was evaluated against manual reference segmentations, demonstrating the following performance: Dice coefficient: 0.95 ± 0.02, R^2^ = 0.86 and mean absolute error 77 ml.^27^ To ensure data quality, procedures were implemented to address potential segmentation challenges, including anatomical variations such as liver cysts, high liver iron, and prior liver resection. This resulted in a high-quality liver volume dataset of 36,957 participants.

### Genotyping and genome-wide association study

Genotyping, quality control, and imputation procedures in the UK Biobank cohort have previously been described in detail^19^ and are summarized in the Supplementary Materials and methods. By only including individuals that are part of in Data-Field 22020 provided by UK Biobank, we removed samples with excess heterozygosity, sex mismatch, high missingness (>0.05 on autosomes), and individuals who were not part of a maximal set of unrelated individuals up to third degree.^19^ Furthermore, only participants with European ethnic background were included, based on Data-Field 22006. Lastly, by matching this data set with the liver phenotype data, we achieved a final analytical genome-wide association study (GWAS) dataset of 27,243 for liver fat and 24,752 for liver volume. GWAS was conducted using PLINK v2.0 (www.cog-genomics.org/plink/2.0/). We excluded single nucleotide polymorphisms (SNPs) with call rate <0.9, minor allele count ≤30, Hardy-Weinberg exact test *p* value <1 × 10^-6^, and an imputation quality range (IMPUTE2 information metric) <0.8. To meet the assumption of normality and finite variance in the trait distribution, phenotype variables (liver fat and liver volume) were transformed using the age- and sex-adjusted two-stage inverse-normal transformation; the procedure is detailed in the Supplementary Materials and methods. We used a linear regression model adjusting for age at baseline, sex, genotyping arrays, and the first 20 principal components to control for population stratification. Genome-wide significance was set at *p* value <5 × 10^-8^, with genomic inflation assessed using quantile-quantile plots and the genomic inflation factor (λ). Body frame may constitute an important confounder in the association of genetic variants and liver volume, and as such the liver volume GWAS analysis was further adjusted for baseline height measures. Regional association plots of liver fat- and liver volume-associated genetic variants were visualized using LocusZoom.^28^ Chromosomal position is based on the GRCh37/hg19 build.

### Genetic correlations, pruning and conditional analysis, and gene prioritization

We used linkage disequilibrium (LD) score regression to assess genetic correlations^29^ using GWAS data on hepatic diseases from FinnGen.^30^ To identify independent SNPs, we used two procedures: (a) conditional joint (COJO) analysis^31^ and (b) LD clumping. Results from both approaches were comparable, and the COJO results are presented herein. Results from the original GWAS are available online (see Data availability statement). We retrieved GWAS summary statistics from a recent study on liver fat and liver volume in the UK Biobank for comparison.^13^ To assess the association with visceral adipose tissue (VAT) in a larger sample (n = 38,965), we cross-referenced with a VAT GWAS^32^ from the UK Biobank. Fine mapping of the signals was performed using CARMA,^33^ a Bayesian method that integrates functional annotations and accounts for LD discrepancies to refine causal variant identification, prioritizing variants with high posterior inclusion probabilities and was followed by gene prioritization using a combined SNP-to-gene strategy (cS2G).^34^ We also applied an alternative variant-to-gene (V2G) approach based on Open Target Genetics, using the otargen R package (R Foundation for Statistical Computing, Vienna, Austria).^35^ Details have been provided in the Supplementary Materials and methods.

### MRI voxel-based technique (‘Imiomics’)

Imiomics is a framework to visualize the association of non-image-based phenotypes with over 2 million 3D image elements based on neck-to-knee MRI. In Imiomics analysis, image registration is a crucial component where a target image is deformed to align with a sex-specific reference image by applying a deformation field. Each point in the reference image corresponds to a specific image point in the target image, and these point-to-point correspondences form the basis of the Imiomics analysis.^18^^,^^36^ We evaluated Imiomics maps for unrelated study participants of European ancestry with available MRI data, using the same selection as for study participants for GWAS analysis. For each individual, a ‘volume image’ with voxel-wise (>2 million 3D image elements) information about the magnitude of the deformation in each region was obtained, together with a ‘fat fraction image’ consisting of the original fat fraction image of each individual deformed into the template subject.

We assessed the association of each liver trait-associated SNP with fat fraction and tissue volume in each of these image elements using linear regression adjusted for age, age squared, total body fat, height, and 20 principal components stratified by sex in a sample of 11,483 men and 12,401 women. We display the results in Imiomics neck-to-knee association maps for the liver trait-increasing allele. We corrected *p* values with the Bonferroni method for 18 pre-defined body areas. Details of the Imiomics analysis are included in the Supplementary Materials and methods.

### Association of liver genetic variants with biomarkers, alcohol intake, and liver disease

Linear regression analyses were conducted to evaluate the potential impact of liver-associated genetic variants on 30 cardiometabolic biomarkers that were ln-transformed and alcohol intake, and are reported in the Supplementary Materials and methods and Table 1. These phenotypes were collected at the baseline visit (Table S1). These analyses were conducted in the biomarker subset consisting of 310,224 unrelated individuals of European ancestry from the UK Biobank who were not part of the liver GWAS analysis. The models were adjusted for age, sex, genotyping array, and the first 20 principal components. For imputed genotypes, dosages were converted to the closest integer. Further, we evaluated the association between genetic variants with the diagnosis of MASLD and CLD where these diseases were diagnosed using International Classification of Diseases-coded health records (details in the Supplementary Materials and methods). For this, we applied logistic regression analysis adjusting for the same covariates as in the linear models.

**Table 1:** Characteristics of the individuals from the UK Biobank at baseline who were in the MRI subset and in the biomarker subset.

Description	MRI subset (n = 27,243, 51% women)			Biomarker subset (n = 310,224, 54% women)		
	N	Mean	SD	N	Mean	SD
Age at baseline, years	27,242	55.7	7.4	310,224	57.6	8.0
Body mass index, kg/m^2^	27,241	26.6	4.2	309,185	27.5	4.8
Waist-to-hip ratio, cm/cm	27,241	0.9	0.1	309,614	0.9	0.1
Standing height, cm	27,241	169.8	9.2	309,529	168.7	9.2
Alkaline phosphatase, U/L	25,895	79.8	23.5	295,858	83.9	26.7
Alanine aminotransferase, U/L	25,892	23.0	13.8	295,722	23.6	14.0
Aspartate aminotransferase, U/L	25,797	25.7	9.9	294,750	26.2	10.7
Gamma-glutamyltransferase, U/L	25,885	33.7	34.6	295,694	37.7	42.3
Direct bilirubin, μmol/L	22,458	1.8	0.8	251,276	1.8	0.9
Total bilirubin, μmol/L	25,775	9.4	4.6	294,617	9.1	4.4
Apolipoprotein A, g/L	23,522	1.5	0.3	269,380	1.5	0.3
Apolipoprotein B, g/L	25,775	1.0	0.2	294,406	1.0	0.2
Lipoprotein A, nmol/L	20,738	44.1	49.3	235,076	44.2	49.5
C-reactive protein, mg/L	25,839	2.0	3.5	295,203	2.6	4.4
Glucose, mmol/L	23,619	5.0	1.0	270,718	5.1	1.2
Glycated hemoglobin, mmol/mol	25,829	35.0	5.1	295,772	36.0	6.6
HDL cholesterol, mmol/L	23,638	1.5	0.4	270,884	1.5	0.4
LDL cholesterol, mmol/L	25,839	3.6	0.8	295,293	3.6	0.9
Triglycerides, mmol/L	25,870	1.7	1.0	295,605	1.8	1.0
Cholesterol, mmol/L	25,895	5.7	1.1	295,844	5.7	1.1
Urea, mmol/L	25,870	5.3	1.2	295,658	5.4	1.4
Calcium, mmol/L	23,647	2.4	0.1	270,893	2.4	0.1
Albumin, g/L	23,647	45.4	2.5	271,001	45.2	2.6
Creatinine, μmol/L	25,874	72.5	14.0	295,700	72.4	18.1
Phosphate, mmol/L	23,602	1.2	0.2	270,485	1.2	0.2
Total protein, g/L	23,614	72.2	3.9	270,708	72.4	4.0
Urate, μmol/L	25,865	304.0	77.4	295,460	310.0	80.5
Cystatin C, mg/L	25,893	0.9	0.1	295,825	0.9	0.2
Insulin-like growth factor-1, nmol/L	25,766	22.1	5.5	294,247	21.3	5.7
Sex hormone-binding globulin, nmol/L	23,418	52.0	27.8	268,397	51.8	27.6
Number of overweight (%)	16,630 (61)			209,361 (67.5)		
Number of obese (%)	4,844 (17.8)			77,350 (24.9)		

Values are reported as mean and SD. Overweight is defined as individuals having a body mass index (BMI) ≥25.0 kg/m^2^, and obese are individuals having a BMI ≥30.0 kg/m^2^. MRI, magnetic resonance imaging; N, sample size; NA, not applicable; SD, standard deviation.

## Results

Baseline characteristics and cardiometabolic biomarkers for the MRI subset (n = 27,243) and those in the biomarker subset (n = 310,224) are reported in Table 1, including measurement units. The mean (SD) age for individuals at baseline in the GWAS was 55.7 (7.4) years and the age at imaging was 64.6 (7.5) years. The mean liver fat content in the MRI subset was 3.9% (SD: 4.3), and the mean liver volume was 1,419 cm^3^ (SD: 300).

### GWAS analysis identified genetic variants of liver fat and liver volume

Four independent genetic variants were associated with liver fat (λ = 1.05) and 10 independent genetic variants were associated with liver volume (λ = 1.06) (Table 2, Fig. S1–S3). For liver fat, variants were located near the genes *PNPLA3*, *TM6SF2*, and *APOE*, all of which were previously reported as associated with liver fat in the UK Biobank (Table S2).^13^ Fine mapping using CARMA, which integrates functional annotations and accounts for LD structure to enhance causal variant identification, and cS2G suggested *MAU2, CILP2, APOE*, and *PNPLA3* as candidate genes, whereas the V2G approach suggested *MAU2, SUGP1, APOE*, and *SAMM50* (Table S2). The liver volume GWAS confirmed nine previously reported associated genetic variants, and suggested a novel associated intronic variant in *ADH4* (Tables 2 and S3 and Fig. S1 and S3).^13^ Gene prioritization was concordant between the two prioritization approaches for *GCKR* and *CENPW* (Table S2). We found one liver volume variant *LPAR* rs2304128 to be in moderate LD (R^2^ = 0.69) with the liver fat variant *TM6SF2* rs8107974. For both, *MAU2* was suggested as the causal gene by the V2G approach (Table S2).

**Table 2:** Independent genome-wide significant genetic variants for liver fat and liver volume and their prioritized genes. Although rs188247550 and rs7896518 are classified as intronic variants by OTG, they are not located within the genes closest to their transcription start sites (*TM6SF2* and *REEP3*, respectively) but within *SUGP1* and *JMJD1C*

SNP	Chr	Position∗	Nearest gene	EA	OA	EAF	Beta	SE	*p* value	Variant type	cS2G	OTG-V2G
**Liver fat variants**												
rs8107974	19	19388500	*TM6SF2*	T	A	0.08	0.29	0.02	7.3 × 10^-73^	Intronic	*MAU2*	*MAU2*
rs188247550	19	19396616	*TM6SF2*	T	C	0.01	0.27	0.04	6.2 × 10^-14^	Intronic	*CILP2*	*SUGP1*
rs429358	19	45411941	*APOE*	T	C	0.85	0.09	0.01	6.1 × 10^-15^	Missense	*APOE*	*APOE*
rs738408	22	44324730	*PNPLA3*	T	C	0.21	0.19	0.01	2.5 × 10^-78^	Synonymous	*PNPLA3*	*SAMM50*

**Liver volume variants**												
rs193084249	1	26987646	*ARID1A*	G	A	0.02	0.17	0.03	4.5 × 10^-09^	Intergenic	*ZDHHC18*	*ARID1A*
rs1260326	2	27730940	*GCKR*	T	C	0.39	0.08	0.01	4.3 × 10^-20^	Missense	*GCKR*	*GCKR*
rs79287178	3	172294500	*TNFSF10*	A	G	0.03	0.18	0.03	3.0 × 10^-11^	Intronic	NA	*TNFSF10*
rs6858148	4	100065917	*ADH4*	T	C	0.65	0.05	0.01	3.3 × 10^-08^	Intronic	*ADH4, ADH5*	*ADH5*
rs853966	6	127037236	*CENPW*	T	G	0.54	0.06	0.01	9.5 × 10^-11^	Regulatory region	*CENPW*	*CENPW*
rs4240624	8	9184231	*PPP1R3B*	G	A	0.09	0.18	0.02	3.0 × 10^-33^	Intronic	*RP11-115J16.1*	*PPP1R3B*
rs10881959	10	93507964	*TNKS2*	T	G	0.43	0.05	0.01	2.6 × 10^-09^	Intergenic	NA	*REEP3*
rs7896518	10	65104500	*REEP3*	A	G	0.57	0.07	0.01	2.3 × 10^-14^	Intronic	*TNKS2-DT*	*TNKS2*
rs139974673	15	44027885	*PDIA3*	C	T	0.02	0.25	0.03	3.2 × 10^-19^	Intronic	*CKMT1A*	*ADAL*
rs2304128	19	19746151	*LPAR2*	T	G	0.08	0.10	0.02	7.1 × 10^-10^	Intronic	*GMIP*	*MAU2*

The nearest gene is defined based on the distance to the start site. Gene prioritization results are from combined SNP-to-gene strategy (cS2G) and Open Targets Genetics (OTG) highest variant-to-gene (V2G) score. ∗Chromosomal position is based on the GRCh37/hg19 build. Linear regression models were used in these analyses, adjusting for age at baseline, sex, genotyping arrays, and the first 20 principal components to control for population stratification. The liver volume GWAS analysis was further adjusted for baseline height measures. Genome-wide significance was set at *p* value <5×10^-8^.

Chr, chromosome; EA, effect allele; EAF, effect allele frequency; NA, not applicable; OA, other allele; SE, standard error; SNP, single nucleotide polymorphism.

Additionally, genetic correlation analysis showed positive genetic correlations between our liver traits and MASLD, metabolic dysfunction-associated steatohepatitis (MASH), but negative correlations between liver fat and liver volume as illustrated in Fig. S4. Cross-referencing our variants with a previous GWAS^32^ on VAT and subcutaneous adipose tissue (SAT) in the abdominal region showed nominal associations of the *APOE* rs429358 liver fat-associated variant with increased VAT and SAT in the abdominal region. Moreover, the *TM6SF2* rs188247550 liver fat-associated variant was nominally associated with abdominal SAT and the *REEP3* rs7896518 liver volume-associated variant with VAT as detailed in Table S4.

### Association of liver variants with body composition using Imiomics maps

**Fig. 1:**
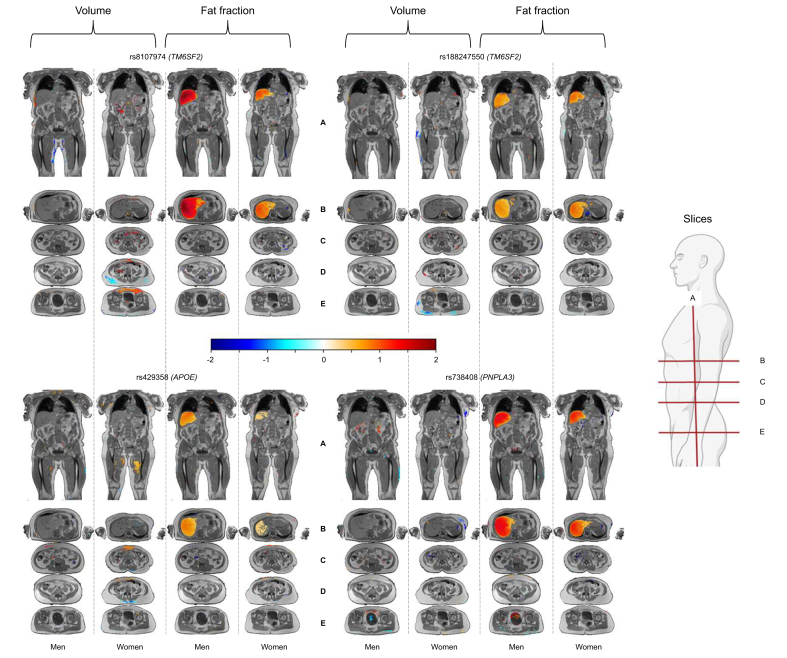
Neck-to-knee voxel maps of volume and fat fraction for four liver fat variants. The figure shows statistical 3D associations between magnetic resonance imaging (MRI) data and single nucleotide polymorphism (SNP) in men and women. Each panel displays volume maps for men, volume maps for women, fat fraction maps for men, and fat fraction maps for women. Linear regression models were used in these analyses, adjusting for age, age squared, total body fat, height, and 20 principal components stratified by sex. *p* values were corrected with the Bonferroni method for 18 pre-defined body areas. Significant associations (*p* value <2.8 × 10^-3^) are color-coded from negative (blue) to positive (red). For visualization purposes clipped beta values (from the lower 1% to the highest 1%) were for each experiment linearly rescaled between -2 and +2 maintaining the sign of the association. Non-significant regions are uncolored and instead show underlying anatomical MRI images for reference. Coronal slices (A) and axial slices at the liver (B), kidneys (C), abdomen (D), and hip (E) are shown. APOE, apolipoprotein E; PNPLA3, patatin like domain 3, 1-acylglycerol-3-phosphate O-acyltransferase; TM6SF2, transmembrane 6 superfamily member 2.

**Fig. 2:**
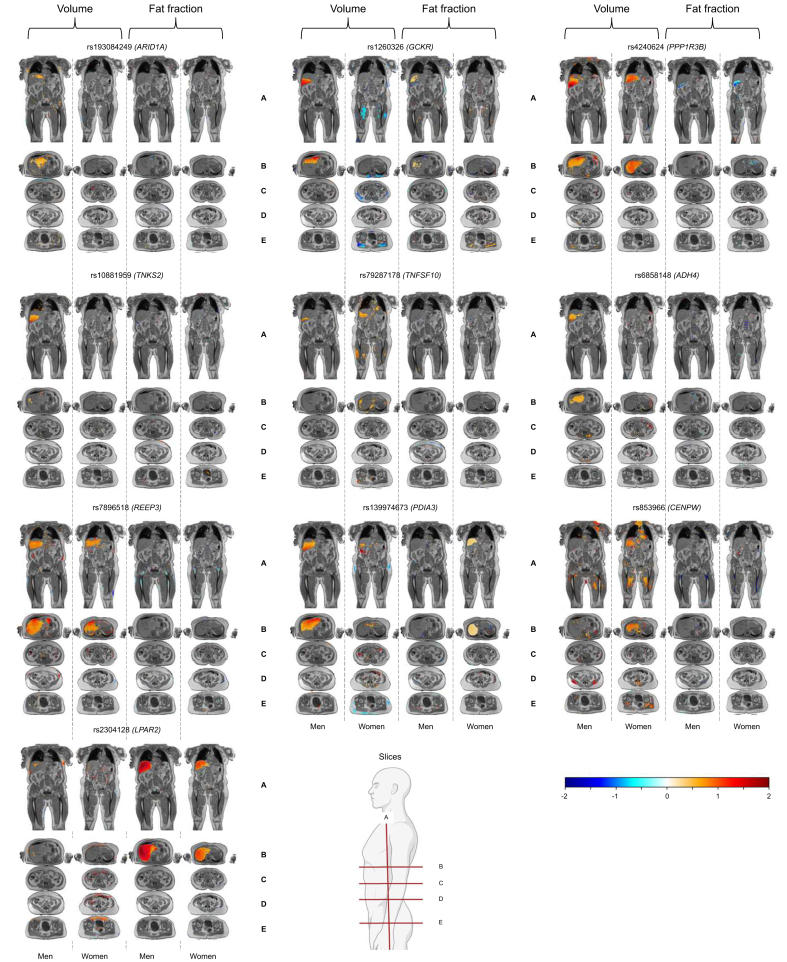
Neck-to-knee voxel maps of volume and fat fraction for 10 liver volume variants. The figure shows statistical 3D associations between magnetic resonance imaging (MRI) data and single nucleotide polymorphism (SNP) in men and women. Each panel displays volume maps for men, volume maps for women, fat fraction maps for men, and fat fraction maps for women. Linear regression models were used in these analyses, adjusting for age, age squared, total body fat, height, and 20 principal components stratified by sex. *p* values were corrected with the Bonferroni method for 18 pre-defined body areas. Significant associations (*p* value <2.8 × 10^-3^) are color-coded from negative (blue) to positive (red). For visualization purposes clipped beta values (from the lower 1% to the highest 1%) were for each experiment linearly rescaled between -2 and +2 maintaining the sign of the association. Non-significant regions are uncolored and instead show underlying anatomical MRI images for reference. Coronal slices (A) and axial slices at the liver (B), kidneys (C), abdomen (D), and hip (E) are shown. ADH4, alcohol dehydrogenase 4; ARID1A, AT-rich interaction domain 1A; CENPW, centromere protein W; GCKR, glucokinase regulatory protein; LPAR2, lysophosphatidic acid receptor 2; PDIA3, protein disulfide isomerase family A member 3; PPP1R3B, protein phosphatase 1 regulatory subunit 3B; REEP3, receptor accessory protein 3; TNFSF10, tumor necrosis factor ligand superfamily member 10; TNKS2, tankyrase 2.

Associations of liver fat and liver volume genetic variants are summarized for 18 pre-defined body areas in Fig. S5 and S6, respectively. We applied a significance threshold of *p* value <2.8 × 10^-3^, accounting for multiple comparisons. Figs. 1 and 2 illustrate the associations of the genetic variants with two-dimensional body maps at pre-defined sections. For each genetic variant, results are presented for the liver trait-increasing effect allele and reported using the closest gene name (Fig. S7–S20). Supplementary Movies S1–S4 and S5–S14 show associations for liver fat- and liver volume-associated genetic variants across the neck-to-knee region, respectively. Associations are shown in both axial and coronal planes.

The following is/are the supplementary data related to this article:

Video S1_rs8107974 5 Video S1

Video SS1_rs8107974 6 Video S2

Video SS2_rs188247550 7 Video S3

Video SS2_rs188247550 8 Video S4

Video SS3_rs429358 9 Video S5

Video SS3_rs429358 10 Video S6

Video SS4_rs738408 11 Video S7

Video SS4_rs738408 12 Video S8

Video SS5_rs193084249 13 Video S9

Video SS5_rs193084249 14 Video S10

Video SS6_rs1260326 15 Video S11

Video SS6_rs1260326 16 Video S12

Video SS7_rs79287178 17 Video S13

Video SS7_rs79287178 18 Video S14

Video SS8_rs6858148 19 Video S15

Video SS8_rs6858148 20 Video S16

Video SS9_rs853966 21 Video S17

Video SS9_rs853966 22 Video S18

Video SS10_rs4240624 23 Video S19

Video SS10_rs4240624 24 Video S20

Video SS11_rs10881959 25 Video S21

Video SS11_rs10881959 26 Video S22

Video SS12_rs7896518 27 Video S23

Video SS12_rs7896518 28 Video S24

Video SS13_rs139974673 29 Video S25

Video SS13_rs139974673 30 Video S26

Video SS14_rs2304128 31 Video S27

Video SS14_rs2304128 32 Video S28

#### Liver fat genetic variants

As expected, all four liver fat variants showed positive association with liver fat fraction in the Imiomics analysis in both men and women. We did not observe evidence for the effects of these genetic variants on fat fraction in any other compartments of the body (Figs 1, S5, and S7–S10, Supplementary Movies S1–S4). The liver fat variants showed a few associations with the volume of adipose tissue across parts of the studied body compartments (Figs 1 and S5). We observed signals for the *TM6SF2* rs188247550 and rs8107974 variants with lower body SAT, including lower volume of gluteal and thigh SAT in women (rs188247550), and lower volume of gluteal SAT in women and lower thigh SAT in men (rs8107974). The *TM6SF2* rs8107974 variant further showed regional signals for lower abdominal posterior SAT volume and higher thorax and abdominal SAT volume in women (Fig. S7). The *PNPLA3* variant was negatively regionally associated with thigh SAT in men and positively with higher gluteal SAT in women (Fig. S10). No associations were observed between the liver fat variants and the volume of the lung, heart, liver, and skeleton, except for a positive signal for parts of thigh muscle volume for the *APOE* variant (Fig. S5 and S9).

#### Liver volume variants

All liver volume variants showed positive associations with liver volume in the Imiomics except for the *LPAR2* variant (Fig. 2). However, the *ARID1A, GCKR, ADH4,* and *TNKS2* variants only showed an association with liver volume in men and *CENPW* only in women in this analysis (Figs 2 and S6). We observed a variety of body-wide effects of the genetic variants, where the liver volume-increasing allele of the *CENPW* variant had regional positive associations with volumes of gluteofemoral muscles in both women and men and thoracic and abdominal muscles in men (Fig. S15). For the *GCKR* variant, the association pattern contrasted with that of the *CENPW* variant, where regional negative associations were noted in women for the *GCKR* variant with skeletal muscle volumes and with lower thigh SAT in men (Fig. S12). The *ADH4* variant was associated to higher VAT in women, and greater abdominal skeletal muscle volume in men (Fig. S14). The *PPP1R3B* variant was associated with increased thoracic and gluteal skeletal muscle volume in men (Fig. S16). The *REEP3* and *PDIA3* variants was associated with greater heart volume in men (Fig. S18 and S19) and the *TNSF10* with larger heart volume in women (Fig. S13). The *PDIA3* variant also showed regional negative associations with gluteal SAT adipose tissue volumes in women (Fig. S19). The *LPAR2* variant was positively associated with thorax SAT in men and abdominal SAT in women (Fig. S20). There were only a few associations of liver volume variants with the fat fraction across tissue compartments (Figs 2 and S6). The *GCKR* variant was regionally positively associated with the fat fraction in the skeletal muscle of the gluteal area in women and with the fat fraction of the liver in men (Fig. S12). The *LPAR2* variant was positively associated with the liver fat fraction in both men and women (Fig. S20) and the *ADH4* variant was associated with the lower disc fat fraction in women (Fig. S14). Finally, the *CENPW* variant was negatively associated with the fat in femur in both men and women and with the fat fraction in spine and pelvic bone in men (Fig. S15).

### Genetic variants for liver fat content associated with various biomarkers and liver disease traits

**Fig. 3:**
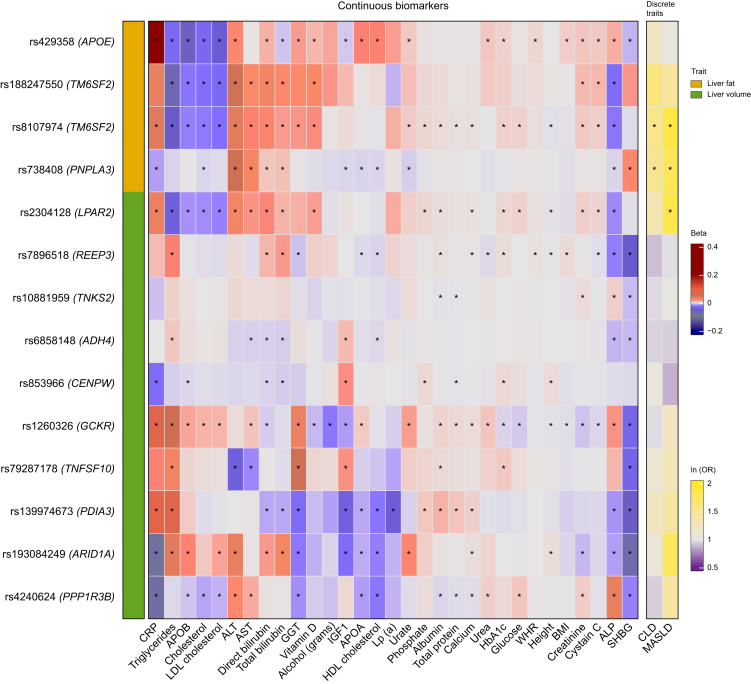
Associations of liver fat and liver volume variants with biomarkers and liver disease. Each row in the heatmap represents a liver fat or liver volume genetic variant. Liver fat variants are in orange, and liver volume variants are in green in the first column. Each column in the heatmap represents a biomarker or a discrete trait. The biomarkers are clustered with hierarchical clustering. The heatmap is color-coded by strength of association according to color legend. ∗*p* value <0.05. Linear regression models were used for analyses of continuous traits and logistic regression models for discrete traits. Models were adjusted for age, sex, genotyping array, and the first 20 principal components. All the biomarker concentrations were log-transformed before the analysis. Discrete traits are shown as natural log of the OR. ALP, alkaline phosphatase; ALT, alanine aminotransferase; AST, aspartate aminotransferase; APOA, apolipoprotein A; APOB, apolipoprotein B; CLD, chronic liver disease; CRP, C-reactive protein; GGT, gamma-glutamyl transferase; HBA1c, hemoglobin A1c; IGF1, insulin-like growth factor 1; Lp(a), lipoprotein A; MASLD, metabolic dysfunction-associated steatotic liver disease; OR, odds ratio; SHBG, sex hormone-binding globulin; WHR, waist-to-hip ratio.

Next, we tested the association of the identified liver fat variants with biomarkers, MASLD, CLD, and alcohol intake. All liver fat-increasing alleles were associated with increased alanine aminotransferase and direct bilirubin and were negatively associated with cholesterol. The *APOE* and *TM6SF2* variants were associated with increased markers of inflammation, glycemia and kidney markers, and increased risk of CLD, but negatively associated with LDL cholesterol, triglycerides, and apolipoprotein B. The *TM6SF2* rs8107974 and *PNPLA3* variants were associated with an increased risk of MASLD. The *PNPLA3* variant had a different pattern with negative associated with CRP, ALP, IGF1, apolipoprotein A, apolipoprotein B, HDL cholesterol, and urate. There were no associations with alcohol intake (Fig. 3).

### Genetic variants for liver volume-associated with various biomarkers and liver disease traits

We also analyzed the association of liver volume-increasing variants with biomarkers, MASLD, CLD, and alcohol intake. All variants were negatively associated with sex hormone binding globulin except *LPAR2* and *CENPW*. Variants in the *GCKR, PDIA3,* and *ARID1A* loci showed associations with most biomarkers (Fig. 3).

## Discussion

We leveraged MRI-based neck-to-knee 3D voxel-based mapping of liver fat and liver volume genetic markers to uncover metabolic and sex-specific patterns in the UK Biobank cohort. Our GWAS analysis confirmed 13 previously reported associations of genetic variants with liver traits^13^^,^^16^^,^^37^ and suggested a novel association of an intronic variant in *ADH4*, the alcohol dehydrogenase 4 gene, with liver volume. Our study extends previous liver phenotype GWAS analyses by mapping liver trait variants with neck-to-knee fat fraction and tissue volume at a granular spatial resolution using the Imiomics concept. Previous GWAS and candidate gene studies have characterized associations between liver trait-associated variants at *TM6SF2, PNPLA3*, and *GCKR* with MASLD and fat distribution,^16^^,^^38^ but these primarily focused on large-scale association analyses without spatially resolved assessments. We found liver fat genetic variants to be largely specifically associated with increased fat fraction in the liver. In contrast, liver volume genetic variants displayed more heterogeneous associations with tissue volume. Liver trait-associated variants were associated with a broad range of cardiometabolic biomarkers.

Our results for liver fat-associated variants in or near *TM6SF2*, *APOE*, and *PNPLA3* are in general consistent with previous reports showing that these variants are positively associated with markers of liver and kidney dysfunction and inflammation and negatively associated with cholesterol lipoproteins.[39], [40], [41], [42] Our Imiomics analysis showed that the four liver fat genetic variants were exclusively associated with increased fat fraction in the liver. The *PNPLA3* liver fat variant has been associated with lower body weight, BMI, and fat percentage in healthy individuals, particularly in women.^43^ Previous research demonstrated that genetically determined higher liver fat is causally associated with an increased risk of liver cirrhosis and hepatic cancer.^3^ In our study, we found positive genetic correlations between our liver traits and MASLD and MASH, indicating that these traits share features in their genetic background supporting previous findings.

Our novel finding of associations of variants in *ADH4* with liver volume suggests a potential role for alcohol metabolism genes in liver size and systemic fat distribution but warrants replication in external material. The *ADH4* variant was associated with higher VAT volume in women, greater abdominal skeletal muscle volume in men, and decreased fat fraction in discs in women. Liu *et al.*,^13^ who have previously assessed variants at the genome-wide scale with liver volume in the UK Biobank did not highlight this variant in a slightly larger and largely overlapping sample (*p* value = 1 × 10^-4^, n = 32,860). This could be because of differences in liver volume assessment, model specification, or sample differences. Our results showed a slightly stronger association in the height-adjusted model than in the non-adjusted model (3.3 × 10^-8^ *vs.* 1.0 × 10^-7^). In a previous large GWAS of cardiometabolic traits, the *ADH4* rs6858148 variant was associated with waist-to-hip ratio (*p* value = 5.2 × 10^-4^),^44^ whereas no association was found with MASLD,^10^ steatohepatitis,^30^ waist circumference,^45^ VAT, or abdominal SAT^32^ (Table S4).

Studies on liver volume genetics have reported that liver volume is associated with a higher risk of type 2 diabetes but not type 1 diabetes,^46^ and that liver volume is influenced by genetic factors related to abdominal fat distribution.^47^ We found the liver volume-associated variants near *ARID1A* and *GCKR* to be associated with liver function enzymes, lipid levels, and glucose metabolism, as previously reported.^48^^,^^49^ Moreover, variants at the *CENPW*, *PPP1R3B*, and *PDIA3* loci showed associations with glucose, CRP, and cholesterol markers, which is in agreement with previous findings.[50], [51], [52] Imiomics results for liver volume variants were heterogeneous. Previous studies on liver volume genetics are limited^13^ and, to the best of our knowledge, have not been studied in relation to fat content or tissue volume at a granular level. Previous research suggests that *PPP1R3B* may be involved in hepatic glycogen synthesis and may contribute to variations in liver phenotypes, including glycogen storage and bile acid composition, which in turn may affect liver volume.^53^^,^^54^ The liver volume variant near *LPAR2* was positively associated with liver fat fraction and likely represents the same locus as the liver fat variants in the *TM6SF2* locus, which were in moderate LD and showed similar patterns of associations. The *GCKR*, *CENPW,* and *PDIA3* genes have been previously reported to have pleiotropic effects,[55], [56], [57] and our findings on their non-specificity across body maps further support these effects. For *GCKR* and *CENPW* variants, we found opposing signals in men and women, where *GCKR* variant was positively associated with liver volume only in men and negatively with skeletal muscle in women, and *CENPW* variant positively associated with liver volume only in women and positively with skeletal muscle more prominently in men.

A strength of our study is its innovative use of neck-to-knee voxel-based MRI maps (Imiomics), to provide high spatial 3D resolution and a detailed understanding of liver fat and liver volume genetic variants across different body compartments and organs, while offering valuable insights into metabolic and sex-specific genetic effects. Although liver fat content was assessed through both MRI measures and an automated neural network trained on neck-to-knee MRI images, a strong correlation (R^2^ = 0.94) with the UK Biobank reference method confirms the reliability of our measures. However, limitations include the exclusive focus on participants of European ancestry, potentially limiting the generalizability of the findings. The volunteer-based nature of UK Biobank introduces a selection bias, which may limit the external validity of our findings and their generalizability to the broader population. Individuals who died before the imaging visit were not included in the study, which means that our study population might be biased toward a healthier sample. A limitation is that results could not be validated in other studies outside the UK Biobank. Further studies in other population groups with imaging are needed to confirm our findings, especially regarding the liver volume and Imiomics results. Despite the high-quality imaging data, there remains a possibility of bias attributable to MRI artifacts or segmentation errors. Additionally, as Imiomics is an emerging method, the extent of type I errors remains uncertain. Furthermore, we did not directly assess the predictive value of genetic and Imiomics-based phenotypes for clinical outcomes such as liver fibrosis, cirrhosis, cardiometabolic disease, or mortality. However, our findings provide a foundation for future studies to integrate longitudinal data and evaluate whether these genetic and imaging traits are associated with disease progression and patient outcomes – an essential step toward advancing precision medicine applications of Imiomics.

In summary, liver fat-associated genetic variants were largely specifically associated with hepatic fat and not extrahepatic. Further, liver fat variants were associated with markers of inflammation and liver injury and negatively linked to lipid biomarkers. Liver volume-associated genetic variants showed broader organ and tissue effects, including sex-specific impact on organ volume and fat, along with diverse biomarker associations. These results contribute to our understanding of how liver trait-associated variants can have sex-specific and heterogeneous effects on organ and body composition, with diverse cardiometabolic implications.

## Abbreviations

ADH4, alcohol dehydrogenase 4; ALP, alkaline phosphatase; ALT, alanine aminotransferase; APOA, apolipoprotein A; APOB, apolipoprotein B; ARID1A, AT-rich interaction domain 1A; AST, aspartate aminotransferase; CLD, chronic liver disease; COJO, conditional joint analysis; CREA, creatinine; CRP, C-reactive protein; cS2G, combined SNP-to-gene strategy; CYSC, cystatin C; DBIL, direct bilirubin; GCKR, glucokinase regulatory protein; GGT, gamma-glutamyl transferase; GWAS, genome-wide association study; HbA1c, hemoglobin A1c; IGF1, insulin-like growth factor 1; Imiomics, imaging-based phenomics; LD, linkage disequilibrium; MASH, metabolic-associated steatohepatitis; MASLD, metabolic dysfunction-associated steatotic liver disease; MRI, magnetic resonance imaging; OTG, Open Target Genetics; PDIA3, protein disulfide isomerase family A member 3; PPP1R3B, protein phosphatase 1 regulatory subunit 3B; REEP3, receptor accessory protein 3; SAT, subcutaneous adipose tissue; SHBG, sex hormone-binding globulin; SNP, single nucleotide polymorphism; TBIL, total bilirubin; TC, total cholesterol; TG, triglycerides; TNFSF10, tumor necrosis factor ligand superfamily member 10; UK Biobank, United Kingdom Biobank; VAT, visceral adipose tissue; V2G, variant-to-gene; WHR, waist-to-hip ratio.

## Authors' contributions

Obtained the funding, project administration, and supervision: JK, HA, TF. Conceptualized the study: SA, GDC, JCC, JK, HA, TF. Data curation: SA, GDC, TL, UM, NA, FM, RS, AMM, JK. Performed the formal analysis: SA, GDC, NA, UM, UH, MJRL, JCC, JK. Development or design of methodology in the creation of statistical models: SA, GDC, TL, UM, JCC, FM, RS, JK, TF. Software development and code implementation: UM, TL, FM, RS, JK. Preparation of visualization or data presentation: SA, GDC, UM, NA, JCC, SSB, MJRL, AMM, JK. Wrote the first draft: SA, GDC, UM, UH, DN, JK, TF. Provided interpretation of the results and critical feedback on the final version of the manuscript: all authors.

## Data availability statement

The code supporting this study is available at https://github.com/gerca/LiverGeneticsImiomic. Table S1 provides a detailed mapping of the UK Biobank variables used in this study, including their corresponding data fields. Researchers worldwide can access the phenotype and genotype data for the UK Biobank by applying for data access through the UK Biobank (https://www.ukbiobank.ac.uk/enable-your-research/register). Original GWAS results for liver fat and liver volume (height- and non-height adjusted) are available at the GWAS Catalog database under accession codes GCST90566817 (liver fat), GCST90566818 (liver volume, non-height adjusted), and GCST90566819 (liver volume, height adjusted).

## Conflicts of interest

JK and HA are co-founders, co-owners of, and part-time employees of Antaros Medical AB, BioVenture Hub, Mölndal, Sweden. JCC receives a salary from Sirona AB. Her contribution to this manuscript was performed prior to commencing employment. GDC was affiliated with the University of Copenhagen during the conduct of this research. He is currently employed at Novo Nordisk. This work was completed before his employment and was not funded or influenced by Novo Nordisk. Other co-authors reported no potential conflicts of interest relevant to this article.

Please refer to the accompanying ICMJE disclosure forms for further details.
