# Supplementary material for: Impact of genetic variants linked to liver fat and liver volume on MRI-mapped body composition
Source: JHEP Rep. 2025 Jun 2;7(9):101468. doi: 10.1016/j.jhepr.2025.101468 (PMC12355076; doi:10.1016/j.jhepr.2025.101468)
Supplement: Multimedia component 1 [file mmc1.pdf]

Impact of genetic variants linked to liver fat and liver volume on MRI-mapped body composition

Shafqat Ahmad, Germán D Carrasquilla, Taro Langner, Uwe Menzel, Nouman Ahmad, Sergi Sayols-Baixeras, Koen F Dekkers, Beatrice Kennedy, Filip Malmberg, Ulf Hammar, María J Romero-Lado, Jenny C Censin, Diem Nguyen, Andrés Martínez Mora, Tuomas O Kilpeläinen, Lars Lind, Jan W Eriksson, Robin Strand, Joel Kullberg, Håkan Ahlström, Tove Fall

Table of contents

Supplementary materials and methods……………………………………………………2

Supplementary figures………………………………………………………………………7

Supplementary tables………………………………………………………………….……..27

Supplementary movies……………………………………………………………….……27

Supplementary references…………………………………………………………….…..28

Supplementary materials and methods

Anthropometric and cardiometabolic markers

Height, weight, waist, and hip circumference were objectively measured following standardized protocols. At baseline, body mass index (BMI) was calculated using weight and height (kg/m^2), and waist-hip ratio (WHR) was calculated using waist in centimeters divided by hip in centimeters. Biochemical markers used in this study were assessed in serum samples collected during the baseline assessment. Biochemical measurements were performed using a Beckman Coulter AU5800 analyzer, except for lipoprotein A [LPA, Data-Field 30790], which was measured with a Randox analyzer, and Cystatin C [CYSC, Data-Field 30720], which was measured using a Siemens analyzer.[1] Glycated hemoglobin [HbA1c, Data-Field 30750] was measured from red blood cells using a Bio-Rad Variant II Turbo analyzer.[2]

Clinical diagnosis of MASLD and chronic liver disease

Clinical diagnosis of metabolic dysfunction-associated steatotic liver disease (MASLD) was assessed from health inpatient register diagnoses coded according to the International Classification of Disease version 10 (ICD-10) code K760. Chronic liver disease (CLD) was assessed using ICD-10 K702-704, K717, K721, K74, K740-746, and ICD-9 subtypes (27103; 4562; 571; 5712; 5715; 57150; 57151; 57158; 57159; 5716) which covers an extensive range of liver diseases, including alcohol-induced cirrhosis and primary biliary cirrhosis as has been described previously.[3] The diagnosis of participants, encompassing both prevalent and incident cases, was considered before and after the imaging data.

Liver fat and volume phenotype regression analysis

Before performing the genome-wide association study (GWAS) analysis of liver fat and liver volume, both traits were transformed into inverse-normal traits. In this inverse-normal transformation (INT) two-step approach, the phenotype (trait) was regressed on the covariates in the first step, and the residuals emerging from that linear regression were then inverse-normal

transformed, applying a rank-based algorithm. In the second step, the inverse-normal transformed residuals were regressed on the variant and the covariates to reveal genotype-phenotype associations.

Imiomics analysis

The analysis of the image data with respect to liver fat-associated genetic variant data was completed with an algorithm called "Imiomics".[4] MRI images comprising both water and fat signals for each subject were divided into six stations covering the neck to the knee. The different water and fat signal stations were pre-fused into one neck-to-knee volume per subject, one for the water signal and one for the fat signal. Finally, the water and fat volumes were combined into a fat fraction image, where each voxel represents the relative amount of fat the subject has in that specific region.[4] Here, the fat fraction maps of all subjects were split into man and woman, deformed against a template male subject and a template female subject, respectively. For each subject, a "volume image" or "Jacobian image" with voxel-wise information about the magnitude of the deformation in each region was obtained, together with a "fat fraction image" consisting of the original fat fraction image of each subject deformed into the template subject.[5] The voxels of all the "volume images" and "fat fraction images" were regressed against liver fat- or liver volume-associated single nucleotide polymorphisms (SNPs), adjusting for a set of covariates (age, age squared, total body fat, height, and 20 principal components). These computations provide a map with the voxel-wise regression slope of all regions in the body against the SNPs of interest for men and women and the "volume images" and the "fat fraction images," also known as "Imiomics maps". Consequently, the maps obtained for the volume images show how much the tissue volume across the neck-to-knee region vary by allele in the selected SNPs, while the maps obtained for the fat fraction images depict how much the fat across the neck-to knee region changes by allele of the selected SNPs.

Assessment of alcohol intake

Alcohol intake (measured in grams per week) was assessed through food frequency questionnaires (FFQs). Variables included drinking status (never, previous, current, prefer not to answer) (Data-Field: 20117-0.0) and current alcohol intake (daily or almost daily, three or four times a week, once or twice a week, one to three times a month, special occasion only, never, prefer not to answer) (Data-Field: 1558). Study participants who reported a current alcohol intake frequency of at least once or twice a week were asked to estimate their average weekly alcohol intake of different alcoholic beverages (e.g., red wine, white wine, champagne, beer, cider, spirits, fortified wine) (Data-Fields: 1568, 1578, 1588, 1598, 1608). We then derived the average current intake of alcohol, which was estimated by combining different measures of the alcoholic beverages mentioned above.[6, 7] The FFQs used the following measurements of each of the alcoholic drink types: measures of spirits, glasses for wine, and pints for beer/cider, which are estimated to be equivalent to 1, 2, and 2.5 units, respectively.[6, 7] Study participants who reported a current alcohol intake frequency of "one to three times a month," "special occasions only," or "never" were assumed to have a weekly alcohol consumption volume of zero.[6, 7]

GWAS for liver volume loci without adjustment for height

In the GWAS for liver volume without adjustment for height, we identified the *CENPW* rs1490384 variant (in high linkage disequilibrium (LD), R^2=0.81, D-prime= 0.98 with rs853966 that we discovered in liver volume GWAS with height adjustment), which is a regulatory region variant, and confirmed eight previously identified genetic variants (**Table S5**). The genomic inflation factor was λGC=1.06.

Genetic correlation for liver fat and liver volume across hepatic disease traits

For the assessment of genetic correlations, we utilized linkage disequilibrium score regression (LDSC) analyses, incorporating data from our internal GWAS on liver fat and liver volume as

inputs. We incorporated GWAS data on hepatic diseases from the FinnGen project's Freeze 9 release, dated March 2024 (https://r9.finngen.fi/).[8] These diseases included MASLD, metabolic-associated steatohepatitis (MASH), fibrosis and cirrhosis, and hepatocellular carcinoma. Employing the LDSC method[9] and utilizing the GenomicSEM package version 0.0.5, we estimated genetic correlations between our GWAS on liver fat and liver volume and the aforementioned hepatic diseases. This approach considered disease prevalence data from the FinnGen database and implemented quality control measures to mitigate result inflation. The findings were visualized in a heatmap correlation plot using the ggplot2 package (version 3.4.4) (**Fig. S4**).

Pruning and conditional analysis

We performed linkage disequilibrium (LD) clumping analysis to identify the independent GWAS genetic variants. In the LD clumping approach, two GWAS significant hits approaches were applied (primary with SNPs with p-value $<5\times10^{-08}$ and p-value $<5\times10^{-06}$) using a screening window of 5 Mb adjacent to the first index SNP. In the COJO analysis, we identified genetic regions with one or more SNPs with p-value $<5\times10^{-08}$ using a screening window of 5 Mb adjacent to the first index SNP. New association statistics were calculated for all other SNPs, conditioning on the effect of the index SNP. A SNP with a p-value $<5\times10^{-08}$ conditional on the index SNP was regarded as significantly associated with our outcome trait.

Fine mapping and gene prioritization for liver volume and liver fat loci

In the gene prioritization phase, we focused on the identified significant and independent genetic variants associated with the liver fat and liver volume GWAS, and selected 4 and 10 variants, respectively (**Table 2**). Our prioritization strategies entailed identifying target genes linked to these significant SNPs. These strategies included the retrieval of the nearest candidate gene and the selection of the highest variant-to-gene (V2G) candidate by Open Target Genetics (OTG) utilizing the R package otargen (version 1.1.0),[10] and the implementation of combined

SNP-to-gene strategies (cS2G).[11] The cS2G approach is a composite method integrating seven SNP-to-gene strategies, which involves extensive analysis and fine-mapping to identify putative causal variants within the regions surrounding initial SNP candidates.[11] Fine-mapping using Causal Association and Regression Model Averaging (CARMA) was performed by extending the regions around initial candidate SNPs by ±500,000 bp.[12] After fine mapping, genetic variants with a posterior inclusion probability (PIP) of less than 0.1 were excluded (**Table S2**). This refined set of variants was subjected to further prioritization using the cS2G strategies. The cS2G-reported links were those with a cS2G score greater than 0.5, either with the candidate SNP or a proxy (R^2>0.8), thereby narrowing down to the most likely causal genes. This is why some initially identified variants have more than one cS2G SNP-to-gene link, as all proxy-to-gene links fulfilling the abovementioned conditions were also reported.

Supplementary figures

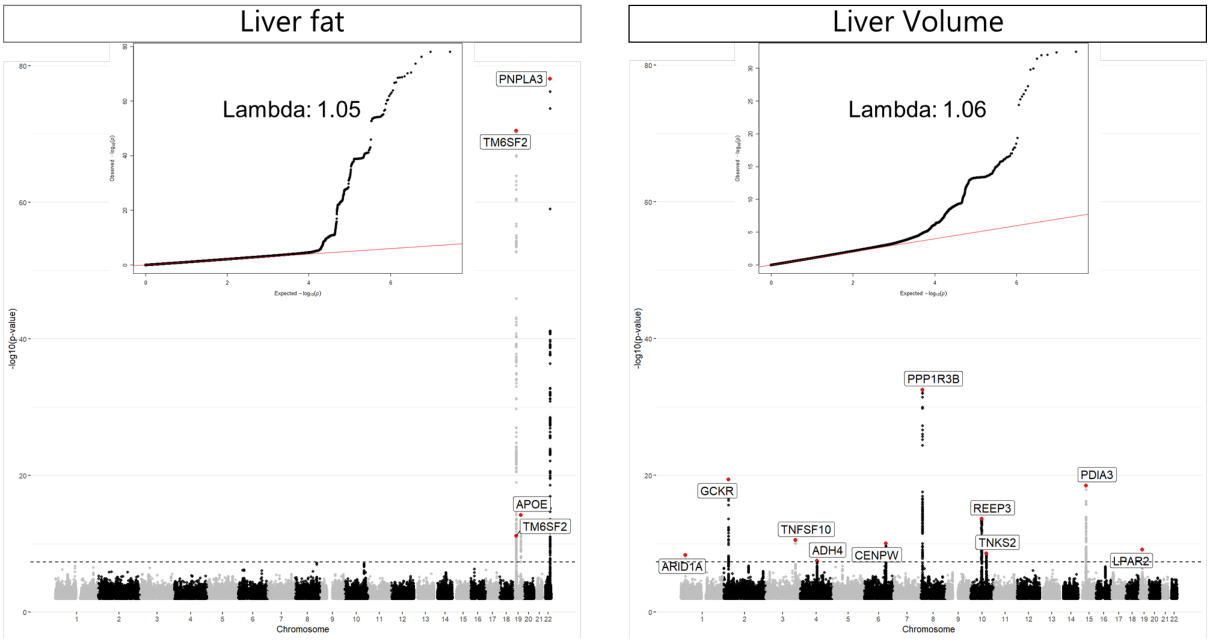

Fig. S1. Manhattan and Q-Q plots from GWAS of liver fat and liver volume in the UK Biobank cohort. Manhattan plot illustrating GWAS of liver fat (n=27,243) and liver volume (n=24,752) in the individuals from the UK Biobank. The left panel presents the Manhattan plot and Q-Q plot of liver fat GWAS, and the right panel presents the Manhattan and Q-Q plot of liver volume GWAS. In the Manhattan plots, the x-axis is the chromosomal position, and the y-axis is the significance of association for each variant in log10(*p*-values). Level of significance is set at *p*-value $<5\times10^{-08}$. The Q-Q plots of GWAS of liver fat and liver volume show the deviation from the diagonal. The genomic inflation factor was ($\lambda GC=1.05$) and ($\lambda GC=1.06$) for the liver fat and liver volume GWAS, respectively.

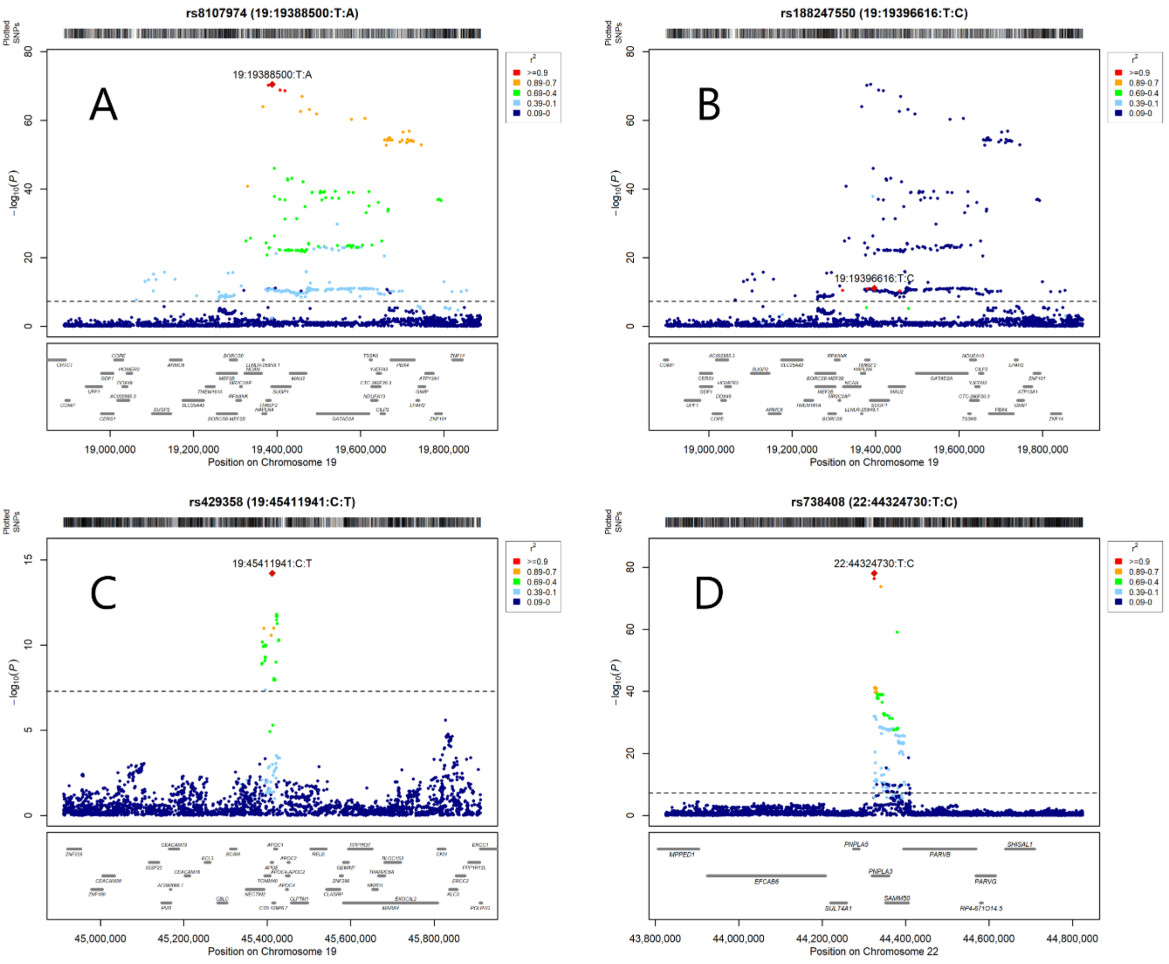

Fig. S2. Regional association plots for liver fat loci. The nearest gene and variant identification are presented for liver fat-associated genetic variants. *TM6SF2* rs8107974 (A), *TM6SF2* rs188247550 (B), *APOE* rs429358 (C), and *PNPLA3* rs738408 (D). The correlation strength between proxies and lead SNPs (R^2) are indicated for each variant in log10(*p*-values) along the chromosome.

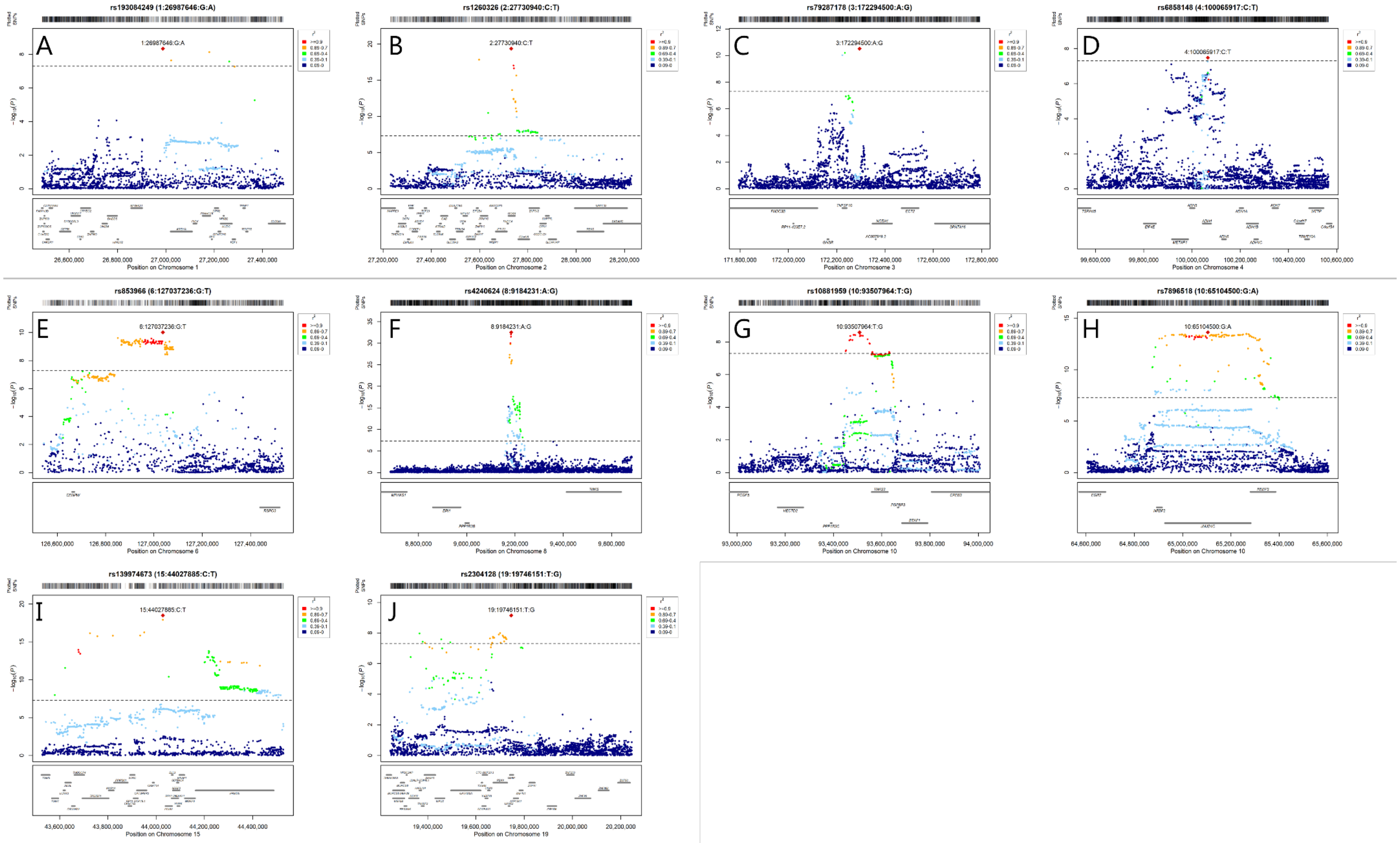

Fig. S3. Regional association plot of liver volume loci. The nearest gene and variant identification are presented for liver volume-associated genetic variants. *ARID1A* rs193084249 (A), *GCKR* rs1260326 (B), *TNFSF10* rs79287178 (C), *ADH4* rs6858148 (D), *CENPW* rs853966 (E), *PPP1R3B* rs4240624 (F), *TNKS2* rs10881959 (G), *REEP3* rs7896518 (H), *PDIA3* rs139974673 (I), and *LPAR2* rs2304128 (J). The correlation strength between proxies and lead SNPs (R^2) are indicated for each variant in log10(*p*-values) along the chromosome.

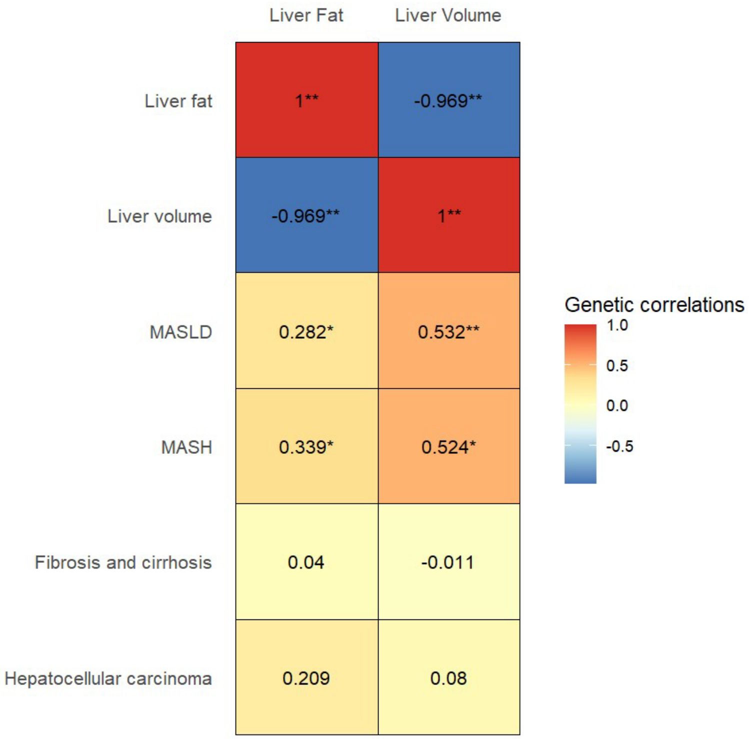

Fig. S4. Genetic correlation for liver fat and liver volume across liver diseases. A single asterisk (*) indicates a significant genetic correlation at *p*-value < 0.05. Double asterisks (**) indicate a significant genetic correlation at the Bonferroni-corrected *p*-value < 0.0042. Blue shading represents negative genetic correlations, and red shading represents positive correlations, with increasing color intensity reflecting increasing correlation strength.

Gene	*TM6SF2* (rs8107974)				*TM6SF2* (rs188247550)				*APOE* (rs429358)				*PNPLA3* (rs738408)			
**Volume or fat fraction**	**Volume**		**Fat fraction**		**Volume**		**Fat fraction**		**Volume**		**Fat fraction**		**Volume**		**Fat fraction**	
**sex**	**Men**	**Women**	**Men**	**Women**	**Men**	**Women**	**Men**	**Women**	**Men**	**Women**	**Men**	**Women**	**Men**	**Women**	**Men**	**Women**
**Adipose tissue**																
Thorax SAT		+ *														
Abdominal anterior SAT		+ *														
Abdominal posterior SAT		- *														
VAT																
Gluteal SAT		- *				- *								+ *		
Thigh SAT	-- *					--*							--*			
**Organs**																
Lungs																
Heart																
Liver			+++	++			+	+			+	+			++	++
Kidneys																
Other																
**Skeleton**																
Spine																
Pelvis																
Femur																
**Skeletal muscle**																
Thorax																
Abdomen																
Gluteus																
Thigh										+ *						
**Specific notes and captions**	* Regional				* Regional				* Regional				* Regional			

Color scale

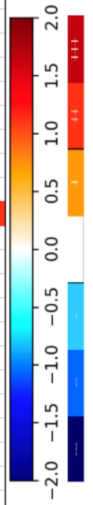

Fig. S5. Summary findings for liver fat genetic variants across neck-to-knee voxel maps for tissue volume and fat fraction. The nearest gene and the variant identification are presented. Tissue volume and fat fraction are presented separately for men and women for different parts of the body. SAT, subcutaneous adipose tissue. The +, ++, +++ and -- indicate the effect size. The * indicates regional associations. Significant associations (p-value $<2.8\times10^{-3}$) are color coded from negative (blue) to positive (red).

Gene	*ARID1A* (rs193084249)				*GCKR* (rs1260326)				*TNFSF10* (rs79287178)				*ADH4* (rs6858148)				*CENPW* (rs853966)			
Volume or fat content	Volume		Fat fraction		Volume		Fat fraction		Volume		Fat fraction		Volume		Fat fraction		Volume		Fat fraction	
Sex	Men	Women	Men	Women	Men	Women	Men	Women	Men	Women	Men	Women	Men	Women	Men	Women	Men	Women	Men	Women
**Adipose tissue**																				
Thorax SAT																				
Abdominal anterior SAT																				
Abdominal posterior SAT																				
VAT														++*						
Gluteal SAT																				
Thigh SAT					–*															
**Organs**																				
Lungs																				
Heart										+*								+*		
Liver	+*				++		+*		+*	+*			+*					+*		
Kidneys																				
**Skeleton**																				
Spine																–*			-*	
Pelvis																			-*	
Femur																			–*	-*
**Skeletal muscle**																				
Thorax						–*											+*			
Abdomen													+*				+*			
Gluteus						–*		+*									++*	+*		
Thigh						–*				+*							+*	+*		
**Specific notes and captions**	* Regional				* Regional		* Regional		* Regional				* Regional		* Discs		* Regional		* Regional	

Color scale

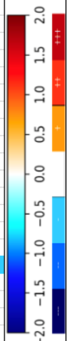

Gene	*PPP1R3B* (rs4240624)				*TNKS2* (rs10881959)				*REEP3* (rs7896518)				*PDIA3* (rs139974673)				*LPAR2* (rs2304128)			
Volume or fat content	Volume		Fat fraction		Volume		Fat fraction		Volume		Fat fraction		Volume		Fat fraction		Volume		Fat fraction	
Sex	Men	Women	Men	Women	Men	Women	Men	Women	Men	Women	Men	Women	Men	Women	Men	Women	Men	Women	Men	Women
**Adipose tissue**																				
Thorax SAT																	+*			
Abdominal anterior SAT																		+*		
Abdominal posterior SAT																				
VAT																				
Gluteal SAT														-*						
Thigh SAT																				
**Organs**																				
Lungs																				
Heart									++				+*							
Liver	++	++			++*				++	++			++	++*		+			++	++
Kidneys																				
**Skeleton**																				
Spine																				
Pelvis																				
Femur																				
**Skeletal muscle**																				
Thorax	+*																			
Abdomen																				
Gluteus	+*																			
Thigh																				
**Specific notes and captions**	* Regional				* Regional								* Regional				* Regional			

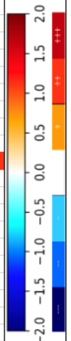

Fig. S6. Summary of findings for liver volume genetic variants across neck-to-knee voxel maps for tissue volume and fat fraction. The nearest gene and the variant identification are presented. Tissue volume and fat fraction are presented separately for men and women for different parts of the body. SAT, subcutaneous adipose tissue. The +, ++, +++ and -- indicate the effect size. The * indicate regional associations. Significant associations (p-value $<2.8\times10^{-3}$) are color coded from negative (blue) to positive (red).

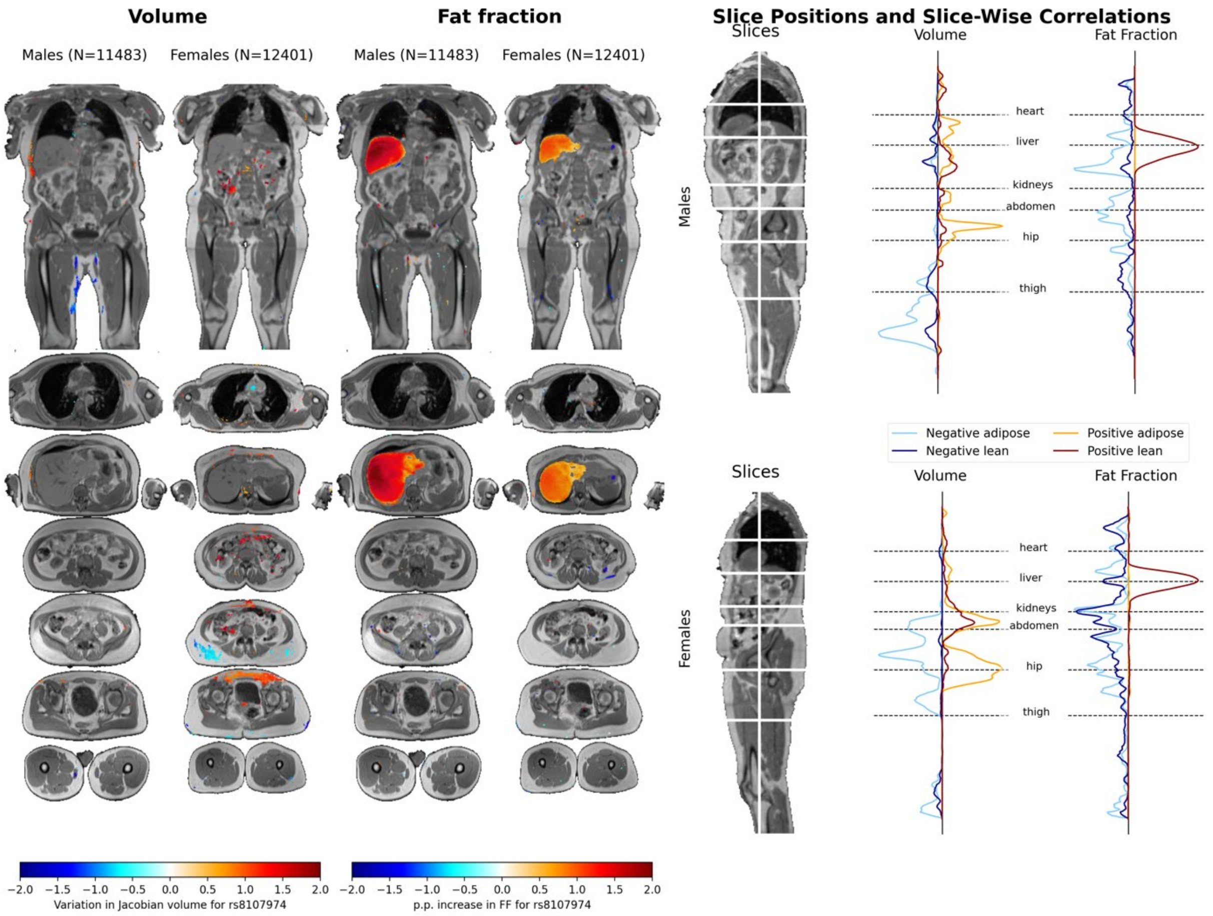

Fig. S7. Imiomics maps for the *TM6SF2* rs8107974 variant that quantifies its association to tissue volume and fat fraction voxel-wise throughout the neck-to-knee region. The resulting images show, from left to right, the associations to volume for males, the associations to volume for females, the associations to fat fractions for males, and the associations to fat fractions for females. Color mapping is used to visualize the quantified associations where significant (*p*-value $<2.8 \times 10^{-3}$). For visualization purposes clipped beta values (from the lower 1% to the highest 1%) were for each experiment linearly rescaled between -2 to +2 maintaining the sign of the association. Non-significant regions are not colored; but instead, a mix of the water and fat magnetic resonance images for the chosen male and female template subjects are shown to allow orientation in the body regions. The first row of images represents coronal slices of the maps, while the remaining rows represent axial slices of those maps in levels of locations of interest (heart, liver, kidneys, abdomen, hip, and thighs). The mid-sagittal slices on the right-hand side of the plots illustrate the visualized slices. The plots on the right-hand side of the collage figures represent the relative amount, from neck to knee, of significant positive and negative associations in the 3D data. The associations are reported separately from lean tissues (where the water signal is stronger than the fat signal) and from adipose tissue (where the fat signal is stronger than the water signal). Full 3D associations are visualized in **Movie S1** (axial and coronal planes).

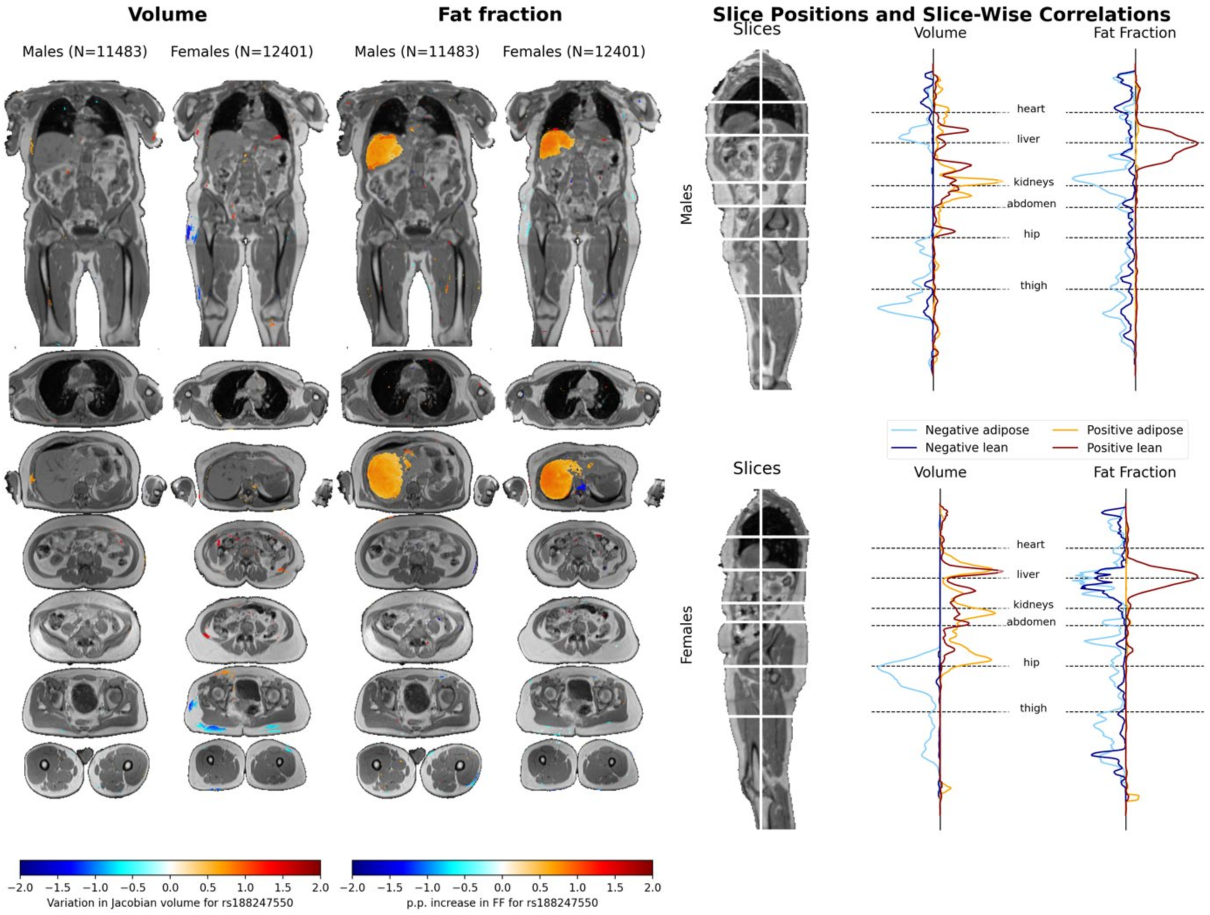

Fig. S8. Imiomics maps for the *TM6SF2* rs188247550 variant that quantifies its association to tissue volume and fat fraction voxel-wise throughout the neck-to-knee region. The resulting images show, from left to right, the associations to volume for males, the associations to volume for females, the associations to fat fractions for males, and the associations to fat fractions for females. Color mapping is used to visualize the quantified associations where significant (p-value $<2.8x10^{-3}$). For visualization purposes clipped beta values (from the lower 1% to the highest 1%) were for each experiment linearly rescaled between -2 to +2 maintaining the sign of the association. Non-significant regions are not colored; but instead, a mix of the water and fat magnetic resonance images for the chosen male and female template subjects are shown to allow orientation in the body regions. The first row of images represents coronal slices of the maps, while the remaining rows represent axial slices of those maps in levels of locations of interest (heart, liver, kidneys, abdomen, hip, and thighs). The mid-sagittal slices on the right-hand side of the plots illustrate the visualized slices. The plots on the right-hand side of the collage figures represent the relative amount, from neck to knee, of significant positive and negative associations in the 3D data. The associations are reported separately from lean tissues (where the water signal is stronger than the fat signal) and from adipose tissue (where the fat signal is stronger than the water signal). Full 3D associations are visualized in **Movie S2** (axial and coronal planes).

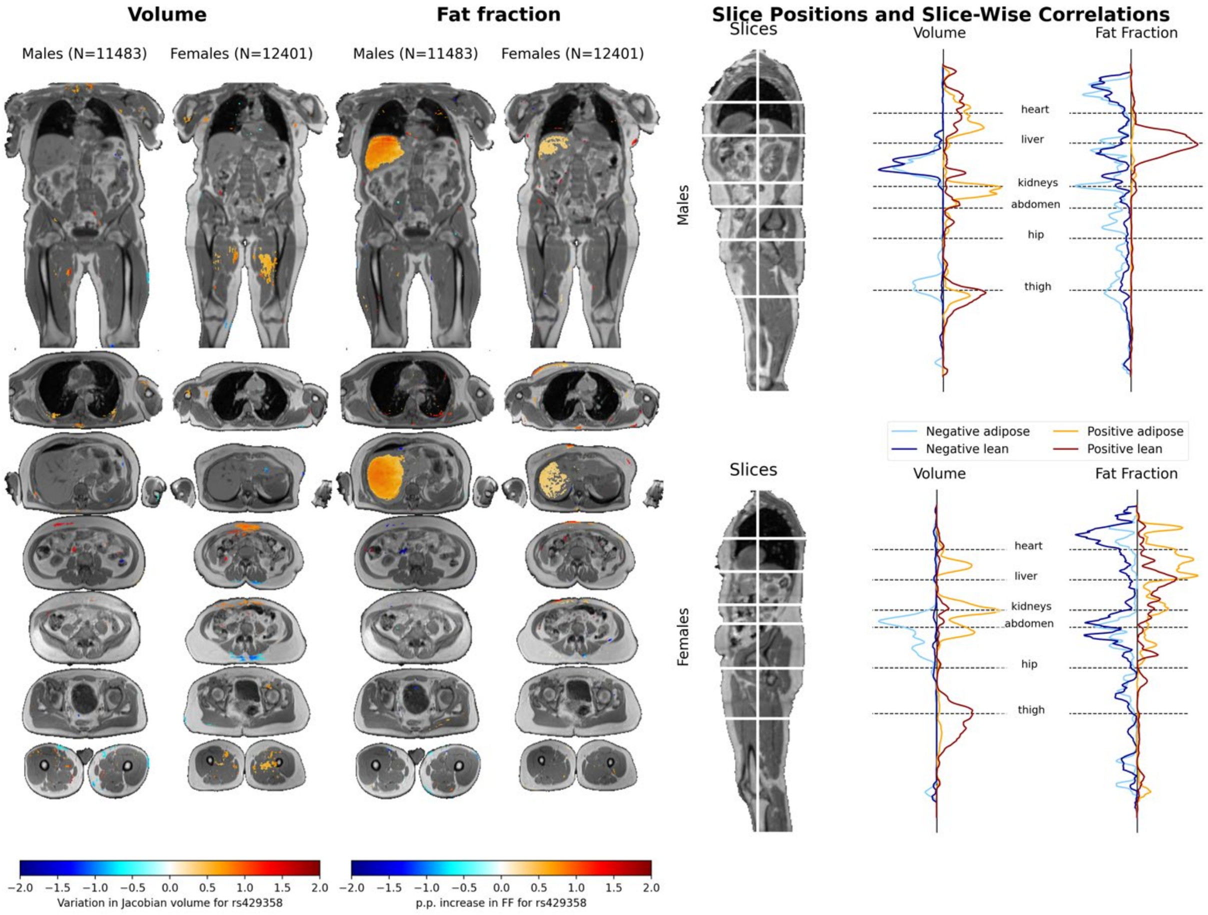

Fig. S9. Imiomics maps for the *APOE* rs429358 variant that quantifies its association to tissue volume and fat fraction voxel-wise throughout the neck-to-knee region. The resulting images show, from left to right, the associations to volume for males, the associations to volume for females, the associations to fat fractions for males, and the associations to fat fractions for females. Color mapping is used to visualize the quantified associations where significant (p-value $<2.8 \times 10^{-3}$). For visualization purposes clipped beta values (from the lower 1% to the highest 1%) were for each experiment linearly rescaled between -2 to +2 maintaining the sign of the association. Non-significant regions are not colored; but instead, a mix of the water and fat magnetic resonance images for the chosen male and female template subjects are shown to allow orientation in the body regions. The first row of images represents coronal slices of the maps, while the remaining rows represent axial slices of those maps in levels of locations of interest (heart, liver, kidneys, abdomen, hip, and thighs). The mid-sagittal slices on the right-hand side of the plots illustrate the visualized slices. The plots on the right-hand side of the collage figures represent the relative amount, from neck to knee, of significant positive and negative associations in the 3D data. The associations are reported separately from lean tissues (where the water signal is stronger than the fat signal) and from adipose tissue (where the fat signal is stronger than the water signal). Full 3D associations are visualized in **Movie S3** (axial and coronal planes).

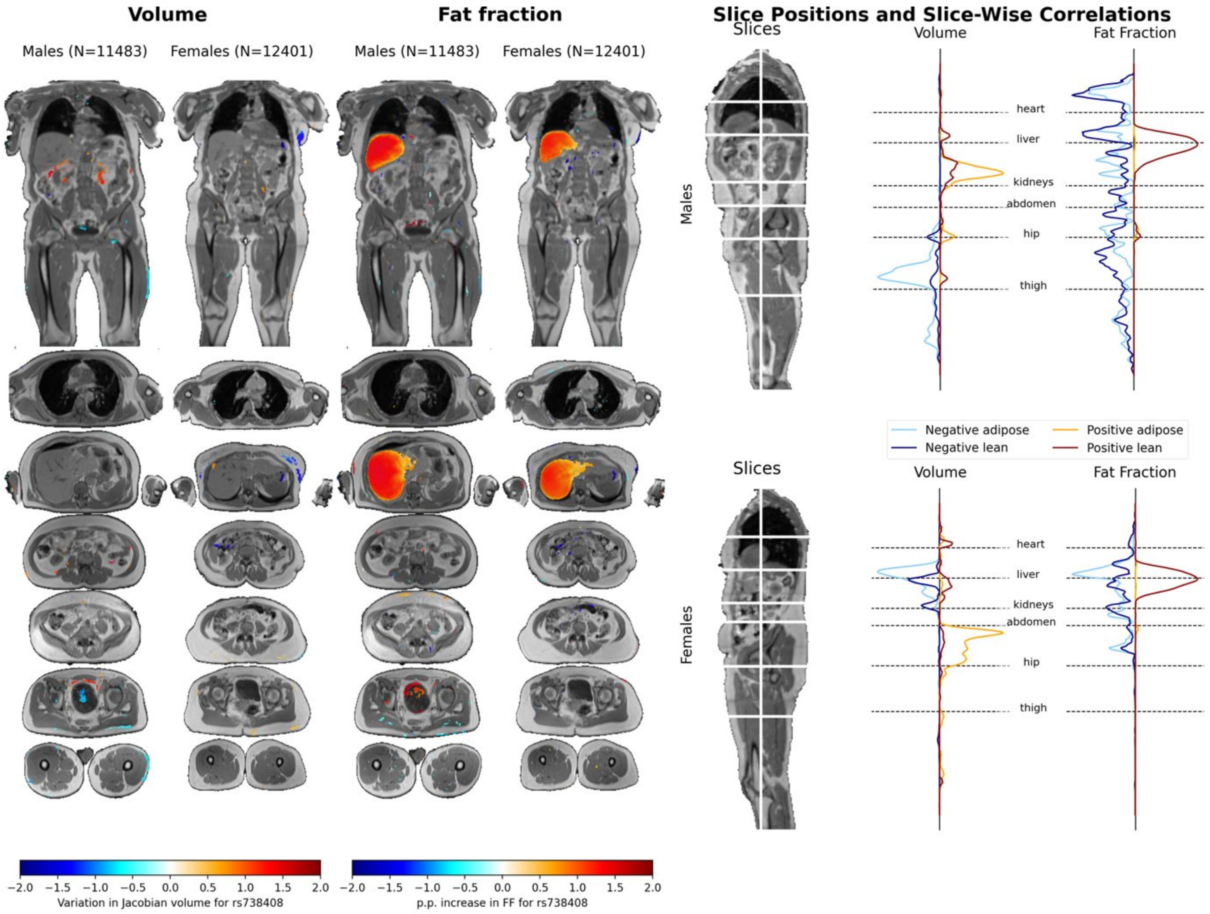

Fig. S10. Imiomics maps for the *PNPLA3* rs738408 variant that quantifies its association to tissue volume and fat fraction voxel-wise throughout the neck-to-knee region. The resulting images show, from left to right, the associations to volume for males, the associations to volume for females, the associations to fat fractions for males, and the associations to fat fractions for females. Color mapping is used to visualize the quantified associations where significant (p-value <2.8×10^{-3}). For visualization purposes clipped beta values (from the lower 1% to the highest 1%) were for each experiment linearly rescaled between -2 to +2 maintaining the sign of the association. Non-significant regions are not colored; but instead, a mix of the water and fat magnetic resonance images for the chosen male and female template subjects are shown to allow orientation in the body regions. The first row of images represents coronal slices of the maps, while the remaining rows represent axial slices of those maps in levels of locations of interest (heart, liver, kidneys, abdomen, hip, and thighs). The mid-sagittal slices on the right-hand side of the plots illustrate the visualized slices. The plots on the right-hand side of the collage figures represent the relative amount, from neck to knee, of significant positive and negative associations in the 3D data. The associations are reported separately from lean tissues (where the water signal is stronger than the fat signal) and from adipose tissue (where the fat signal is stronger than the water signal). Full 3D associations are visualized in **Movie S4** (axial and coronal planes).

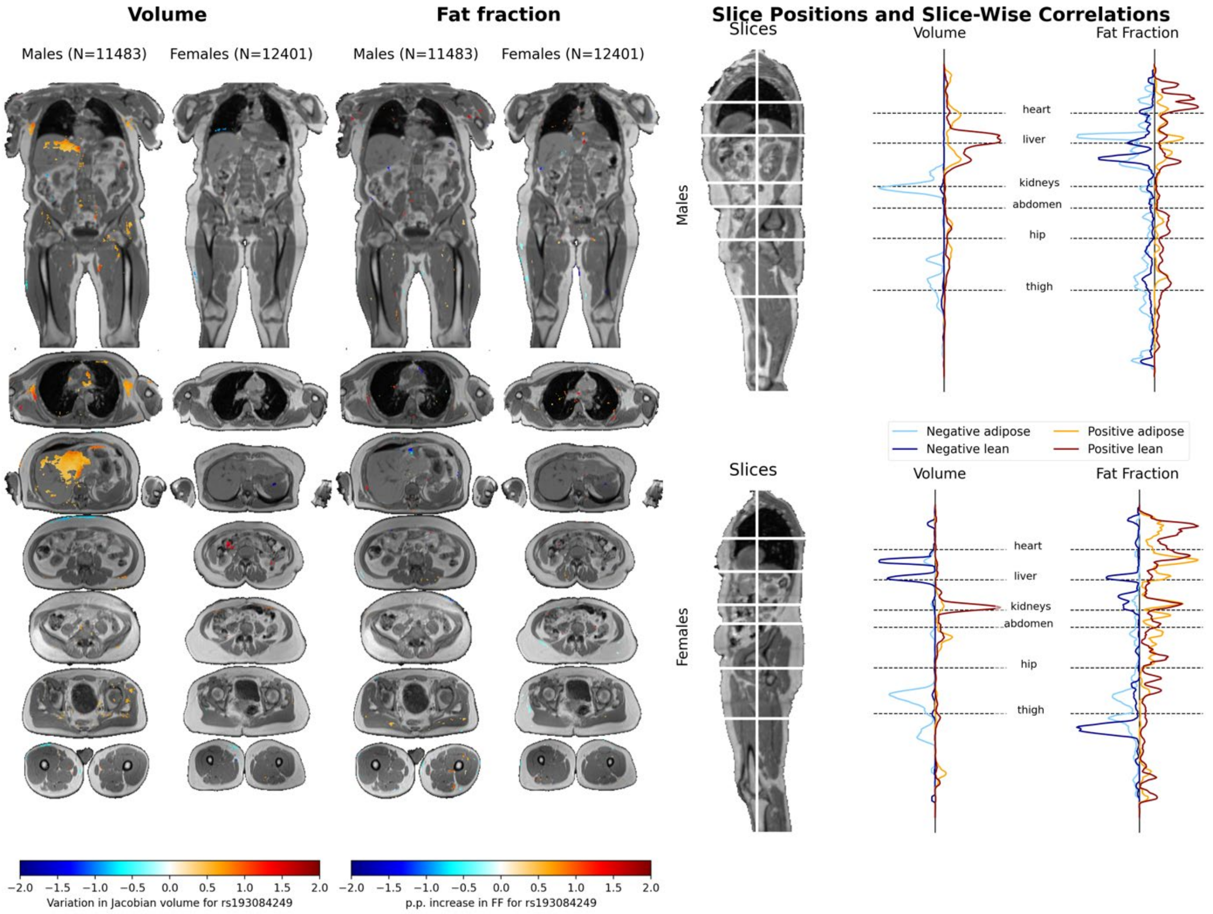

Fig. S11. Imiomics maps for the *ARID1A* rs193084249 variant that quantifies its association to tissue volume and fat fraction voxel-wise throughout the neck-to-knee region. The resulting images show, from left to right, the associations to volume for males, the associations to volume for females, the associations to fat fractions for males, and the associations to fat fractions for females. Color mapping is used to visualize the quantified associations where significant (p-value $<2.8\text{x}10^{-3}$). For visualization purposes clipped beta values (from the lower 1% to the highest 1%) were for each experiment linearly rescaled between -2 to +2 maintaining the sign of the association. Non-significant regions are not colored; but instead, a mix of the water and fat magnetic resonance images for the chosen male and female template subjects are shown to allow orientation in the body regions. The first row of images represents coronal slices of the maps, while the remaining rows represent axial slices of those maps in levels of locations of interest (heart, liver, kidneys, abdomen, hip, and thighs). The mid-sagittal slices on the right-hand side of the plots illustrate the visualized slices. The plots on the right-hand side of the collage figures represent the relative amount, from neck to knee, of significant positive and negative associations in the 3D data. The associations are reported separately from lean tissues (where the water signal is stronger than the fat signal) and from adipose tissue (where the fat signal is stronger than the water signal). Full 3D associations are visualized in **Movie S5** (axial and coronal planes).

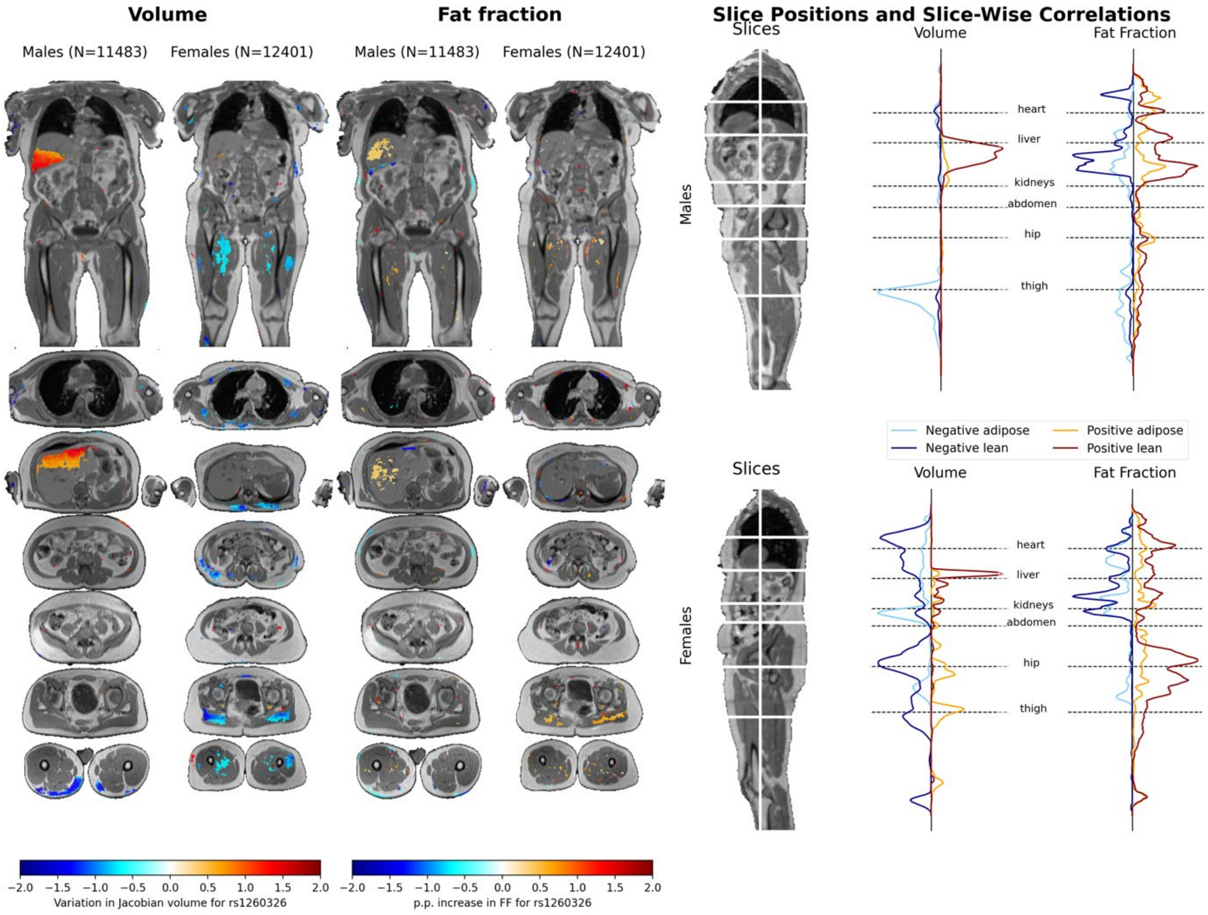

Fig. S12. Imiomics maps for the *GCKR* rs1260326 variant that quantifies its association to tissue volume and fat fraction voxel-wise throughout the neck-to-knee region. The resulting images show, from left to right, the associations to volume for males, the associations to volume for females, the associations to fat fractions for males, and the associations to fat fractions for females. Color mapping is used to visualize the quantified associations where significant (*p*-value $<2.8 \times 10^{-3}$ For visualization purposes clipped beta values (from the lower 1% to the highest 1%) were for each experiment linearly rescaled between -2 to +2 maintaining the sign of the association. Non-significant regions are not colored; but instead, a mix of the water and fat magnetic resonance images for the chosen male and female template subjects are shown to allow orientation in the body regions. The first row of images represents coronal slices of the maps, while the remaining rows represent axial slices of those maps in levels of locations of interest (heart, liver, kidneys, abdomen, hip, and thighs). The mid-sagittal slices on the right-hand side of the plots illustrate the visualized slices. The plots on the right-hand side of the collage figures represent the relative amount, from neck to knee, of significant positive and negative associations in the 3D data. The associations are reported separately from lean tissues (where the water signal is stronger than the fat signal) and from adipose tissue (where the fat signal is stronger than the water signal). Full 3D associations are visualized in **Movie S6** (axial and coronal planes).

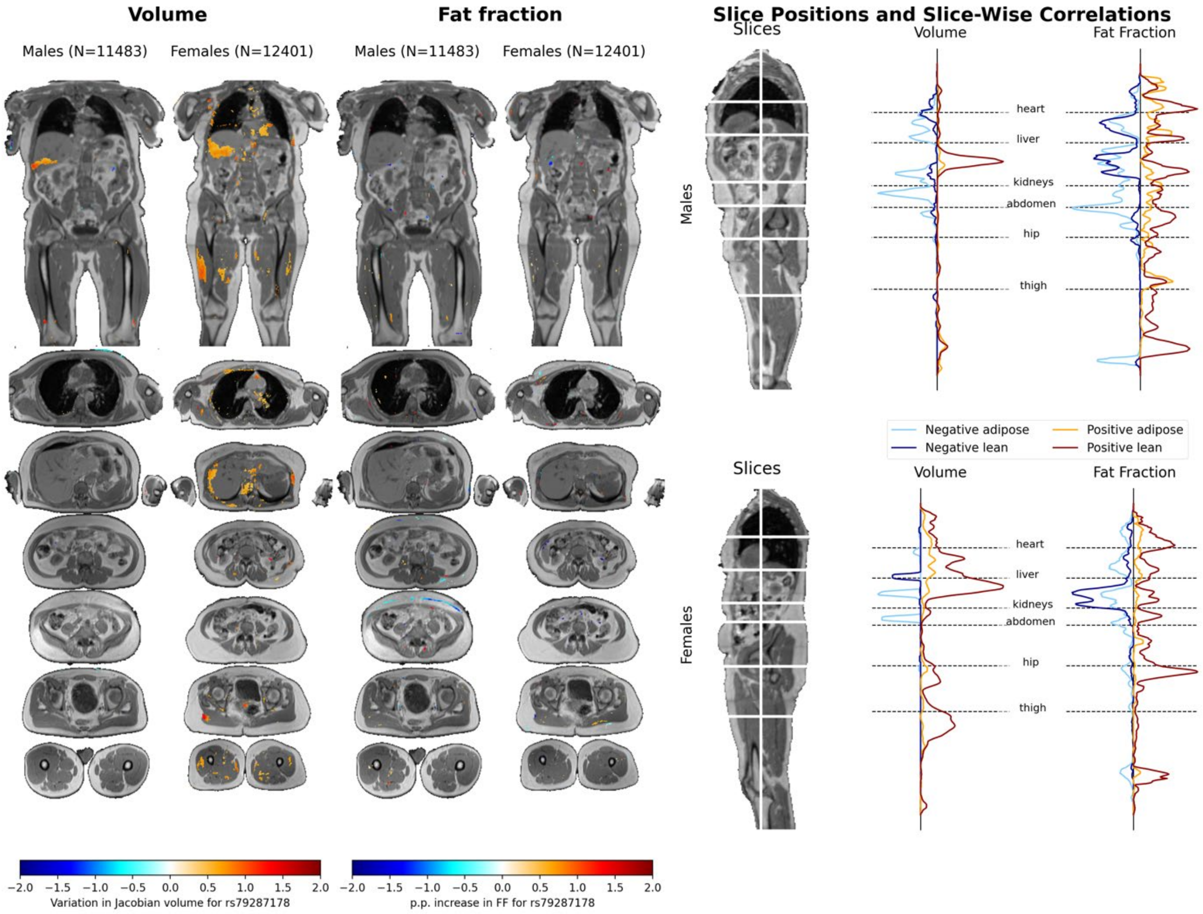

Fig. S13. Imiomics maps for the *TNFSF10* rs79287178 variant that quantifies its association to tissue volume and fat fraction voxel-wise throughout the neck-to-knee region. The resulting images show, from left to right, the associations to volume for males, the associations to volume for females, the associations to fat fractions for males, and the associations to fat fractions for females. Color mapping is used to visualize the quantified associations where significant (p-value $<2.8\text{x}10^{-3}$). For visualization purposes clipped beta values (from the lower 1% to the highest 1%) were for each experiment linearly rescaled between -2 to +2 maintaining the sign of the association. Non-significant regions are not colored; but instead, a mix of the water and fat magnetic resonance images for the chosen male and female template subjects are shown to allow orientation in the body regions. The first row of images represents coronal slices of the maps, while the remaining rows represent axial slices of those maps in levels of locations of interest (heart, liver, kidneys, abdomen, hip, and thighs). The mid-sagittal slices on the right-hand side of the plots illustrate the visualized slices. The plots on the right-hand side of the collage figures represent the relative amount, from neck to knee, of significant positive and negative associations in the 3D data. The associations are reported separately from lean tissues (where the water signal is stronger than the fat signal) and from adipose tissue (where the fat signal is stronger than the water signal). Full 3D associations are visualized in **Movie S7** (axial and coronal planes).

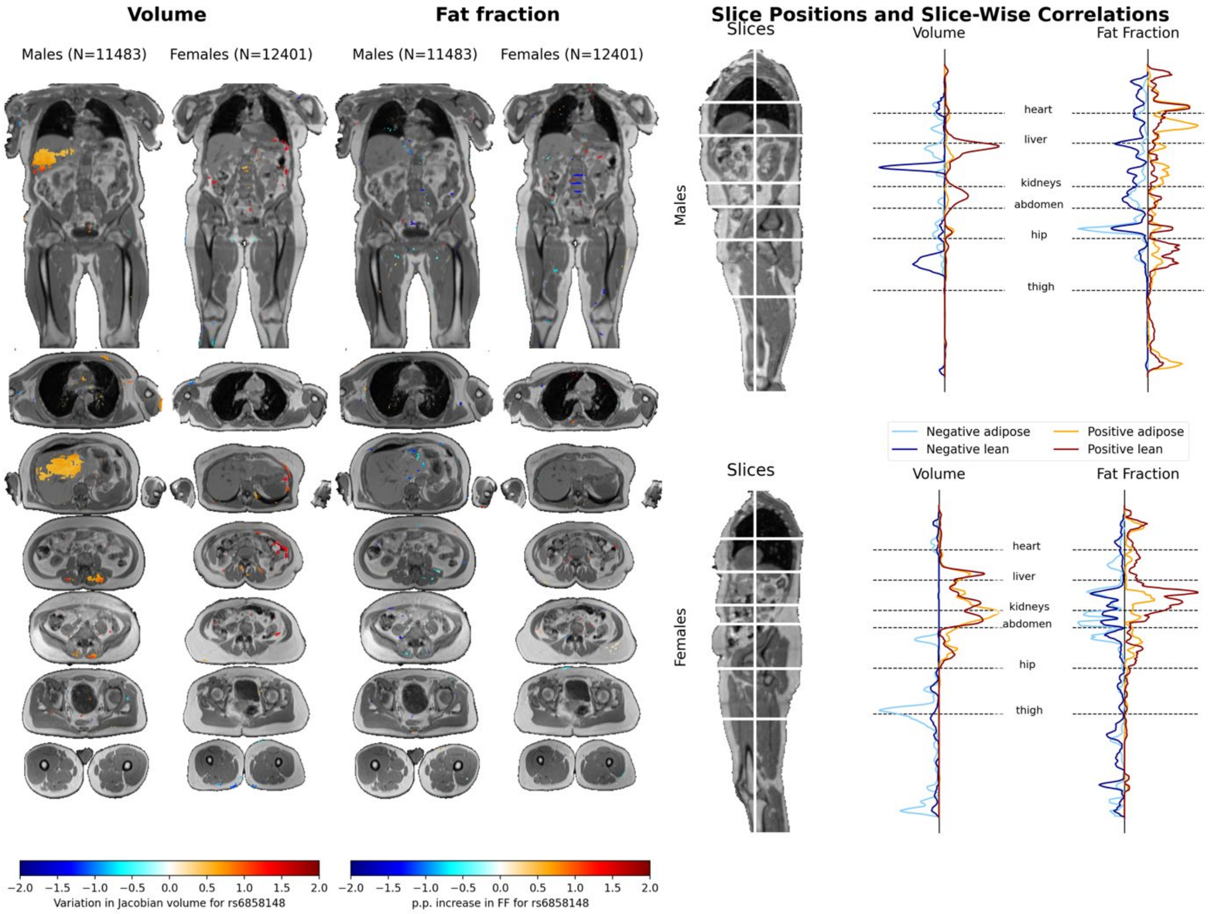

Fig. S14. Imiomics maps for the *ADH4* rs6858148 variant that quantifies its association to tissue volume and fat fraction voxel-wise throughout the neck-to-knee region. The resulting images show, from left to right, the associations to volume for males, the associations to volume for females, the associations to fat fractions for males, and the associations to fat fractions for females. Color mapping is used to visualize the quantified associations where significant (p-value <2.8×10^{-3}). For visualization purposes clipped beta values (from the lower 1% to the highest 1%) were for each experiment linearly rescaled between -2 to +2 maintaining the sign of the association. Non-significant regions are not colored; but instead, a mix of the water and fat magnetic resonance images for the chosen male and female template subjects are shown to allow orientation in the body regions. The first row of images represents coronal slices of the maps, while the remaining rows represent axial slices of those maps in levels of locations of interest (heart, liver, kidneys, abdomen, hip, and thighs). The mid-sagittal slices on the right-hand side of the plots illustrate the visualized slices. The plots on the right-hand side of the collage figures represent the relative amount, from neck to knee, of significant positive and negative associations in the 3D data. The associations are reported separately from lean tissues (where the water signal is stronger than the fat signal) and from adipose tissue (where the fat signal is stronger than the water signal). Full 3D associations are visualized in **Movie S8** (axial and coronal planes).

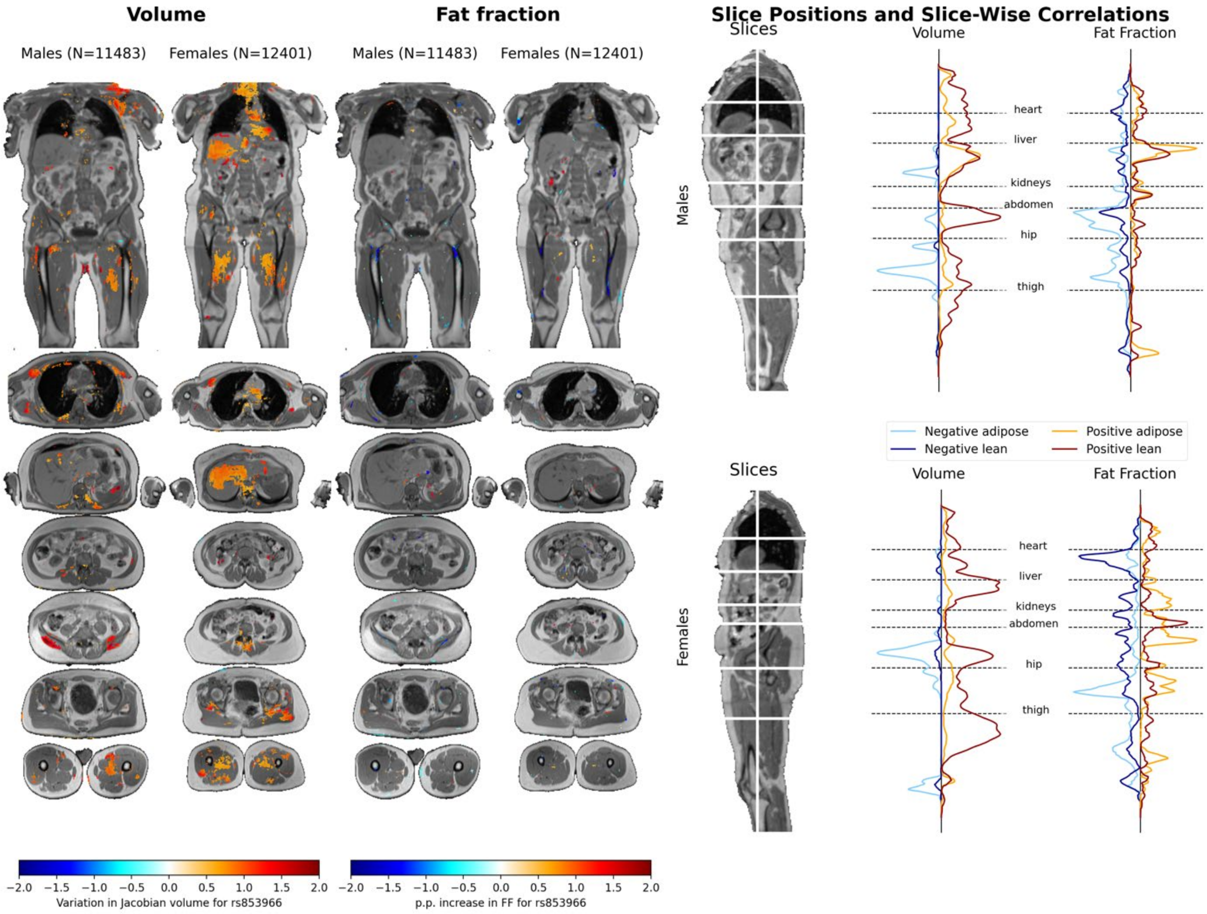

Fig. S15. Imiomics maps for the *CENPW* rs853966 variant that quantifies its association to tissue volume and fat fraction voxel-wise throughout the neck-to-knee region. The resulting images show, from left to right, the associations to volume for males, the associations to volume for females, the associations to fat fractions for males, and the associations to fat fractions for females. Color mapping is used to visualize the quantified associations where significant (p-value $<2.8\text{x}10^{-3}$). For visualization purposes clipped beta values (from the lower 1% to the highest 1%) were for each experiment linearly rescaled between -2 to +2 maintaining the sign of the association. Non-significant regions are not colored; but instead, a mix of the water and fat magnetic resonance images for the chosen male and female template subjects are shown to allow orientation in the body regions. The first row of images represents coronal slices of the maps, while the remaining rows represent axial slices of those maps in levels of locations of interest (heart, liver, kidneys, abdomen, hip, and thighs). The mid-sagittal slices on the right-hand side of the plots illustrate the visualized slices. The plots on the right-hand side of the collage figures represent the relative amount, from neck to knee, of significant positive and negative associations in the 3D data. The associations are reported separately from lean tissues (where the water signal is stronger than the fat signal) and from adipose tissue (where the fat signal is stronger than the water signal). Full 3D associations are visualized in **Movie S9** (axial and coronal planes).

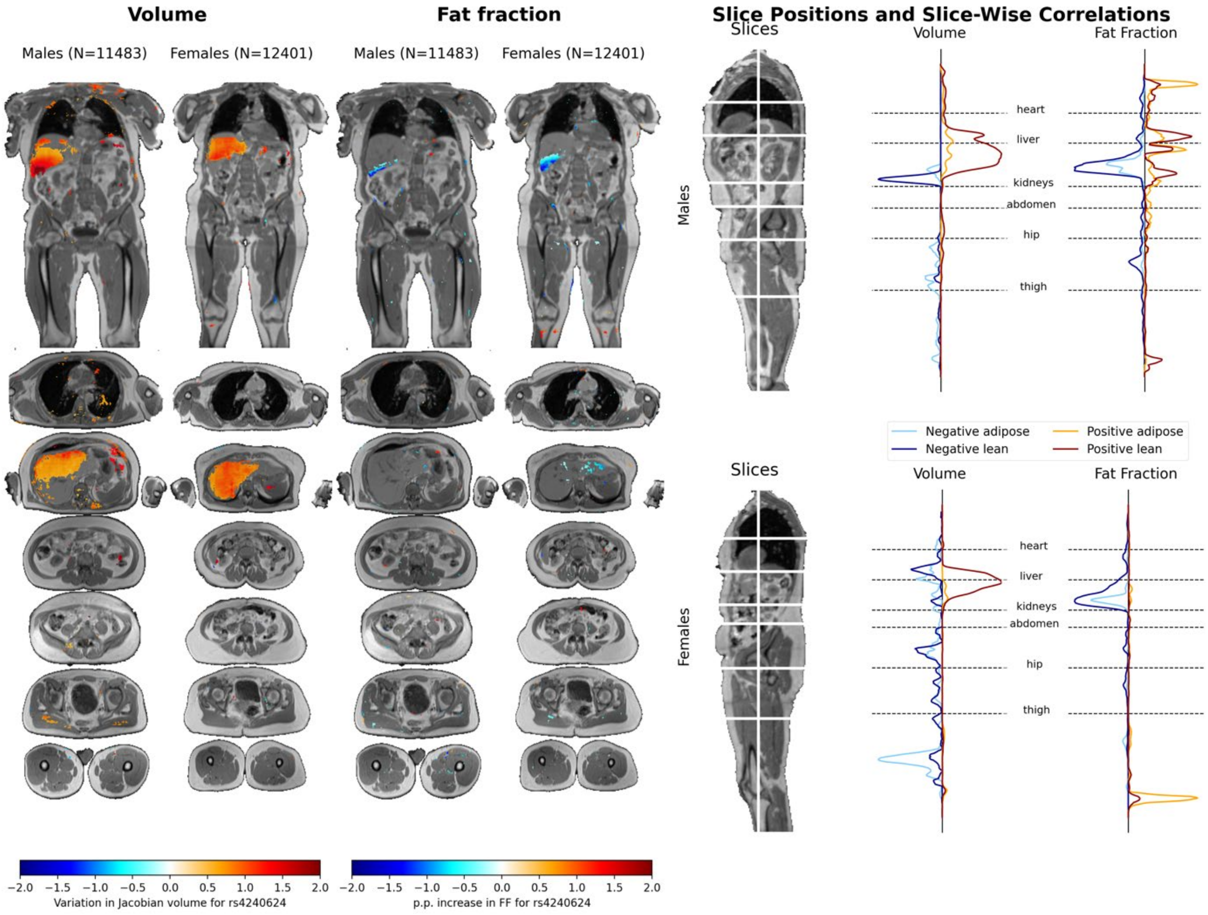

Fig. S16. Imiomics maps for the *PPP1R3B* rs4240624 variant that quantifies its association to tissue volume and fat fraction voxel-wise throughout the neck-to-knee region. The resulting images show, from left to right, the associations to volume for males, the associations to volume for females, the associations to fat fractions for males, and the associations to fat fractions for females. Color mapping is used to visualize the quantified associations where significant (p-value $<2.8\text{x}10^{-3}$). For visualization purposes clipped beta values (from the lower 1% to the highest 1%) were for each experiment linearly rescaled between -2 to +2 maintaining the sign of the association. Non-significant regions are not colored; but instead, a mix of the water and fat magnetic resonance images for the chosen male and female template subjects are shown to allow orientation in the body regions. The first row of images represents coronal slices of the maps, while the remaining rows represent axial slices of those maps in levels of locations of interest (heart, liver, kidneys, abdomen, hip, and thighs). The mid-sagittal slices on the right-hand side of the plots illustrate the visualized slices. The plots on the right-hand side of the collage figures represent the relative amount, from neck to knee, of significant positive and negative associations in the 3D data. The associations are reported separately from lean tissues (where the water signal is stronger than the fat signal) and from adipose tissue (where the fat signal is stronger than the water signal). Full 3D associations are visualized in **Movie S10** (axial and coronal planes).

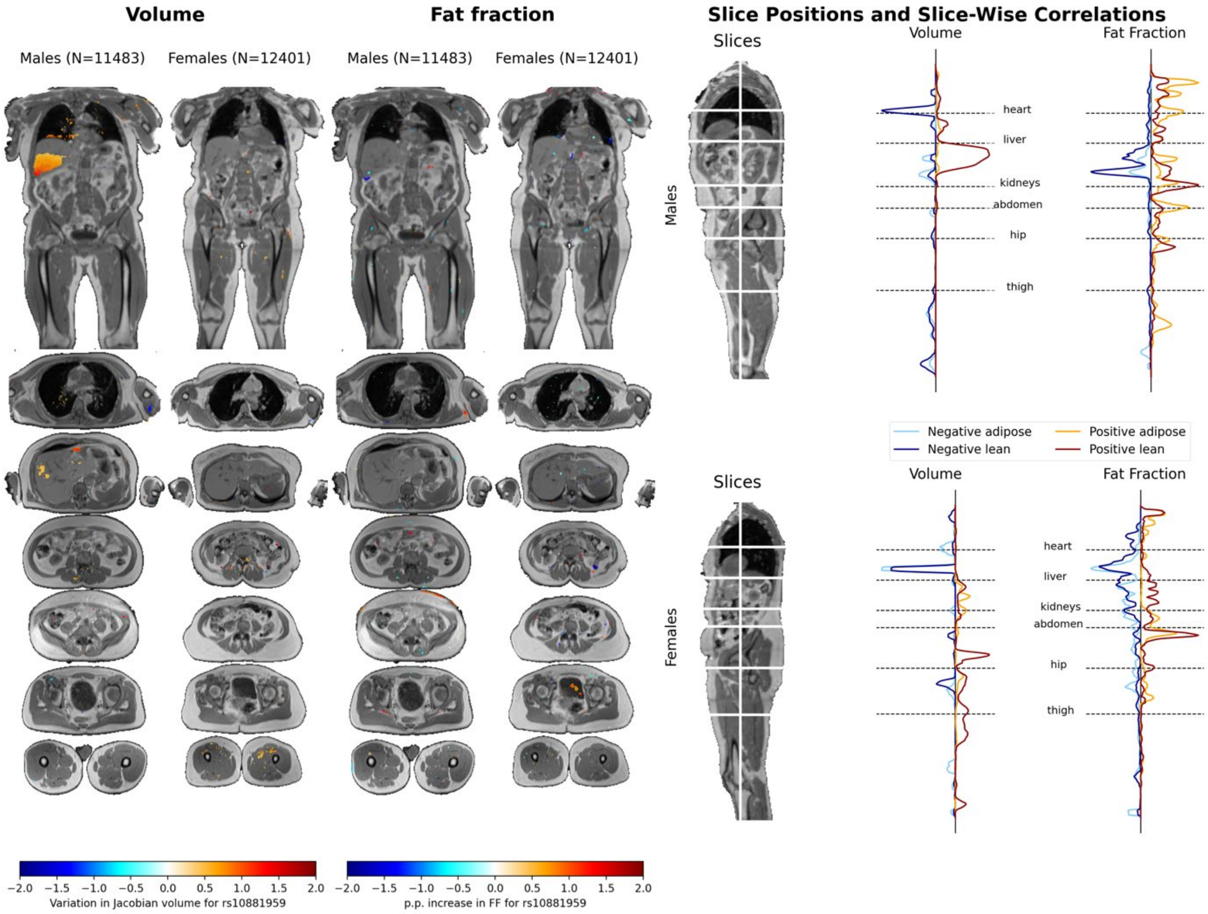

Fig. S17. Imiomics maps for the *TNKS2* rs10881959 variant that quantifies its association to tissue volume and fat fraction voxel-wise throughout the neck-to-knee region. The resulting images show, from left to right, the associations to volume for males, the associations to volume for females, the associations to fat fractions for males, and the associations to fat fractions for females. Color mapping is used to visualize the quantified associations where significant (p-value $<2.8\text{x}10^{-3}$). For visualization purposes clipped beta values (from the lower 1% to the highest 1%) were for each experiment linearly rescaled between -2 to +2 maintaining the sign of the association. Non-significant regions are not colored; but instead, a mix of the water and fat magnetic resonance images for the chosen male and female template subjects are shown to allow orientation in the body regions. The first row of images represents coronal slices of the maps, while the remaining rows represent axial slices of those maps in levels of locations of interest (heart, liver, kidneys, abdomen, hip, and thighs). The mid-sagittal slices on the right-hand side of the plots illustrate the visualized slices. The plots on the right-hand side of the collage figures represent the relative amount, from neck to knee, of significant positive and negative associations in the 3D data. The associations are reported separately from lean tissues (where the water signal is stronger than the fat signal) and from adipose tissue (where the fat signal is stronger than the water signal). Full 3D associations are visualized in **Movie S11** (axial and coronal planes).

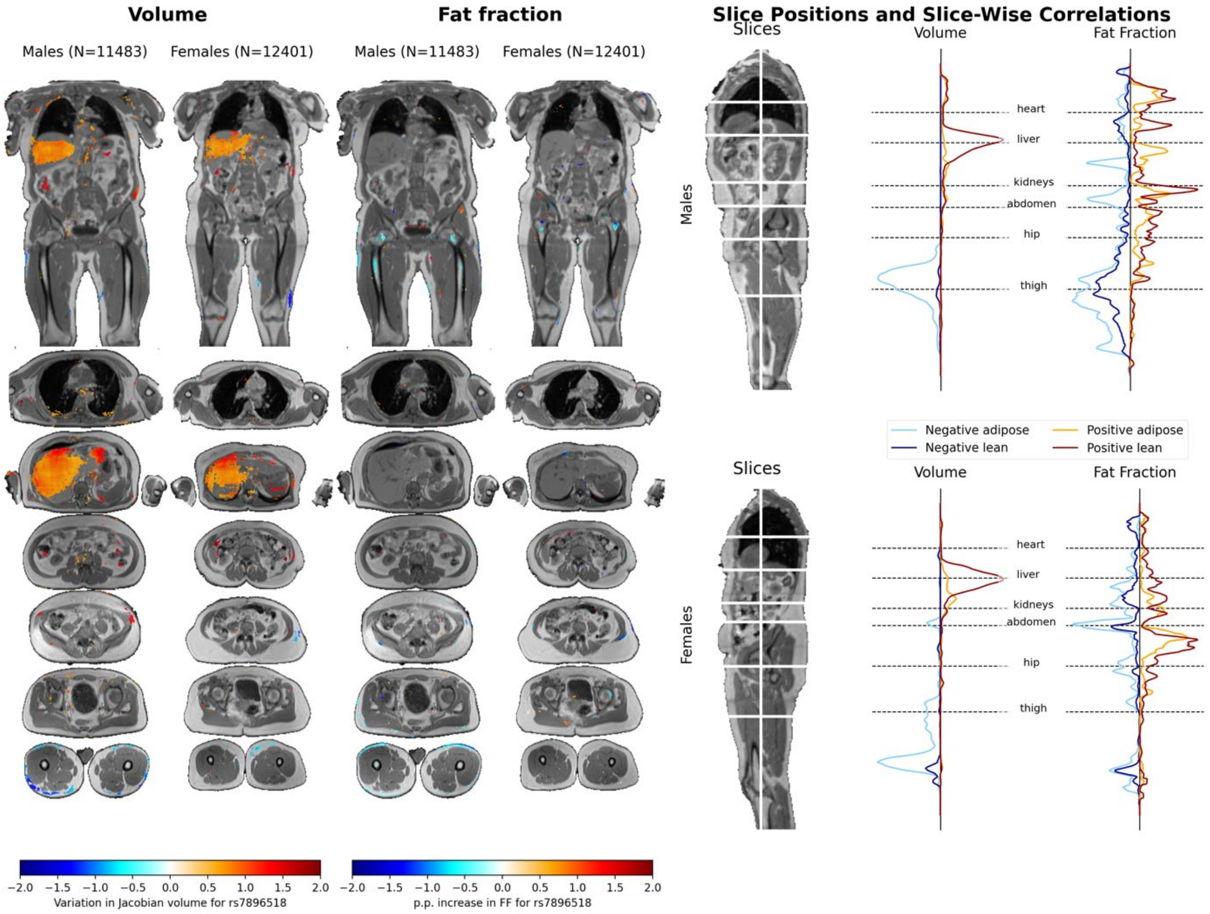

Fig. S18. Imiomics maps for the *REEP3* rs7896518 variant that quantifies its association to tissue volume and fat fraction voxel-wise throughout the neck-to-knee region. The resulting images show, from left to right, the associations to volume for males, the associations to volume for females, the associations to fat fractions for males, and the associations to fat fractions for females. Color mapping is used to visualize the quantified associations where significant (*p*-value $<2.8\text{x}10^{-3}$). For visualization purposes clipped beta values (from the lower 1% to the highest 1%) were for each experiment linearly rescaled between -2 to +2 maintaining the sign of the association. Non-significant regions are not colored; but instead, a mix of the water and fat magnetic resonance images for the chosen male and female template subjects are shown to allow orientation in the body regions. The first row of images represents coronal slices of the maps, while the remaining rows represent axial slices of those maps in levels of locations of interest (heart, liver, kidneys, abdomen, hip, and thighs). The mid-sagittal slices on the right-hand side of the plots illustrate the visualized slices. The plots on the right-hand side of the collage figures represent the relative amount, from neck to knee, of significant positive and negative associations in the 3D data. The associations are reported separately from lean tissues (where the water signal is stronger than the fat signal) and from adipose tissue (where the fat signal is stronger than the water signal). Full 3D associations are visualized in **Movie S12** (axial and coronal planes).

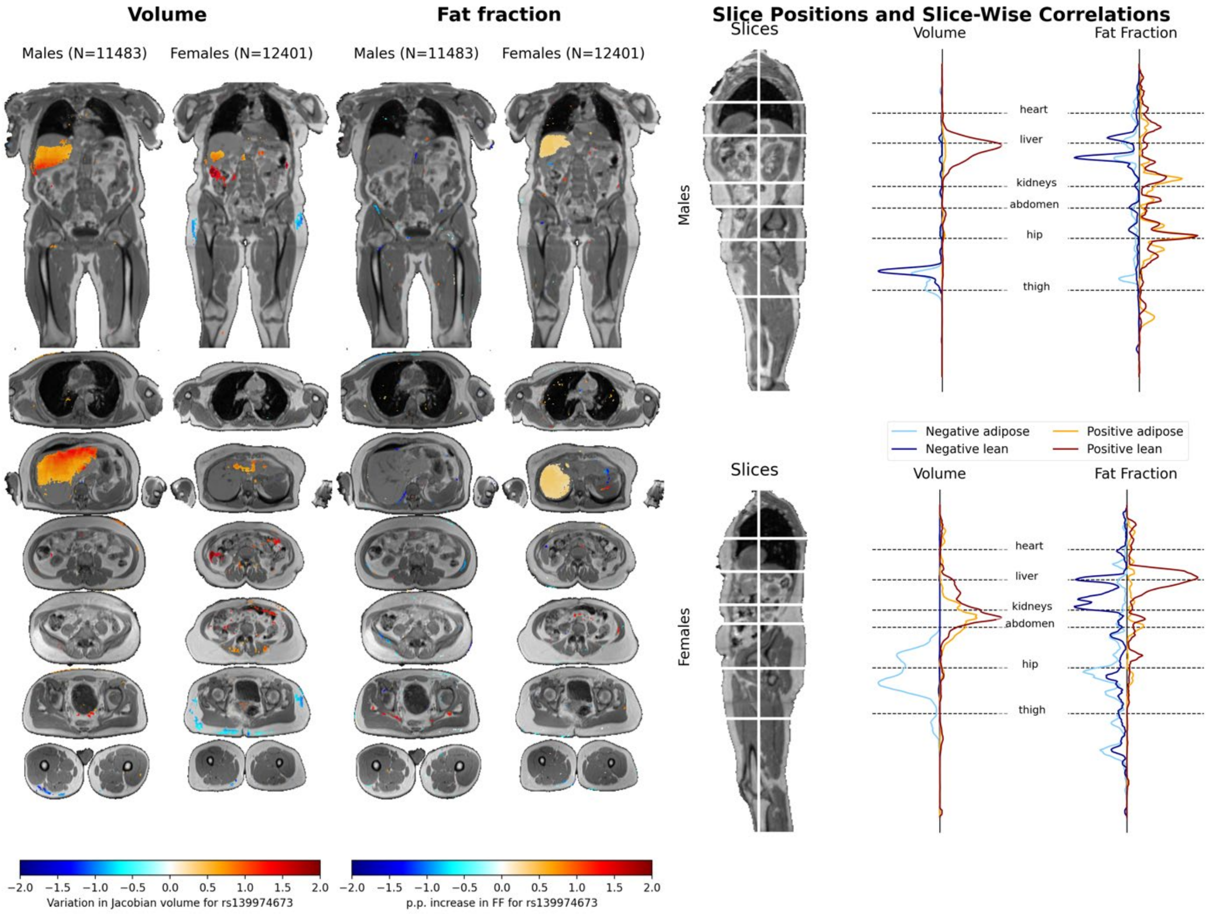

Fig. S19. Imiomics maps for the *PDIA3* rs139974673 variant that quantifies its association to tissue volume and fat fraction voxel-wise throughout the neck-to-knee region. The resulting images show, from left to right, the associations to volume for males, the associations to volume for females, the associations to fat fractions for males, and the associations to fat fractions for females. Color mapping is used to visualize the quantified associations where significant (p-value $<2.8\text{x}10^{-3}$). For visualization purposes clipped beta values (from the lower 1% to the highest 1%) were for each experiment linearly rescaled between -2 to +2 maintaining the sign of the association. Non-significant regions are not colored; but instead, a mix of the water and fat magnetic resonance images for the chosen male and female template subjects are shown to allow orientation in the body regions. The first row of images represents coronal slices of the maps, while the remaining rows represent axial slices of those maps in levels of locations of interest (heart, liver, kidneys, abdomen, hip, and thighs). The mid-sagittal slices on the right-hand side of the plots illustrate the visualized slices. The plots on the right-hand side of the collage figures represent the relative amount, from neck to knee, of significant positive and negative associations in the 3D data. The associations are reported separately from lean tissues (where the water signal is stronger than the fat signal) and from adipose tissue (where the fat signal is stronger than the water signal). Full 3D associations are visualized in **Movie S13** (axial and coronal planes).

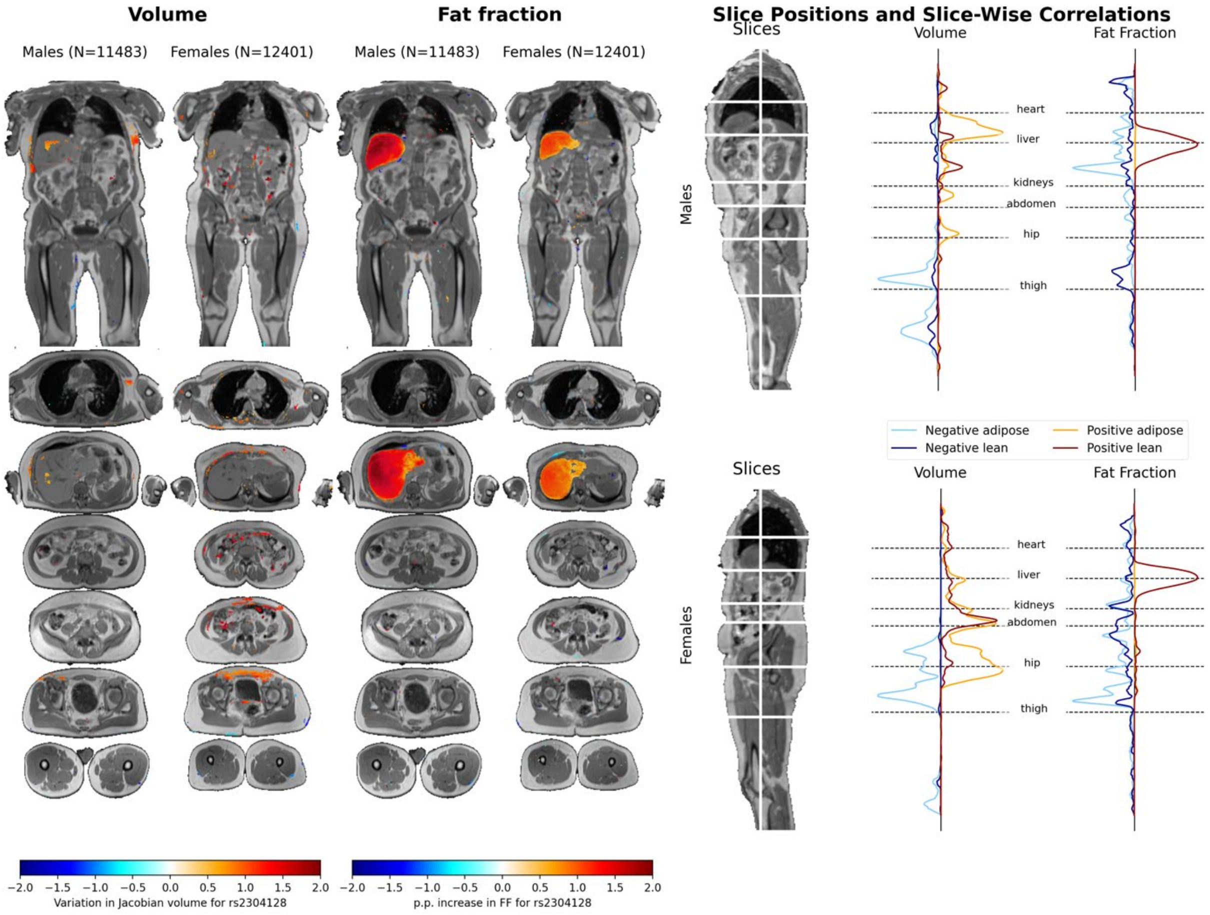

Fig. S20. Imiomics maps for the *LPAR2* rs2304128 variant that quantifies its association to tissue volume and fat fraction voxel-wise throughout the neck-to-knee region. The resulting images show, from left to right, the associations to volume for males, the associations to volume for females, the associations to fat fractions for males, and the associations to fat fractions for females. Color mapping is used to visualize the quantified associations where significant (*p*-value $<2.8\text{x}10^{-3}$). For visualization purposes clipped beta values (from the lower 1% to the highest 1%) were for each experiment linearly rescaled between -2 to +2 maintaining the sign of the association. Non-significant regions are not colored; but instead, a mix of the water and fat magnetic resonance images for the chosen male and female template subjects are shown to allow orientation in the body regions. The first row of images represents coronal slices of the maps, while the remaining rows represent axial slices of those maps in levels of locations of interest (heart, liver, kidneys, abdomen, hip, and thighs). The mid-sagittal slices on the right-hand side of the plots illustrate the visualized slices. The plots on the right-hand side of the collage figures represent the relative amount, from neck to knee, of significant positive and negative associations in the 3D data. The associations are reported separately from lean tissues (where the water signal is stronger than the fat signal) and from adipose tissue (where the fat signal is stronger than the water signal). Full 3D associations are visualized in **Movie S14** (axial and coronal planes).

Supplementary tables (provided in separate excel file)

Table S1. Variables used in the UK Biobank with their respective data-fields.

Table S2. Fine mapping and gene prioritization of liver fat and liver volume genetics variants.

Table S3. Cross-trait associations of liver fat and liver volume genetic variants and their replication in a previous UK Biobank GWAS.

Table S4. Liver fat- and liver volume-associated genetic variants in relation to visceral adipose tissue (VAT) and abdominal subcutaneous adipose tissue (SAT).

Table S5. Genome-wide association analysis of liver volume unadjusted for height (n=24,752).

Supplementary movies (provided in separate files)

Movies S1-S4: 3D visualization of associations in the axial plane and coronal plane for each liver fat genetic variant.

Movies S5-S14: 3D visualization of associations in the axial plane and coronal plane for each liver volume genetic variant.

Supplementary references

[1] Fry D, Almond R, Moffat S, et al. UK Biobank Biomarker Project Companion Document to Accompany Serum Biomarker Data. http://biobankndphoxacuk/showcase/showcase/docs/serum_biochemistrypdf 2019.

[2] Peila R, Rohan TE. Diabetes, Glycated Hemoglobin, and Risk of Cancer in the UK Biobank Study. Cancer Epidemiol Biomarkers Prev 2020.

[3] Censin JC, Peters SAE, Bovijn J, et al. Causal relationships between obesity and the leading causes of death in women and men. PLoS Genet 2019;15:e1008405.

[4] Strand R, Malmberg F, Johansson L, et al. A concept for holistic whole body MRI data analysis, Imiomics. PLoS One 2017;12:e0169966.

[5] Ekstrom S, Malmberg F, Ahlstrom H, et al. Fast graph-cut based optimization for practical dense deformable registration of volume images. Comput Med Imaging Graph 2020;84:101745.

[6] Clarke TK, Adams MJ, Davies G, et al. Genome-wide association study of alcohol consumption and genetic overlap with other health-related traits in UK Biobank (N=112 117). Mol Psychiatry 2017;22:1376-1384.

[7] Howe LJ, Lawson DJ, Davies NM, et al. Genetic evidence for assortative mating on alcohol consumption in the UK Biobank. Nat Commun 2019;10:5039.

[8] Kurki MI, Karjalainen J, Palta P, et al. FinnGen provides genetic insights from a well-phenotyped isolated population. Nature 2023;613:508-518.

[9] Bulik-Sullivan BK, Loh PR, Finucane HK, et al. LD Score regression distinguishes confounding from polygenicity in genome-wide association studies. Nat Genet 2015;47:291-295.

[10] Feizi A, Ray K. otargen: GraphQL-based R package for tidy data accessing and processing from Open Targets Genetics. Bioinformatics 2023;39.

[11] Gazal S, Weissbrod O, Hormozdiari F, et al. Combining SNP-to-gene linking strategies to identify disease genes and assess disease omnigenicity. Nat Genet 2022;54:827-836.

[12] Yang Z, Wang C, Liu L, et al. CARMA is a new Bayesian model for fine-mapping in genome-wide association meta-analyses. Nat Genet 2023;55:1057-1065.
