# Supplementary material for: Impact of genetic variants linked to liver fat and liver volume on MRI-mapped body composition
Source: JHEP Rep. 2025 Jun 2;7(9):101468. doi: 10.1016/j.jhepr.2025.101468 (PMC12355076; doi:10.1016/j.jhepr.2025.101468)
Supplement: Multimedia component 2 [file mmc2.docx]

**JHEP Reports**

**CTAT methods**

Tables for a “Complete, Transparent, Accurate and Timely account” (CTAT) are now mandatory for all revised submissions. The aim is to enhance the reproducibility of methods.

- Only include the parts relevant to your study
- Refer to the CTAT in the main text as ‘Supplementary CTAT Table’
- Do not add subheadings
- Add as many rows as needed to include all information
- Only include one item per row

**If the CTAT form is not relevant to your study, please outline the reasons why:**

|  |
| --- |

- 1. **Antibodies**

| **Name** | **Citation** | **Supplier** | **Cat no.** | **Clone no.** |
| --- | --- | --- | --- | --- |
|  |  |  |  |  |

- 1. **Cell lines**

| **Name** | **Citation** | **Supplier** | **Cat no.** | **Passage no.** | **Authentication test method** |
| --- | --- | --- | --- | --- | --- |
|  |  |  |  |  |  |

- 1. **Organisms**

| **Name** | **Citation** | **Supplier** | **Strain** | **Sex** | **Age** | **Overall n number** |
| --- | --- | --- | --- | --- | --- | --- |
|  |  |  |  |  |  |  |

- 1. **Sequence based reagents**

| **Name** | **Sequence** | **Supplier** |
| --- | --- | --- |
|  |  |  |

- 1. **Biological samples**

| **Description** | **Source** | **Identifier** |
| --- | --- | --- |
|  |  |  |

- 1. **Deposited data**

| **Name of repository** | **Identifier** | **Link** |
| --- | --- | --- |
| GWAS catalog | GCST90566817 (liver fat), GCST90566818 (liver volume, non-height adjusted), and GCST90566819 (liver volume, height adjusted) | https://www.ebi.ac.uk/gwas/search?query=GCST90566817  https://www.ebi.ac.uk/gwas/search?query=GCST90566818  https://www.ebi.ac.uk/gwas/search?query=GCST90566819 |

- 1. **Software**

| **Software name** | **Manufacturer** | **Version** |
| --- | --- | --- |
| Imiomics | Uppsala University | 11-Jan-2022 |

- 1. **Other (*e.g*. drugs, proteins, vectors etc.)**

|  |  |  |
| --- | --- | --- |
|  |  |  |

- 1. **Please provide the details of the corresponding methods author for the manuscript:**

| Main text methods section:  “Magnetic resonance imaging voxel-based technique ("Imiomics")”  Joel Kullberg, Andrés Martínez Mora  Supplementary text section: “Imiomics analysis”  Joel Kullberg, Andrés Martínez Mora |
| --- |

**2.0 Please confirm for randomised controlled trials all versions of the clinical protocol are included in the submission. These will be published online as supplementary information.**

|  |
| --- |
