# Supplementary material for: Impact of genetic variants linked to liver fat and liver volume on MRI-mapped body composition
Source: JHEP Rep. 2025 Jun 2;7(9):101468. doi: 10.1016/j.jhepr.2025.101468 (PMC12355076; doi:10.1016/j.jhepr.2025.101468)
Supplement: Multimedia component 33 [file mmc33.pdf]

Impact of genetic variants linked to liver fat and liver volume on MRI-mapped body composition

Authors

Shafqat Ahmad, Germán D. Carrasquilla, Taro Langner, …, Joel Kullberg, Håkan Ahlström, Tove Fall

Correspondence

german.carrasquilla@sund.ku.dk (G.D. Carrasquilla), tove.fall@medsci.uu.se (T. Fall).

Graphical abstract

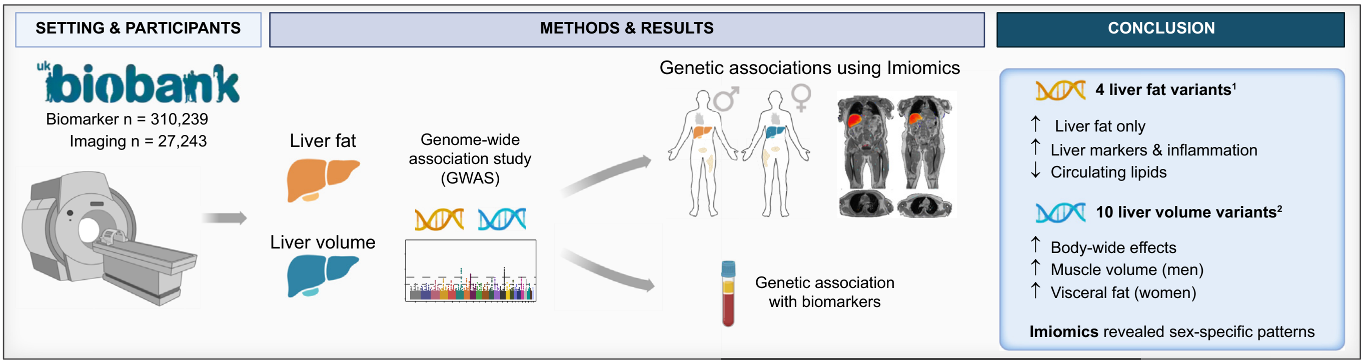

Highlights:

- MRI-based GWAS identified four liver fat and 10 liver volume-associated variants.
- Liver fat variants had localized effects on the liver, with limited effects in other organs, as shown by Imiomics voxel-wise mapping.
- Imiomics analysis revealed heterogeneous and sex-specific effects of liver volume variants across organs and tissues.
- We found evidence suggestive of a novel liver volume locus, *ADH4.*
- Liver fat variants were linked to increased markers of inflammation, liver injury, and lower lipid levels.

Impact and implications:

Liver fat and liver volume are common metabolic traits with a strong genetic component, yet the extent to which they exert organ-specific *vs.* systemic effects remains poorly defined. By integrating genome-wide association analyses and high-resolution neck-to-knee magnetic resonance imaging data through the Imiomics framework, this study reveals distinct genetic architectures for liver fat and liver volume, including sex-specific effects. These findings provide new insights into the biological, organ-level, tissue-specific, and systemic characteristics of steatotic liver disease and its genetic determinants. The results may inform the development of precision imaging genetic approaches, biomarker discovery, and stratified risk assessment strategies, while reinforcing the importance of incorporating sex-specific analyses in future research and clinical applications.

https://doi.org/10.1016/j.jhepr.2025.101468
© 2025 The Authors. Published by Elsevier B.V. on behalf of European Association for the Study of the Liver (EASL). This is an open access article under the CC BY license (http://creativecommons.org/licenses/by/4.0/).

JHEP|Reports

Impact of genetic variants linked to liver fat and liver volume on MRI-mapped body composition

Shafqat Ahmad[1,2,3,†], **Germán D. Carrasquilla**[4,5,*,†], Taro Langner[6,7], Uwe Menzel[1], Nouman Ahmad[6], Sergi Sayols-Baixeras[1,8,9], Koen F. Dekkers[1], Beatrice Kennedy[1], Filip Malmberg[6,10], Ulf Hammar[1], María J. Romero-Lado[4], Jenny C. Censin[11,12], Diem Nguyen[1], Andrés Martínez Mora[6], Tuomas O. Kilpeläinen[4,5], Lars Lind[13], Jan W. Eriksson[14], Robin Strand[6,10], Joel Kullberg[6,7], Håkan Ahlström[6,7], Tove Fall[1,*]

JHEP Reports **2025**. vol. 7 | 1–11

Check for updates

Background & Aims: A quarter of the world population is estimated to have metabolic dysfunction-associated steatotic liver disease. Here, we aim to understand the impact of liver trait-associated genetic variants on fat content and tissue volume across organs and body compartments and on a large set of biomarkers.

Methods: Genome-wide association analyses were performed on liver fat and liver volume estimated with magnetic resonance imaging in up to 27,243 unrelated European participants from the UK Biobank. Identified variants were assessed for associations with fat fraction and tissue volume in >2 million 'Imiomics' image elements in 22,261 individuals and with circulating biomarkers in 310,224 individuals.

Results: We confirmed four liver fat and nine liver volume previously reported genetic variants (p values $<5 \times 10^{-8}$). We further found evidence suggestive of a novel liver volume locus, *ADH4*, where each additional T allele increased liver volume by 0.05 SD (SE = 0.01, p value = 3.3×10^{-8}). The Imiomics analyses showed that liver fat-increasing variants were specifically associated with fat fraction of the liver tissue (p values $<2.8 \times 10^{-3}$) and with higher inflammation, liver and renal injury biomarkers, and lower lipid levels. Associations of liver volume variants with fat content, tissue volume, and biomarkers were more heterogeneous, for example the liver volume-increasing alleles at *CENPW* and *PPP1R3B* were associated with higher skeletal muscle volumes and were more pronounced in men, whereas the *GCKR* variant was negatively associated with lower skeletal muscle volumes in women (p values $<2.8 \times 10^{-3}$).

Conclusions: Liver fat-increasing variants were mostly linked to fat fraction of the liver and were positively associated with some adverse metabolic biomarkers and negatively with lipids. In contrast, liver volume-associated variants showed a less consistent pattern across organs and biomarkers.

© 2025 The Authors. Published by Elsevier B.V. on behalf of European Association for the Study of the Liver (EASL). This is an open access article under the CC BY license (http://creativecommons.org/licenses/by/4.0/).

Introduction

Metabolic dysfunction-associated steatotic liver disease (MASLD) is the most common chronic liver disease, with its global prevalence estimated to have increased from 25% to 38% over the past three decades – a trend that is expected to continue.[1] MASLD is characterized by abnormal liver fat accumulation and can lead to severe liver conditions such as cirrhosis, liver failure, and hepatocellular carcinoma.[2,3] It is also an independent risk factor for extrahepatic complications such as cardiovascular disease, type 2 diabetes, and chronic kidney disease.[4] Magnetic resonance imaging (MRI) is considered the non-invasive gold standard method for liver fat quantification given its accuracy and reproducibility, surpassing traditional biopsy methods in safety and reliability.[5,6] MRI provides precise and reproducible quantitative volumetric assessments of hepatic steatosis[5] and can be used to measure liver volume. Increased liver volume has been observed in the early stages of MASLD and is often reduced in cirrhosis.[7,8]

Understanding the genetic architecture of MASLD phenotypes can enhance insights into pathophysiology and aid in discovering new molecular targets. Twin and family studies have estimated the heritability of MASLD to be 22–70%.[9] Although genetic studies on MASLD and hepatic function biomarkers are expanding,[10–12] most research is based on the UK Biobank, and the largest analysis for MRI-derived liver phenotypes includes approximately 37,000 participants.[3,13–16] Studies of the impact of liver fat- and liver volume-independent variants across hepatic and extrahepatic neck-to-knee regions are still lacking.

Epidemiological evidence shows clear sex differences in ectopic fat distribution,[17] but most genetic studies combine the analysis for both sexes, overlooking such differences. To address these gaps, our study employed neck-to-knee 3D

* Corresponding authors. Addresses: Molecular Epidemiology, Department of Medical Sciences, Uppsala University, Uppsala 75185, Sweden (T. Fall); Novo Nordisk Foundation Center for Basic Metabolic Research, Faculty of Health and Medical Sciences, University of Copenhagen, Copenhagen 2200, Denmark (G.D. Carrasquilla).
E-mail addresses: tove.fall@medsci.uu.se (T. Fall), german.carrasquilla@sund.ku.dk (G.D. Carrasquilla).
† Shared first authorship.
https://doi.org/10.1016/j.jhepr.2025.101468

voxel-based maps ('Imiomics') to provide spatially detailed information about organ morphology, fat content, and tissue volume across different sex-specific body maps based on MRI data using the UK Biobank.[18] Thus, we aimed to investigate the associations between liver fat- and liver volume-associated genetic variants and detailed measurements of fat content and tissue volume across various body compartments and organs. Additionally, we explored how these genetic variants were associated with clinical and cardiometabolic biomarkers.

Materials and methods

Ethics statement

This research was conducted using the UK Biobank resource under application number 14237 and written informed consent from all participants was provided before the study. The UK Biobank has ethical approval from the Northwest Multi-Centre Research Ethics Committee (ref: 11/NW/0382) and the current study was further approved by the Swedish Ethical Review Authority (DNR 2019-03073).

Study sample and measurements

The UK Biobank is a prospective epidemiological cohort with deep phenotypic and genetic data collected for 502,655 individuals aged 40–69 years at recruitment 2006–2010 across 22 research centers in the UK.[19,20] At the baseline assessment, participants answered questions about lifestyle and sociodemographic parameters, provided biological samples, and underwent clinical measurements. Details on the baseline assessment and biochemical assays have been published previously.[19–21] A summary of the phenotypes relevant to this study, including anthropometric and biomarkers measures, alcohol intake, and clinical diagnosis of MASLD and chronic liver disease (CLD), is found in the Supplementary Materials and methods and Table S1.

Assessment of liver fat

Starting in 2014, a randomly selected subset of UK Biobank participants, up to 100,000 participants, are continuously invited to a neck-to-knee MRI. For the current analysis, imaging data was extracted for 38,949 participants in late 2019.[22] Neck-to-knee body MRI scans (Data-Field 20201) were acquired using a Siemens 1.5T MAGNETOM Aera (Siemens Healthcare, Erlangen, Bavaria, Germany) with a dual-echo Dixon technique. Liver fat content was estimated using the proton density fat fraction (PDFF), which calculates liver fat as the proportion of fat to total tissue signal (fat + water). At the time of analysis, direct PDFF measurements were available for 9,893 participants.[23] To extend liver fat estimation to a larger group, a neural network trained by Langner *et al.*[24] was applied to the MRI images. This deep learning model was trained using reference liver fat measurements and validated through a 10-fold cross-validation process. After validation, the model was applied to infer liver fat content in the remaining imaging data cohort. This resulted in a high-quality dataset of 38,949 participants with measured or inferred liver fat. This method achieved a strong regression fit in cross-validation (R^2 = 0.94).

Assessment of liver volume

We quantified liver volume using image segmentation of the water MRI images from the abdominal scans. The liver segmentation model was trained on a dataset of 97 manually segmented livers, and subsequently applied to segment the livers of all participants. The model used was a modified 2.5D U-Net[25] with a ResNet encoder pre-trained on ImageNet.[26] It was trained and applied slice-by-slice on axial slices and in addition, included the closest neighboring slices as input data. From the liver segmentation method, the liver volume was derived by multiplying the number of voxels segmented with the known volume of each voxel. The segmentation approach was evaluated against manual reference segmentations, demonstrating the following performance: Dice coefficient: 0.95 ± 0.02, R^2 = 0.86 and mean absolute error 77 ml.[27] To ensure data quality, procedures were implemented to address potential segmentation challenges, including anatomical variations such as liver cysts, high liver iron, and prior liver resection. This resulted in a high-quality liver volume dataset of 36,957 participants.

Genotyping and genome-wide association study

Genotyping, quality control, and imputation procedures in the UK Biobank cohort have previously been described in detail[19] and are summarized in the Supplementary Materials and methods. By only including individuals that are part of in Data-Field 22020 provided by UK Biobank, we removed samples with excess heterozygosity, sex mismatch, high missingness (>0.05 on autosomes), and individuals who were not part of a maximal set of unrelated individuals up to third degree.[19] Furthermore, only participants with European ethnic background were included, based on Data-Field 22006. Lastly, by matching this data set with the liver phenotype data, we achieved a final analytical genome-wide association study (GWAS) dataset of 27,243 for liver fat and 24,752 for liver volume. GWAS was conducted using PLINK v2.0 (www.cog-genomics.org/plink/2.0/). We excluded single nucleotide polymorphisms (SNPs) with call rate <0.9, minor allele count ≤30, Hardy-Weinberg exact test p value $<1 \times 10^{-6}$, and an imputation quality range (IMPUTE2 information metric) <0.8. To meet the assumption of normality and finite variance in the trait distribution, phenotype variables (liver fat and liver volume) were transformed using the age- and sex-adjusted two-stage inverse-normal transformation; the procedure is detailed in the Supplementary Materials and methods. We used a linear regression model adjusting for age at baseline, sex, genotyping arrays, and the first 20 principal components to control for population stratification. Genome-wide significance was set at p value $<5 \times 10^{-8}$, with genomic inflation assessed using quantile-quantile plots and the genomic inflation factor (λ). Body frame may constitute an important confounder in the association of genetic variants and liver volume, and as such the liver volume GWAS analysis was further adjusted for baseline height measures. Regional association plots of liver fat- and liver volume-associated genetic variants were visualized using LocusZoom.[28] Chromosomal position is based on the GRCh37/hg19 build.

Genetic correlations, pruning and conditional analysis, and gene prioritization

We used linkage disequilibrium (LD) score regression to assess genetic correlations[29] using GWAS data on hepatic diseases

from FinnGen.[30] To identify independent SNPs, we used two procedures: (a) conditional joint (COJO) analysis[31] and (b) LD clumping. Results from both approaches were comparable, and the COJO results are presented herein. Results from the original GWAS are available online (see Data availability statement). We retrieved GWAS summary statistics from a recent study on liver fat and liver volume in the UK Biobank for comparison.[13] To assess the association with visceral adipose tissue (VAT) in a larger sample (n = 38,965), we cross-referenced with a VAT GWAS[32] from the UK Biobank. Fine mapping of the signals was performed using CARMA,[33] a Bayesian method that integrates functional annotations and accounts for LD discrepancies to refine causal variant identification, prioritizing variants with high posterior inclusion probabilities and was followed by gene prioritization using a combined SNP-to-gene strategy (cS2G).[34] We also applied an alternative variant-to-gene (V2G) approach based on Open Target Genetics, using the otargen R package (R Foundation for Statistical Computing, Vienna, Austria).[35] Details have been provided in the Supplementary Materials and methods.

MRI voxel-based technique ('Imiomics')

Imiomics is a framework to visualize the association of non-image-based phenotypes with over 2 million 3D image elements based on neck-to-knee MRI. In Imiomics analysis, image registration is a crucial component where a target image is deformed to align with a sex-specific reference image by applying a deformation field. Each point in the reference image corresponds to a specific image point in the target image, and these point-to-point correspondences form the basis of the Imiomics analysis.[18,36] We evaluated Imiomics maps for unrelated study participants of European ancestry with available MRI data, using the same selection as for study participants for GWAS analysis. For each individual, a 'volume image' with voxel-wise (>2 million 3D image elements) information about the magnitude of the deformation in each region was obtained, together with a 'fat fraction image' consisting of the original fat fraction image of each individual deformed into the template subject.

We assessed the association of each liver trait-associated SNP with fat fraction and tissue volume in each of these image elements using linear regression adjusted for age, age squared, total body fat, height, and 20 principal components stratified by sex in a sample of 11,483 men and 12,401 women. We display the results in Imiomics neck-to-knee association maps for the liver trait-increasing allele. We corrected *p* values with the Bonferroni method for 18 pre-defined body areas. Details of the Imiomics analysis are included in the Supplementary Materials and methods.

Association of liver genetic variants with biomarkers, alcohol intake, and liver disease

Linear regression analyses were conducted to evaluate the potential impact of liver-associated genetic variants on 30 cardiometabolic biomarkers that were ln-transformed and alcohol intake, and are reported in the Supplementary Materials and methods and Table 1. These phenotypes were collected at the baseline visit (Table S1). These analyses were conducted in the biomarker subset consisting of 310,224 unrelated individuals of European ancestry from the UK Biobank who were not part of the liver GWAS analysis. The models were adjusted for age, sex, genotyping array, and the first 20 principal components. For imputed genotypes, dosages were converted to the closest integer. Further, we evaluated the association between genetic variants with the diagnosis of MASLD and CLD where these diseases were diagnosed using International Classification of Diseases-coded health records (details in the Supplementary Materials and methods). For this, we applied logistic regression analysis adjusting for the same covariates as in the linear models.

Results

Baseline characteristics and cardiometabolic biomarkers for the MRI subset (n = 27,243) and those in the biomarker subset (n = 310,224) are reported in Table 1, including measurement units. The mean (SD) age for individuals at baseline in the GWAS was 55.7 (7.4) years and the age at imaging was 64.6 (7.5) years. The mean liver fat content in the MRI subset was 3.9% (SD: 4.3), and the mean liver volume was 1,419 cm^3 (SD: 300).

GWAS analysis identified genetic variants of liver fat and liver volume

Four independent genetic variants were associated with liver fat (λ = 1.05) and 10 independent genetic variants were associated with liver volume (λ = 1.06) (Table 2, Fig. S1–S3). For liver fat, variants were located near the genes *PNPLA3*, *TM6SF2*, and *APOE*, all of which were previously reported as associated with liver fat in the UK Biobank (Table S2).[13] Fine mapping using CARMA, which integrates functional annotations and accounts for LD structure to enhance causal variant identification, and cS2G suggested *MAU2, CILP2, APOE*, and *PNPLA3* as candidate genes, whereas the V2G approach suggested *MAU2, SUGP1, APOE*, and *SAMM50* (Table S2). The liver volume GWAS confirmed nine previously reported associated genetic variants, and suggested a novel associated intronic variant in *ADH4* (Tables 2 and S3 and Fig. S1 and S3).[13] Gene prioritization was concordant between the two prioritization approaches for *GCKR* and *CENPW* (Table S2). We found one liver volume variant *LPAR* rs2304128 to be in moderate LD (R^2 = 0.69) with the liver fat variant *TM6SF2* rs8107974. For both, *MAU2* was suggested as the causal gene by the V2G approach (Table S2).

Additionally, genetic correlation analysis showed positive genetic correlations between our liver traits and MASLD, metabolic dysfunction-associated steatohepatitis (MASH), but negative correlations between liver fat and liver volume as illustrated in Fig. S4. Cross-referencing our variants with a previous GWAS[32] on VAT and subcutaneous adipose tissue (SAT) in the abdominal region showed nominal associations of the *APOE* rs429358 liver fat-associated variant with increased VAT and SAT in the abdominal region. Moreover, the *TM6SF2* rs188247550 liver fat-associated variant was nominally associated with abdominal SAT and the *REEP3* rs7896518 liver volume-associated variant with VAT as detailed in Table S4.

Association of liver variants with body composition using Imiomics maps

Associations of liver fat and liver volume genetic variants are summarized for 18 pre-defined body areas in Fig. S5 and S6,

Table 1. Characteristics of the individuals from the UK Biobank at baseline who were in the MRI subset and in the biomarker subset.

	MRI subset (n = 27,243, 51% women)			Biomarker subset (n = 310,224, 54% women)		
Description	N	Mean	SD	N	Mean	SD
Age at baseline, years	27,242	55.7	7.4	310,224	57.6	8.0
Body mass index, kg/m^2	27,241	26.6	4.2	309,185	27.5	4.8
Waist-to-hip ratio, cm/cm	27,241	0.9	0.1	309,614	0.9	0.1
Standing height, cm	27,241	169.8	9.2	309,529	168.7	9.2
Alkaline phosphatase, U/L	25,895	79.8	23.5	295,858	83.9	26.7
Alanine aminotransferase, U/L	25,892	23.0	13.8	295,722	23.6	14.0
Aspartate aminotransferase, U/L	25,797	25.7	9.9	294,750	26.2	10.7
Gamma-glutamyltransferase, U/L	25,885	33.7	34.6	295,694	37.7	42.3
Direct bilirubin, µmol/L	22,458	1.8	0.8	251,276	1.8	0.9
Total bilirubin, µmol/L	25,775	9.4	4.6	294,617	9.1	4.4
Apolipoprotein A, g/L	23,522	1.5	0.3	269,380	1.5	0.3
Apolipoprotein B, g/L	25,775	1.0	0.2	294,406	1.0	0.2
Lipoprotein A, nmol/L	20,738	44.1	49.3	235,076	44.2	49.5
C-reactive protein, mg/L	25,839	2.0	3.5	295,203	2.6	4.4
Glucose, mmol/L	23,619	5.0	1.0	270,718	5.1	1.2
Glycated hemoglobin, mmol/mol	25,829	35.0	5.1	295,772	36.0	6.6
HDL cholesterol, mmol/L	23,638	1.5	0.4	270,884	1.5	0.4
LDL cholesterol, mmol/L	25,839	3.6	0.8	295,293	3.6	0.9
Triglycerides, mmol/L	25,870	1.7	1.0	295,605	1.8	1.0
Cholesterol, mmol/L	25,895	5.7	1.1	295,844	5.7	1.1
Urea, mmol/L	25,870	5.3	1.2	295,658	5.4	1.4
Calcium, mmol/L	23,647	2.4	0.1	270,893	2.4	0.1
Albumin, g/L	23,647	45.4	2.5	271,001	45.2	2.6
Creatinine, µmol/L	25,874	72.5	14.0	295,700	72.4	18.1
Phosphate, mmol/L	23,602	1.2	0.2	270,485	1.2	0.2
Total protein, g/L	23,614	72.2	3.9	270,708	72.4	4.0
Urate, µmol/L	25,865	304.0	77.4	295,460	310.0	80.5
Cystatin C, mg/L	25,893	0.9	0.1	295,825	0.9	0.2
Insulin-like growth factor-1, nmol/L	25,766	22.1	5.5	294,247	21.3	5.7
Sex hormone-binding globulin, nmol/L	23,418	52.0	27.8	268,397	51.8	27.6
Number of overweight (%)	16,630 (61)			209,361 (67.5)		
Number of obese (%)	4,844 (17.8)			77,350 (24.9)		

Values are reported as mean and SD. Overweight is defined as individuals having a body mass index (BMI) ≥25.0 kg/m^2, and obese are individuals having a BMI ≥30.0 kg/m^2. MRI, magnetic resonance imaging; N, sample size; NA, not applicable; SD, standard deviation.

Table 2. Independent genome-wide significant genetic variants for liver fat and liver volume and their prioritized genes. Although rs188247550 and rs7896518 are classified as intronic variants by OTG, they are not located within the genes closest to their transcription start sites (*TM6SF2* and *REEP3*, respectively) but within *SUGP1* and *JMJD1C*

SNP	Chr	Position*	Nearest gene	EA	OA	EAF	Beta	SE	*p* value	Variant type	cS2G	OTG-V2G
**Liver fat variants**												
rs8107974	19	19388500	*TM6SF2*	T	A	0.08	0.29	0.02	7.3×10^{-73}	Intronic	*MAU2*	*MAU2*
rs188247550	19	19396616	*TM6SF2*	T	C	0.01	0.27	0.04	6.2×10^{-14}	Intronic	*CILP2*	*SUGP1*
rs429358	19	45411941	*APOE*	T	C	0.85	0.09	0.01	6.1×10^{-15}	Missense	*APOE*	*APOE*
rs738408	22	44324730	*PNPLA3*	T	C	0.21	0.19	0.01	2.5×10^{-78}	Synonymous	*PNPLA3*	*SAMM50*
**Liver volume variants**												
rs193084249	1	26987646	*ARID1A*	G	A	0.02	0.17	0.03	4.5×10^{-09}	Intergenic	*ZDHHC18*	*ARID1A*
rs1260326	2	27730940	*GCKR*	T	C	0.39	0.08	0.01	4.3×10^{-20}	Missense	*GCKR*	*GCKR*
rs79287178	3	172294500	*TNFSF10*	A	G	0.03	0.18	0.03	3.0×10^{-11}	Intronic	NA	*TNFSF10*
rs6858148	4	100065917	*ADH4*	T	C	0.65	0.05	0.01	3.3×10^{-08}	Intronic	*ADH4, ADH5*	*ADH5*
rs853966	6	127037236	*CENPW*	T	G	0.54	0.06	0.01	9.5×10^{-11}	Regulatory region	*CENPW*	*CENPW*
rs4240624	8	9184231	*PPP1R3B*	G	A	0.09	0.18	0.02	3.0×10^{-33}	Intronic	*RP11-115J16.1*	*PPP1R3B*
rs10881959	10	93507964	*TNKS2*	T	G	0.43	0.05	0.01	2.6×10^{-09}	Intergenic	NA	*REEP3*
rs7896518	10	65104500	*REEP3*	A	G	0.57	0.07	0.01	2.3×10^{-14}	Intronic	*TNKS2-DT*	*TNKS2*
rs139974673	15	44027885	*PDIA3*	C	T	0.02	0.25	0.03	3.2×10^{-19}	Intronic	*CKMT1A*	*ADAL*
rs2304128	19	19746151	*LPAR2*	T	G	0.08	0.10	0.02	7.1×10^{-10}	Intronic	*GMIP*	*MAU2*

The nearest gene is defined based on the distance to the start site. Gene prioritization results are from combined SNP-to-gene strategy (cS2G) and Open Targets Genetics (OTG) highest variant-to-gene (V2G) score. *Chromosomal position is based on the GRCh37/hg19 build. Linear regression models were used in these analyses, adjusting for age at baseline, sex, genotyping arrays, and the first 20 principal components to control for population stratification. The liver volume GWAS analysis was further adjusted for baseline height measures. Genome-wide significance was set at *p* value $<5\times10^{-8}$.
Chr, chromosome; EA, effect allele; EAF, effect allele frequency; NA, not applicable; OA, other allele; SE, standard error; SNP, single nucleotide polymorphism.

respectively. We applied a significance threshold of *p* value $<2.8 \times 10^{-3}$, accounting for multiple comparisons. Figs. 1 and 2 illustrate the associations of the genetic variants with two-dimensional body maps at pre-defined sections. For each genetic variant, results are presented for the liver trait-increasing effect allele and reported using the closest gene name (Fig.

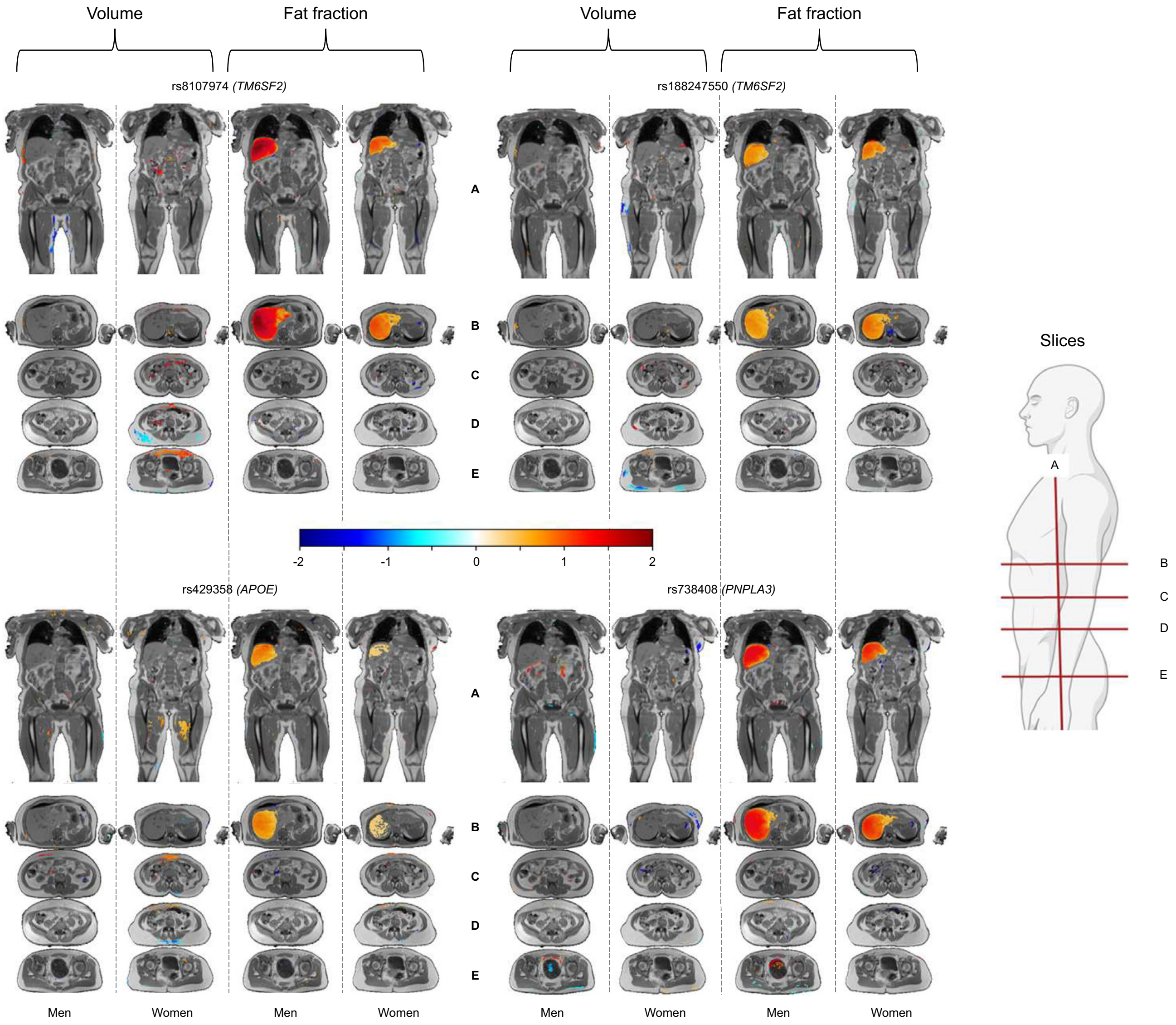

Fig. 1. Neck-to-knee voxel maps of volume and fat fraction for four liver fat variants. The figure shows statistical 3D associations between magnetic resonance imaging (MRI) data and single nucleotide polymorphism (SNP) in men and women. Each panel displays volume maps for men, volume maps for women, fat fraction maps for men, and fat fraction maps for women. Linear regression models were used in these analyses, adjusting for age, age squared, total body fat, height, and 20 principal components stratified by sex. *p* values were corrected with the Bonferroni method for 18 pre-defined body areas. Significant associations (p value $<2.8 \times 10^{-3}$) are color-coded from negative (blue) to positive (red). For visualization purposes clipped beta values (from the lower 1% to the highest 1%) were for each experiment linearly rescaled between -2 and +2 maintaining the sign of the association. Non-significant regions are uncolored and instead show underlying anatomical MRI images for reference. Coronal slices (A) and axial slices at the liver (B), kidneys (C), abdomen (D), and hip (E) are shown. APOE, apolipoprotein E; PNPLA3, patatin like domain 3, 1-acylglycerol-3-phosphate O-acyltransferase; TM6SF2, transmembrane 6 superfamily member 2.

S7–S20). Supplementary Movies S1–S4 and S5–S14 show associations for liver fat- and liver volume-associated genetic variants across the neck-to-knee region, respectively. Associations are shown in both axial and coronal planes.

Supplementary video related to this article can be found at https://doi.org/10.1016/j.jhepr.2025.101468

Liver fat genetic variants

As expected, all four liver fat variants showed positive association with liver fat fraction in the Imiomics analysis in both men and women. We did not observe evidence for the effects of these genetic variants on fat fraction in any other compartments of the body (Figs 1, S5, and S7–S10, Supplementary Movies S1–S4). The liver fat variants showed a few associations with the volume of adipose tissue across parts of the studied body compartments (Figs 1 and S5). We observed signals for the *TM6SF2* rs188247550 and rs8107974 variants with lower body SAT, including lower volume of gluteal and thigh SAT in women (rs188247550), and lower volume of gluteal SAT in women and lower thigh SAT in men (rs8107974). The *TM6SF2* rs8107974 variant further showed regional signals for lower abdominal posterior SAT volume and higher thorax and abdominal SAT volume in women (Fig. S7). The *PNPLA3* variant was negatively regionally associated with thigh SAT in men and positively with higher gluteal SAT in women (Fig. S10). No associations were observed

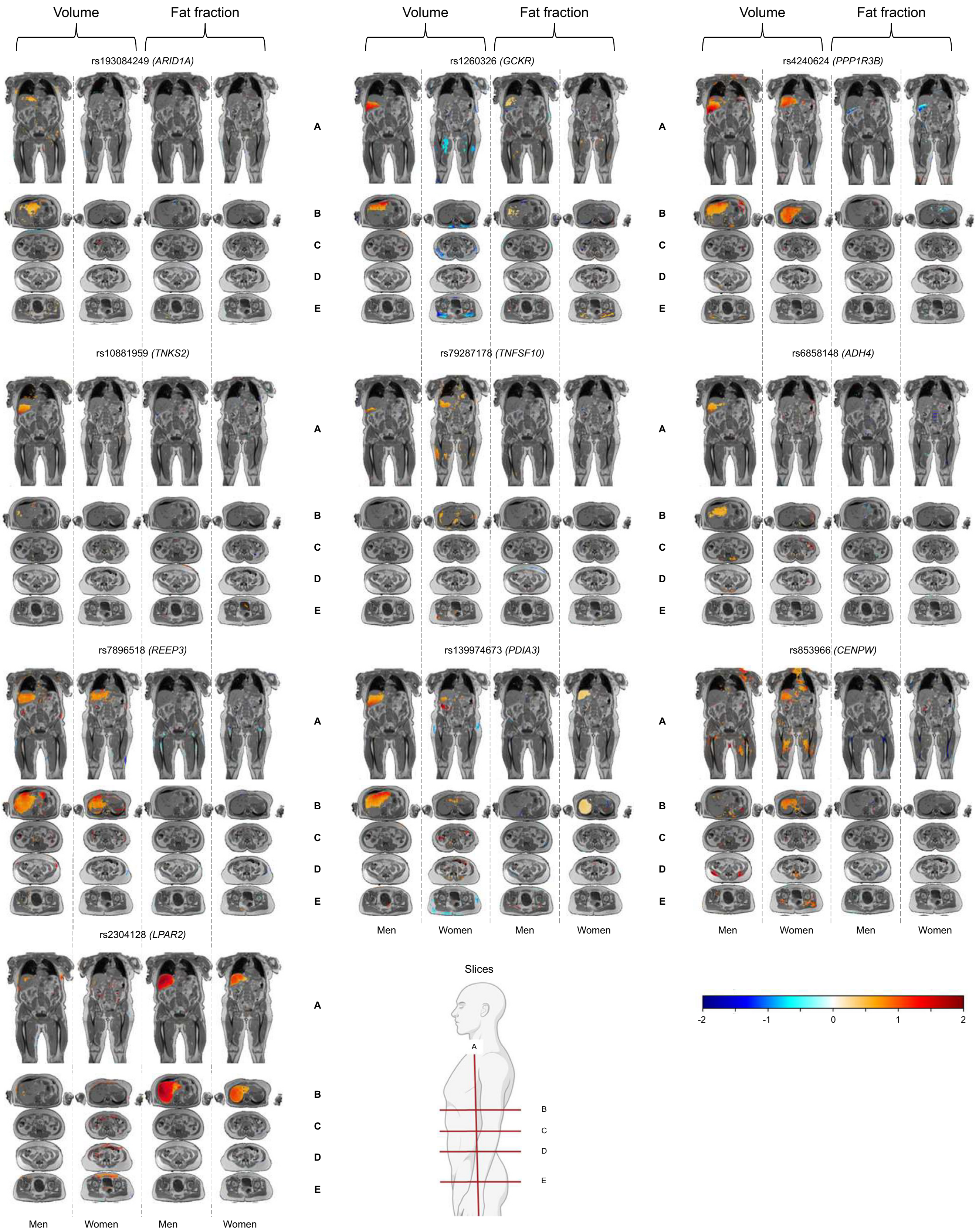

Fig. 2. Neck-to-knee voxel maps of volume and fat fraction for 10 liver volume variants. The figure shows statistical 3D associations between magnetic resonance imaging (MRI) data and single nucleotide polymorphism (SNP) in men and women. Each panel displays volume maps for men, volume maps for women, fat fraction maps for men, and fat fraction maps for women. Linear regression models were used in these analyses, adjusting for age, age squared, total body fat, height, and 20 principal components stratified by sex. *p* values were corrected with the Bonferroni method for 18 pre-defined body areas. Significant associations (*p* value $<2.8 \times 10^{-3}$)

between the liver fat variants and the volume of the lung, heart, liver, and skeleton, except for a positive signal for parts of thigh muscle volume for the *APOE* variant (Fig. S5 and S9).

Liver volume variants

All liver volume variants showed positive associations with liver volume in the Imiomics except for the *LPAR2* variant (Fig. 2). However, the *ARID1A, GCKR, ADH4,* and *TNKS2* variants only showed an association with liver volume in men and *CENPW* only in women in this analysis (Figs 2 and S6). We observed a variety of body-wide effects of the genetic variants, where the liver volume-increasing allele of the *CENPW* variant had regional positive associations with volumes of gluteofemoral muscles in both women and men and thoracic and abdominal muscles in men (Fig. S15). For the *GCKR* variant, the association pattern contrasted with that of the *CENPW* variant, where regional negative associations were noted in women for the *GCKR* variant with skeletal muscle volumes and with lower thigh SAT in men (Fig. S12). The *ADH4* variant was associated to higher VAT in women, and greater abdominal skeletal muscle volume in men (Fig. S14). The *PPP1R3B* variant was associated with increased thoracic and gluteal skeletal muscle volume in men (Fig. S16). The *REEP3* and *PDIA3* variants was associated with greater heart volume in men (Fig. S18 and S19) and the *TNSF10* with larger heart volume in women (Fig. S13). The *PDIA3* variant also showed regional negative associations with gluteal SAT adipose tissue volumes in women (Fig. S19). The *LPAR2* variant was positively associated with thorax SAT in men and abdominal SAT in women (Fig. S20). There were only a few associations of liver volume variants with the fat fraction across tissue compartments (Figs 2 and S6). The *GCKR* variant was regionally positively associated with the fat fraction in the skeletal muscle of the gluteal area in women and with the fat fraction of the liver in men (Fig. S12). The *LPAR2* variant was positively associated with the liver fat fraction in both men and women (Fig. S20) and the *ADH4* variant was associated with the lower disc fat fraction in women (Fig. S14). Finally, the *CENPW* variant was negatively associated with the fat in femur in both men and women and with the fat fraction in spine and pelvic bone in men (Fig. S15).

Genetic variants for liver fat content associated with various biomarkers and liver disease traits

Next, we tested the association of the identified liver fat variants with biomarkers, MASLD, CLD, and alcohol intake. All liver fat-increasing alleles were associated with increased alanine aminotransferase and direct bilirubin and were negatively associated with cholesterol. The *APOE* and *TM6SF2* variants were associated with increased markers of inflammation, glycemia and kidney markers, and increased risk of CLD, but negatively associated with LDL cholesterol, triglycerides, and apolipoprotein B. The *TM6SF2* rs8107974 and *PNPLA3* variants were associated with an increased risk of MASLD. The *PNPLA3* variant had a different pattern with negative associated with CRP, ALP, IGF1, apolipoprotein A, apolipoprotein B, HDL cholesterol, and urate. There were no associations with alcohol intake (Fig. 3).

Genetic variants for liver volume-associated with various biomarkers and liver disease traits

We also analyzed the association of liver volume-increasing variants with biomarkers, MASLD, CLD, and alcohol intake. All variants were negatively associated with sex hormone binding globulin except *LPAR2* and *CENPW*. Variants in the *GCKR, PDIA3,* and *ARID1A* loci showed associations with most biomarkers (Fig. 3).

Discussion

We leveraged MRI-based neck-to-knee 3D voxel-based mapping of liver fat and liver volume genetic markers to uncover metabolic and sex-specific patterns in the UK Biobank cohort. Our GWAS analysis confirmed 13 previously reported associations of genetic variants with liver traits[13,16,37] and suggested a novel association of an intronic variant in *ADH4*, the alcohol dehydrogenase 4 gene, with liver volume. Our study extends previous liver phenotype GWAS analyses by mapping liver trait variants with neck-to-knee fat fraction and tissue volume at a granular spatial resolution using the Imiomics concept. Previous GWAS and candidate gene studies have characterized associations between liver trait-associated variants at *TM6SF2, PNPLA3*, and *GCKR* with MASLD and fat distribution,[16,38] but these primarily focused on large-scale association analyses without spatially resolved assessments. We found liver fat genetic variants to be largely specifically associated with increased fat fraction in the liver. In contrast, liver volume genetic variants displayed more heterogeneous associations with tissue volume. Liver trait-associated variants were associated with a broad range of cardiometabolic biomarkers.

Our results for liver fat-associated variants in or near *TM6SF2*, *APOE*, and *PNPLA3* are in general consistent with previous reports showing that these variants are positively associated with markers of liver and kidney dysfunction and inflammation and negatively associated with cholesterol lipoproteins.[39–42] Our Imiomics analysis showed that the four liver fat genetic variants were exclusively associated with increased fat fraction in the liver. The *PNPLA3* liver fat variant has been associated with lower body weight, BMI, and fat percentage in healthy individuals, particularly in women.[43] Previous research demonstrated that genetically determined higher liver fat is causally associated with an increased risk of liver cirrhosis and hepatic cancer.[3] In our study, we found positive genetic correlations between our liver traits and MASLD and MASH, indicating that these traits share features in their genetic background supporting previous findings.

are color-coded from negative (blue) to positive (red). For visualization purposes clipped beta values (from the lower 1% to the highest 1%) were for each experiment linearly rescaled between -2 and +2 maintaining the sign of the association. Non-significant regions are uncolored and instead show underlying anatomical MRI images for reference. Coronal slices (A) and axial slices at the liver (B), kidneys (C), abdomen (D), and hip (E) are shown. ADH4, alcohol dehydrogenase 4; ARID1A, AT-rich interaction domain 1A; CENPW, centromere protein W; GCKR, glucokinase regulatory protein; LPAR2, lysophosphatidic acid receptor 2; PDIA3, protein disulfide isomerase family A member 3; PPP1R3B, protein phosphatase 1 regulatory subunit 3B; REEP3, receptor accessory protein 3; TNFSF10, tumor necrosis factor ligand superfamily member 10; TNKS2, tankyrase 2.

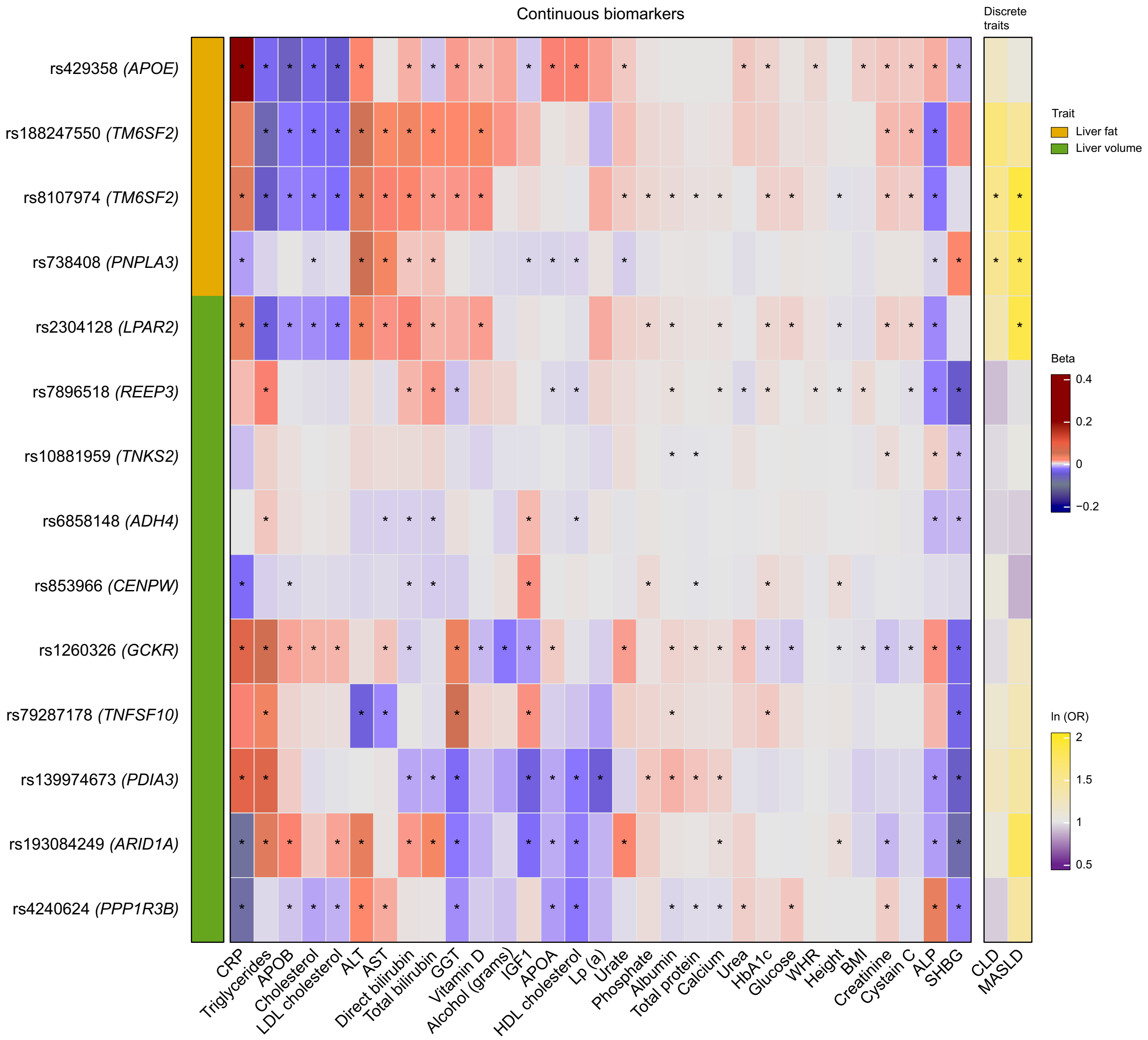

Fig. 3. Associations of liver fat and liver volume variants with biomarkers and liver disease. Each row in the heatmap represents a liver fat or liver volume genetic variant. Liver fat variants are in orange, and liver volume variants are in green in the first column. Each column in the heatmap represents a biomarker or a discrete trait. The biomarkers are clustered with hierarchical clustering. The heatmap is color-coded by strength of association according to color legend. *p value <0.05. Linear regression models were used for analyses of continuous traits and logistic regression models for discrete traits. Models were adjusted for age, sex, genotyping array, and the first 20 principal components. All the biomarker concentrations were log-transformed before the analysis. Discrete traits are shown as natural log of the OR. ALP, alkaline phosphatase; ALT, alanine aminotransferase; AST, aspartate aminotransferase; APOA, apolipoprotein A; APOB, apolipoprotein B; CLD, chronic liver disease; CRP, C-reactive protein; GGT, gamma-glutamyl transferase; HBA1c, hemoglobin A1c; IGF1, insulin-like growth factor 1; Lp(a), lipoprotein A; MASLD, metabolic dysfunction-associated steatotic liver disease; OR, odds ratio; SHBG, sex hormone-binding globulin; WHR, waist-to-hip ratio.

Our novel finding of associations of variants in *ADH4* with liver volume suggests a potential role for alcohol metabolism genes in liver size and systemic fat distribution but warrants replication in external material. The *ADH4* variant was associated with higher VAT volume in women, greater abdominal skeletal muscle volume in men, and decreased fat fraction in discs in women. Liu *et al.*,[13] who have previously assessed variants at the genome-wide scale with liver volume in the UK Biobank did not highlight this variant in a slightly larger and largely overlapping sample (p value = 1 × 10^{-4}, n = 32,860). This could be because of differences in liver volume assessment, model specification, or sample differences. Our results showed a slightly stronger association in the height-adjusted model than in the non-adjusted model (3.3 × 10^{-8} *vs.* 1.0 × 10^{-7}). In a previous large GWAS of cardiometabolic traits, the *ADH4* rs6858148 variant was associated with waist-to-hip ratio (p value = 5.2 × 10^{-4}),[44] whereas no association was found with MASLD,[10] steatohepatitis,[30] waist circumference,[45] VAT, or abdominal SAT[32] (Table S4).

Studies on liver volume genetics have reported that liver volume is associated with a higher risk of type 2 diabetes but not type 1 diabetes,[46] and that liver volume is influenced by genetic factors related to abdominal fat distribution.[47] We found the liver volume-associated variants near *ARID1A* and

GCKR to be associated with liver function enzymes, lipid levels, and glucose metabolism, as previously reported.[48,49] Moreover, variants at the *CENPW*, *PPP1R3B*, and *PDIA3* loci showed associations with glucose, CRP, and cholesterol markers, which is in agreement with previous findings.[50–52] Imiomics results for liver volume variants were heterogeneous. Previous studies on liver volume genetics are limited[13] and, to the best of our knowledge, have not been studied in relation to fat content or tissue volume at a granular level. Previous research suggests that *PPP1R3B* may be involved in hepatic glycogen synthesis and may contribute to variations in liver phenotypes, including glycogen storage and bile acid composition, which in turn may affect liver volume.[53,54] The liver volume variant near *LPAR2* was positively associated with liver fat fraction and likely represents the same locus as the liver fat variants in the *TM6SF2* locus, which were in moderate LD and showed similar patterns of associations. The *GCKR*, *CENPW,* and *PDIA3* genes have been previously reported to have pleiotropic effects,[55–57] and our findings on their non-specificity across body maps further support these effects. For *GCKR* and *CENPW* variants, we found opposing signals in men and women, where *GCKR* variant was positively associated with liver volume only in men and negatively with skeletal muscle in women, and *CENPW* variant positively associated with liver volume only in women and positively with skeletal muscle more prominently in men.

A strength of our study is its innovative use of neck-to-knee voxel-based MRI maps (Imiomics), to provide high spatial 3D resolution and a detailed understanding of liver fat and liver volume genetic variants across different body compartments and organs, while offering valuable insights into metabolic and sex-specific genetic effects. Although liver fat content was assessed through both MRI measures and an automated neural network trained on neck-to-knee MRI images, a strong correlation (R^2 = 0.94) with the UK Biobank reference method confirms the reliability of our measures. However, limitations include the exclusive focus on participants of European ancestry, potentially limiting the generalizability of the findings. The volunteer-based nature of UK Biobank introduces a selection bias, which may limit the external validity of our findings and their generalizability to the broader population. Individuals who died before the imaging visit were not included in the study, which means that our study population might be biased toward a healthier sample. A limitation is that results could not be validated in other studies outside the UK Biobank. Further studies in other population groups with imaging are needed to confirm our findings, especially regarding the liver volume and Imiomics results. Despite the high-quality imaging data, there remains a possibility of bias attributable to MRI artifacts or segmentation errors. Additionally, as Imiomics is an emerging method, the extent of type I errors remains uncertain. Furthermore, we did not directly assess the predictive value of genetic and Imiomics-based phenotypes for clinical outcomes such as liver fibrosis, cirrhosis, cardiometabolic disease, or mortality. However, our findings provide a foundation for future studies to integrate longitudinal data and evaluate whether these genetic and imaging traits are associated with disease progression and patient outcomes – an essential step toward advancing precision medicine applications of Imiomics.

In summary, liver fat-associated genetic variants were largely specifically associated with hepatic fat and not extrahepatic. Further, liver fat variants were associated with markers of inflammation and liver injury and negatively linked to lipid biomarkers. Liver volume-associated genetic variants showed broader organ and tissue effects, including sex-specific impact on organ volume and fat, along with diverse biomarker associations. These results contribute to our understanding of how liver trait-associated variants can have sex-specific and heterogeneous effects on organ and body composition, with diverse cardiometabolic implications.

Affiliations

[1]*Molecular Epidemiology, Department of Medical Sciences, Uppsala University, Uppsala, Sweden;* [2]*Preventive Medicine Division, Harvard Medical School, Brigham and Women's Hospital, Boston, MA, United States;* [3]*School of Natural Sciences, Technology and Environmental Studies, Södertörn University, Huddinge, Sweden;* [4]*Novo Nordisk Foundation Center for Basic Metabolic Research, Faculty of Health and Medical Sciences, University of Copenhagen, Copenhagen, Denmark;* [5]*Novo Nordisk Foundation Center for Genomic Mechanisms of Disease, Broad Institute of MIT and Harvard, Cambridge, MA, United States;* [6]*Radiology, Department of Surgical Sciences, Uppsala University, Uppsala, Sweden;* [7]*Antaros Medical AB, Mölndal, Sweden;* [8]*Medical Epidemiology, Department of Surgical Sciences, Uppsala University, Uppsala, Sweden;* [9]*CIBER Cardiovascular Diseases (CIBERCV), Instituto de Salud Carlos III, Madrid, Spain;* [10]*Department of Information Technology, Uppsala University, Uppsala, Sweden;* [11]*Big Data Institute at the Li Ka Shing Centre for Health Information and Discovery, University of Oxford, Oxford, UK;* [12]*Wellcome Centre for Human Genetics, University of Oxford, Oxford, UK;* [13]*Clinical Epidemiology, Department of Medical Sciences, Uppsala University, Uppsala, Sweden;* [14]*Clinical Diabetology and Metabolism, Department of Medical Sciences, Uppsala University, Uppsala, Sweden*

Abbreviations

ADH4, alcohol dehydrogenase 4; ALP, alkaline phosphatase; ALT, alanine aminotransferase; APOA, apolipoprotein A; APOB, apolipoprotein B; ARID1A, AT-rich interaction domain 1A; AST, aspartate aminotransferase; CLD, chronic liver disease; COJO, conditional joint analysis; CREA, creatinine; CRP, C-reactive protein; cS2G, combined SNP-to-gene strategy; CYSC, cystatin C; DBIL, direct bilirubin; GCKR, glucokinase regulatory protein; GGT, gamma-glutamyl transferase; GWAS, genome-wide association study; HbA1c, hemoglobin A1c; IGF1, insulin-like growth factor 1; Imiomics, imaging-based phenomics; LD, linkage disequilibrium; MASH, metabolic-associated steatohepatitis; MASLD, metabolic dysfunction-associated steatotic liver disease; MRI, magnetic resonance imaging; OTG, Open Target Genetics; PDIA3, protein disulfide isomerase family A member 3; PPP1R3B, protein phosphatase 1 regulatory subunit 3B; REEP3, receptor accessory protein 3; SAT, subcutaneous adipose tissue; SHBG, sex hormone-binding globulin; SNP, single nucleotide polymorphism; TBIL, total bilirubin; TC, total cholesterol; TG, triglycerides; TNFSF10, tumor necrosis factor ligand superfamily member 10; UK Biobank, United Kingdom Biobank; VAT, visceral adipose tissue; V2G, variant-to-gene; WHR, waist-to-hip ratio.

Conflicts of interest

JK and HA are co-founders, co-owners of, and part-time employees of Antaros Medical AB, BioVenture Hub, Mölndal, Sweden. JCC receives a salary from Sirona AB. Her contribution to this manuscript was performed prior to commencing employment. GDC was affiliated with the University of Copenhagen during the conduct of this research. He is currently employed at Novo Nordisk. This work was completed before his employment and was not funded or influenced by Novo Nordisk. Other co-authors reported no potential conflicts of interest relevant to this article.

Please refer to the accompanying ICMJE disclosure forms for further details.

Authors' contributions

Obtained the funding, project administration, and supervision: JK, HA, TF. Conceptualized the study: SA, GDC, JCC, JK, HA, TF. Data curation: SA, GDC, TL, UM, NA, FM, RS, AMM, JK. Performed the formal analysis: SA, GDC, NA, UM, UH, MJRL, JCC, JK. Development or design of methodology in the creation of statistical models: SA, GDC, TL, UM, JCC, FM, RS, JK, TF. Software

development and code implementation: UM, TL, FM, RS, JK. Preparation of visualization or data presentation: SA, GDC, UM, NA, JCC, SSB, MJRL, AMM, JK. Wrote the first draft: SA, GDC, UM, UH, DN, JK, TF. Provided interpretation of the results and critical feedback on the final version of the manuscript: all authors.

Data availability statement

The code supporting this study is available at https://github.com/gerca/LiverGeneticsImiomic. Table S1 provides a detailed mapping of the UK Biobank variables used in this study, including their corresponding data fields. Researchers worldwide can access the phenotype and genotype data for the UK Biobank by applying for data access through the UK Biobank (https://www.ukbiobank.ac.uk/enable-your-research/register). Original GWAS results for liver fat and liver volume (height- and non-height adjusted) are available at the GWAS Catalog database under accession codes GCST90566817 (liver fat), GCST90566818 (liver volume, non-height adjusted), and GCST90566819 (liver volume, height adjusted).

Acknowledgements

Resources that enabled the computations and data handling in projects sens2017131 and sens2019016 are provided by the National Academic Infrastructure for Supercomputing in Sweden (NAISS) at Uppsala Multidisciplinary Center for Advanced Computational Science (UPPMAX) that is funded by the Swedish Research Council through grant agreement no. 2022-06725. Graphical abstract was created with BioRender.com.

TF received support from the European Research Council through a Starting Grant (ERC-StG-801965), the Swedish Research Council (VR, 2019-01471), and the Swedish Heart-Lung Foundation (Hjärt-Lungfonden, 2023-0687). SA was supported by the research grants from FORMAS – Early Career Grant (2020-00989), EpiHealth, and Swedish Research Council – Early Career Grant (2022-01460). JCC was funded by the Oxford Medical Research Council Doctoral Training Partnership (Oxford MRC DTP) and the Nuffield Department of Clinical Medicine, University of Oxford. JK was supported by a research grant from the Swedish Heart-Lung Foundation, EXODIAB, and the Swedish Research Council (2019-04756) and used the UK Biobank resource under application number 14237. HA was supported by grants from EXODIAB, the Swedish Heart-Lung Foundation (20200500, 2022012923) and the Swedish Research Council (2016-01040). GDC was supported by a grant from the Danish Diabetes Academy that is funded by the Novo Nordisk Foundation (NNF17SA0031406) and from the European Union's Horizon 2020 research and innovation programme under the Marie Sklodowska-Curie grant agreement No. 846502. GDC and TOK were additionally supported by Novo Nordisk Foundation grants NNF18CC0034900, NNF22OC0074128, and NNF23SA0084103.

Supplementary data

Supplementary data to this article can be found online at https://doi.org/10.1016/j.jhepr.2025.101468.

References

Author names in bold designate shared co-first authorship.

[1] Younossi ZM, Golabi P, Paik JM, et al. The global epidemiology of nonalcoholic fatty liver disease (NAFLD) and nonalcoholic steatohepatitis (NASH): a systematic review. Hepatology 2023;77:1335–1347.
[2] Byrne CD, Targher G. NAFLD: a multisystem disease. J Hepatol 2015;62:S47–S64.
[3] Ahmed A, Cule M, Bell JD, et al. Differing genetic variants associated with liver fat and their contrasting relationships with cardiovascular diseases and cancer. J Hepatol 2024;81:921–929.
[4] **Targher G**, **Byrne CD**, **Tilg H**. MASLD: a systemic metabolic disorder with cardiovascular and malignant complications. Gut 2024;73:691–702.
[5] Starekova J, Hernando D, Pickhardt PJ, et al. Quantification of liver fat content with CT and MRI: state of the art. Radiology 2021;301:250–262.
[6] Artz NS, Haufe WM, Hooker CA, et al. Reproducibility of MR-based liver fat quantification across field strength: same-day comparison between 1.5T and 3T in obese subjects. J Magn Reson Imaging 2015;42:811–817.
[7] **Patel M**, **Puangsricharoen P**, **Arshad HMS**, et al. Does providing routine liver volume assessment add value when performing CT surveillance in cirrhotic patients? Abdom Radiol (Ny) 2019;44:3263–3272.
[8] Bian H, Hakkarainen A, Zhou Y, et al. Impact of non-alcoholic fatty liver disease on liver volume in humans. Hepatol Res 2015;45:210–219.
[9] **Eslam M**, **George J**. Genetic contributions to NAFLD: leveraging shared genetics to uncover systems biology. Nat Rev Gastroenterol Hepatol 2020;17:40–52.
[10] **Chen Y**, **Du X**, Kuppa A, et al. Genome-wide association meta-analysis identifies 17 loci associated with nonalcoholic fatty liver disease. Nat Genet 2023;55:1640–1650.
[11] Ghouse J, Sveinbjornsson G, Vujkovic M, et al. Integrative common and rare variant analyses provide insights into the genetic architecture of liver cirrhosis. Nat Genet 2024;56:827–837.
[12] Margoliash J, Fuchs S, Li Y, et al. Polymorphic short tandem repeats make widespread contributions to blood and serum traits. Cell Genom 2023;3:100458.
[13] Liu Y, Basty N, Whitcher B, et al. Genetic architecture of 11 organ traits derived from abdominal MRI using deep learning. Elife 2021;10:e65554.
[14] Park SL, Li Y, Sheng X, et al. Genome-wide association study of liver fat: the multiethnic cohort adiposity phenotype study. Hepatol Commun 2020;4:1112–1123.
[15] **Haas ME**, **Pirruccello JP**, **Friedman SN**, et al. Machine learning enables new insights into genetic contributions to liver fat accumulation. Cell Genom 2021;1:100066.
[16] van der Meer D, Gurholt TP, Sønderby IE, et al. The link between liver fat and cardiometabolic diseases is highlighted by genome-wide association study of MRI-derived measures of body composition. Commun Biol 2022;5:1271.
[17] Bredella MA. Sex differences in body composition. In: Mauvais-Jarvis F, editor. Sex and gender factors affecting metabolic homeostasis, diabetes and obesity. Cham: Springer; 2017. p. 9–27.
[18] Strand R, Malmberg F, Johansson L, et al. A concept for holistic whole body MRI data analysis, Imiomics. PLoS One 2017;12:e0169966.
[19] **Bycroft C**, **Freeman C**, **Petkova D**, et al. The UK Biobank resource with deep phenotyping and genomic data. Nature 2018;562:203–209.
[20] Sudlow C, Gallacher J, Allen N, et al. UK biobank: an open access resource for identifying the causes of a wide range of complex diseases of middle and old age. Plos Med 2015;12:e1001779.
[21] Elliott P, Peakman TC, Biobank UK. The UK Biobank sample handling and storage protocol for the collection, processing and archiving of human blood and urine. Int J Epidemiol 2008;37:234–244.
[22] Littlejohns TJ, Holliday J, Gibson LM, et al. The UK Biobank imaging enhancement of 100,000 participants: rationale, data collection, management and future directions. Nat Commun 2020;11:2624.
[23] Wilman HR, Kelly M, Garratt S, et al. Characterisation of liver fat in the UK Biobank cohort. PLoS One 2017;12:e0172921.
[24] Langner T, Strand R, Ahlström H, et al. Large-scale inference of liver fat with neural networks on UK Biobank body MRI. In: Martel AL, Abolmaesumi P, Stoyanov D, et al., editors. Medical image computing and computer assisted intervention – MICCAI 2020. Cham: Springer; 2020. p. 602–611.
[25] Ronneberger O, Fischer P, Brox T. U-Net: convolutional networks for biomedical image segmentation. In: Navab N, Hornegger J, Wells WM, Frangi AF, editors. Medical image computing and computer-assisted intervention – MICCAI 2015. Cham: Springer; 2015. p. 234–241.
[26] Langner T, Östling A, Maldonis L, et al. Kidney segmentation in neck-to-knee body MRI of 40,000 UK Biobank participants. Sci Rep 2020;10:20963.
[27] Langner T, Martinez Mora A, Strand R, et al. MIMIR: deep regression for automated analysis of UK Biobank MRI scans. Radiol Artif Intell 2022;4:e210178.
[28] **Pruim RJ**, **Welch RP**, Sanna S, et al. LocusZoom: regional visualization of genome-wide association scan results. Bioinformatics 2010;26:2336–2337.
[29] Bulik-Sullivan BK, Loh PR, Finucane HK, et al. LD Score regression distinguishes confounding from polygenicity in genome-wide association studies. Nat Genet 2015;47:291–295.
[30] Kurki MI, Karjalainen J, Palta P, et al. FinnGen provides genetic insights from a well-phenotyped isolated population. Nature 2023;613:508–518.
[31] Yang J, Ferreira T, Morris AP, et al. Conditional and joint multiple-SNP analysis of GWAS summary statistics identifies additional variants influencing complex traits. Nat Genet 2012;44:369–375. S361–3.
[32] **Agrawal S**, **Wang M**, Klarqvist MDR, et al. Inherited basis of visceral, abdominal subcutaneous and gluteofemoral fat depots. Nat Commun 2022;13:3771.

[33] Yang Z, Wang C, Liu L, et al. CARMA is a new Bayesian model for fine-mapping in genome-wide association meta-analyses. Nat Genet 2023;55:1057–1065.
[34] Gazal S, Weissbrod O, Hormozdiari F, et al. Combining SNP-to-gene linking strategies to identify disease genes and assess disease omnigenicity. Nat Genet 2022;54:827–836.
[35] Feizi A, Ray K. otargen: GraphQL-based R package for tidy data accessing and processing from Open Targets Genetics. Bioinformatics 2023;39: btad441.
[36] Lind L, Strand R, Michaëlsson K, et al. Relationship between endothelium-dependent vasodilation and fat distribution using the new "imiomics" image analysis technique. Nutr Metab Cardiovasc Dis 2019;29:1077–1086.
[37] **Li Y**, **van den Berg EH**, Kurilshikov A, et al. Genome-wide studies reveal genetic risk factors for hepatic fat content. Genomics, Proteomics, Bioinformatics 2024;22:qzae031.
[38] **Elmansoury N**, **Megahed AA**, Kamal A, et al. Relevance of PNPLA3, TM6SF2, HSD17B13, and GCKR variants to MASLD severity in an Egyptian population. Genes (Basel) 2024;15:455.
[39] **Palmer ND**, **Kahali B**, **Kuppa A**, et al. Allele-specific variation at APOE increases nonalcoholic fatty liver disease and obesity but decreases risk of Alzheimer's disease and myocardial infarction. Hum Mol Genet 2021;30:1443–1456.
[40] Anstee QM, Day CP. The genetics of nonalcoholic fatty liver disease: spotlight on PNPLA3 and TM6SF2. Semin Liver Dis 2015;35:270–290.
[41] Mahdessian H, Taxiarchis A, Popov S, et al. TM6SF2 is a regulator of liver fat metabolism influencing triglyceride secretion and hepatic lipid droplet content. Proc Natl Acad Sci U S A 2014;111:8913–8918.
[42] **Liu DJ**, **Peloso GM**, **Yu H**, et al. Exome-wide association study of plasma lipids in >300,000 individuals. Nat Genet 2017;49:1758–1766.
[43] Kempinska-Podhorodecka A, Krawczyk M, Klak M, et al. Healthy PNPLA3 risk allele carriers present with unexpected body fat composition. A study of one thousand subjects. J Gastrointestin Liver Dis 2014;23:33–37.
[44] Pulit SL, Stoneman C, Morris AP, et al. Meta-analysis of genome-wide association studies for body fat distribution in 694 649 individuals of European ancestry. Hum Mol Genet 2019;28:166–174.
[45] **Elsworth B**, **Lyon M**, Alexander T, et al. The MRC IEU OpenGWAS data infrastructure. BioRxiv 2020. https://doi.org/10.1101/2020.08.10.244293.
[46] Martin S, Sorokin EP, Thomas EL, et al. Estimating the effect of liver and pancreas volume and fat content on risk of diabetes: a Mendelian randomization study. Diabetes Care 2022;45:460–468.
[47] **Martin S**, **Cule M**, Basty N, et al. Genetic evidence for different adiposity phenotypes and their opposing influences on ectopic fat and risk of cardiometabolic disease. Diabetes 2021;70:1843–1856.
[48] **Qu YL**, **Deng CH**, Luo Q, et al. Arid1a regulates insulin sensitivity and lipid metabolism. EBioMedicine 2019;42:481–493.
[49] Du X, DeForest N, Majithia AR. Human genetics to identify therapeutic targets for NAFLD: challenges and opportunities. Front Endocrinol (Lausanne) 2021;12:777075.
[50] Sahini N, Borlak J. Genomics of human fatty liver disease reveal mechanistically linked lipid droplet-associated gene regulations in bland steatosis and nonalcoholic steatohepatitis. Transl Res 2016;177:41–69.
[51] Klimentidis YC, Arora A, Newell M, et al. Phenotypic and genetic characterization of lower LDL cholesterol and increased type 2 diabetes risk in the UK Biobank. Diabetes 2020;69:2194–2205.
[52] Creasy KT, Mehta MB, Park J, et al. PPP1R3B is a metabolic switch that shifts hepatic energy storage from lipid to glycogen. Sci Adv 2025;11:eado3440.
[53] Stender S, Smagris E, Lauridsen BK, et al. Relationship between genetic variation at PPP1R3B and levels of liver glycogen and triglyceride. Hepatology 2018;67:2182–2195.
[54] **Männistö V**, **Kaminska D**, Käkelä P, et al. Protein phosphatase 1 regulatory subunit 3B genotype at rs4240624 has a major effect on gallbladder bile composition. Hepatol Commun 2021;5:244–257.
[55] **Yeh KH**, **Hsu LA**, Teng MS, et al. Pleiotropic effects of common and rare GCKR exonic mutations on cardiometabolic traits. Genes (Basel) 2022;13:491.
[56] **Han X**, **Qassim A**, An J, et al. Genome-wide association analysis of 95 549 individuals identifies novel loci and genes influencing optic disc morphology. Hum Mol Genet 2019;28:3680–3690.
[57] **Ghouse J**, **Tragante V**, Ahlberg G, et al. Genome-wide meta-analysis identifies 93 risk loci and enables risk prediction equivalent to monogenic forms of venous thromboembolism. Nat Genet 2023;55:399–409.

Keywords: Genetic variation; Metabolic dysfunction-associated steatotic liver disease; Chronic liver disease; Metabolic disease.
Received 20 September 2024; received in revised form 19 May 2025; accepted 27 May 2025; Available online 2 June 2025

Journal of Hepatology, Volume 7

Supplemental information

Impact of genetic variants linked to liver fat and liver volume on MRI-mapped body composition

Shafqat Ahmad, Germán D. Carrasquilla, Taro Langner, Uwe Menzel, Nouman Ahmad, Sergi Sayols-Baixeras, Koen F. Dekkers, Beatrice Kennedy, Filip Malmberg, Ulf Hammar, María J. Romero-Lado, Jenny C. Censin, Diem Nguyen, Andrés Martínez Mora, Tuomas O. Kilpeläinen, Lars Lind, Jan W. Eriksson, Robin Strand, Joel Kullberg, Håkan Ahlström, and Tove Fall

Impact of genetic variants linked to liver fat and liver volume on MRI-mapped body composition

Shafqat Ahmad, Germán D Carrasquilla, Taro Langner, Uwe Menzel, Nouman Ahmad, Sergi Sayols-Baixeras, Koen F Dekkers, Beatrice Kennedy, Filip Malmberg, Ulf Hammar, María J Romero-Lado, Jenny C Censin, Diem Nguyen, Andrés Martínez Mora, Tuomas O Kilpeläinen, Lars Lind, Jan W Eriksson, Robin Strand, Joel Kullberg, Håkan Ahlström, Tove Fall

Table of contents

Supplementary materials and methods……………………………………………………2

Supplementary figures………………………………………………………………………7

Supplementary tables……………………………………………………………….……..27

Supplementary movies……………………………………………………………….……27

Supplementary references…………………………………………………………….…..28

Supplementary materials and methods

Anthropometric and cardiometabolic markers

Height, weight, waist, and hip circumference were objectively measured following standardized protocols. At baseline, body mass index (BMI) was calculated using weight and height (kg/m^2), and waist-hip ratio (WHR) was calculated using waist in centimeters divided by hip in centimeters. Biochemical markers used in this study were assessed in serum samples collected during the baseline assessment. Biochemical measurements were performed using a Beckman Coulter AU5800 analyzer, except for lipoprotein A [LPA, Data-Field 30790], which was measured with a Randox analyzer, and Cystatin C [CYSC, Data-Field 30720], which was measured using a Siemens analyzer.[1] Glycated hemoglobin [HbA1c, Data-Field 30750] was measured from red blood cells using a Bio-Rad Variant II Turbo analyzer.[2]

Clinical diagnosis of MASLD and chronic liver disease

Clinical diagnosis of metabolic dysfunction-associated steatotic liver disease (MASLD) was assessed from health inpatient register diagnoses coded according to the International Classification of Disease version 10 (ICD-10) code K760. Chronic liver disease (CLD) was assessed using ICD-10 K702-704, K717, K721, K74, K740-746, and ICD-9 subtypes (27103; 4562; 571; 5712; 5715; 57150; 57151; 57158; 57159; 5716) which covers an extensive range of liver diseases, including alcohol-induced cirrhosis and primary biliary cirrhosis as has been described previously.[3] The diagnosis of participants, encompassing both prevalent and incident cases, was considered before and after the imaging data.

Liver fat and volume phenotype regression analysis

Before performing the genome-wide association study (GWAS) analysis of liver fat and liver volume, both traits were transformed into inverse-normal traits. In this inverse-normal transformation (INT) two-step approach, the phenotype (trait) was regressed on the covariates in the first step, and the residuals emerging from that linear regression were then inverse-normal

transformed, applying a rank-based algorithm. In the second step, the inverse-normal transformed residuals were regressed on the variant and the covariates to reveal genotype-phenotype associations.

Imiomics analysis

The analysis of the image data with respect to liver fat-associated genetic variant data was completed with an algorithm called "Imiomics".[4] MRI images comprising both water and fat signals for each subject were divided into six stations covering the neck to the knee. The different water and fat signal stations were pre-fused into one neck-to-knee volume per subject, one for the water signal and one for the fat signal. Finally, the water and fat volumes were combined into a fat fraction image, where each voxel represents the relative amount of fat the subject has in that specific region.[4] Here, the fat fraction maps of all subjects were split into man and woman, deformed against a template male subject and a template female subject, respectively. For each subject, a "volume image" or "Jacobian image" with voxel-wise information about the magnitude of the deformation in each region was obtained, together with a "fat fraction image" consisting of the original fat fraction image of each subject deformed into the template subject.[5] The voxels of all the "volume images" and "fat fraction images" were regressed against liver fat- or liver volume-associated single nucleotide polymorphisms (SNPs), adjusting for a set of covariates (age, age squared, total body fat, height, and 20 principal components). These computations provide a map with the voxel-wise regression slope of all regions in the body against the SNPs of interest for men and women and the "volume images" and the "fat fraction images," also known as "Imiomics maps". Consequently, the maps obtained for the volume images show how much the tissue volume across the neck-to-knee region vary by allele in the selected SNPs, while the maps obtained for the fat fraction images depict how much the fat across the neck-to knee region changes by allele of the selected SNPs.

Assessment of alcohol intake

Alcohol intake (measured in grams per week) was assessed through food frequency questionnaires (FFQs). Variables included drinking status (never, previous, current, prefer not to answer) (Data-Field: 20117-0.0) and current alcohol intake (daily or almost daily, three or four times a week, once or twice a week, one to three times a month, special occasion only, never, prefer not to answer) (Data-Field: 1558). Study participants who reported a current alcohol intake frequency of at least once or twice a week were asked to estimate their average weekly alcohol intake of different alcoholic beverages (e.g., red wine, white wine, champagne, beer, cider, spirits, fortified wine) (Data-Fields: 1568, 1578, 1588, 1598, 1608). We then derived the average current intake of alcohol, which was estimated by combining different measures of the alcoholic beverages mentioned above.[6, 7] The FFQs used the following measurements of each of the alcoholic drink types: measures of spirits, glasses for wine, and pints for beer/cider, which are estimated to be equivalent to 1, 2, and 2.5 units, respectively.[6, 7] Study participants who reported a current alcohol intake frequency of "one to three times a month," "special occasions only," or "never" were assumed to have a weekly alcohol consumption volume of zero.[6, 7]

GWAS for liver volume loci without adjustment for height

In the GWAS for liver volume without adjustment for height, we identified the *CENPW* rs1490384 variant (in high linkage disequilibrium (LD), R^2=0.81, D-prime= 0.98 with rs853966 that we discovered in liver volume GWAS with height adjustment), which is a regulatory region variant, and confirmed eight previously identified genetic variants (**Table S5**). The genomic inflation factor was λGC=1.06.

Genetic correlation for liver fat and liver volume across hepatic disease traits

For the assessment of genetic correlations, we utilized linkage disequilibrium score regression (LDSC) analyses, incorporating data from our internal GWAS on liver fat and liver volume as

inputs. We incorporated GWAS data on hepatic diseases from the FinnGen project's Freeze 9 release, dated March 2024 (https://r9.finngen.fi/).[8] These diseases included MASLD, metabolic-associated steatohepatitis (MASH), fibrosis and cirrhosis, and hepatocellular carcinoma. Employing the LDSC method[9] and utilizing the GenomicSEM package version 0.0.5, we estimated genetic correlations between our GWAS on liver fat and liver volume and the aforementioned hepatic diseases. This approach considered disease prevalence data from the FinnGen database and implemented quality control measures to mitigate result inflation. The findings were visualized in a heatmap correlation plot using the ggplot2 package (version 3.4.4) (**Fig. S4**).

Pruning and conditional analysis

We performed linkage disequilibrium (LD) clumping analysis to identify the independent GWAS genetic variants. In the LD clumping approach, two GWAS significant hits approaches were applied (primary with SNPs with *p*-value $<5\times10^{-08}$ and *p*-value $<5\times10^{-06}$) using a screening window of 5 Mb adjacent to the first index SNP. In the COJO analysis, we identified genetic regions with one or more SNPs with *p*-value $<5\times10^{-08}$ using a screening window of 5 Mb adjacent to the first index SNP. New association statistics were calculated for all other SNPs, conditioning on the effect of the index SNP. A SNP with a *p*-value $<5\times10^{-08}$ conditional on the index SNP was regarded as significantly associated with our outcome trait.

Fine mapping and gene prioritization for liver volume and liver fat loci

In the gene prioritization phase, we focused on the identified significant and independent genetic variants associated with the liver fat and liver volume GWAS, and selected 4 and 10 variants, respectively (**Table 2**). Our prioritization strategies entailed identifying target genes linked to these significant SNPs. These strategies included the retrieval of the nearest candidate gene and the selection of the highest variant-to-gene (V2G) candidate by Open Target Genetics (OTG) utilizing the R package otargen (version 1.1.0),[10] and the implementation of combined

SNP-to-gene strategies (cS2G).[11] The cS2G approach is a composite method integrating seven SNP-to-gene strategies, which involves extensive analysis and fine-mapping to identify putative causal variants within the regions surrounding initial SNP candidates.[11] Fine-mapping using Causal Association and Regression Model Averaging (CARMA) was performed by extending the regions around initial candidate SNPs by ±500,000 bp.[12] After fine mapping, genetic variants with a posterior inclusion probability (PIP) of less than 0.1 were excluded (**Table S2**). This refined set of variants was subjected to further prioritization using the cS2G strategies. The cS2G-reported links were those with a cS2G score greater than 0.5, either with the candidate SNP or a proxy (R^2>0.8), thereby narrowing down to the most likely causal genes. This is why some initially identified variants have more than one cS2G SNP-to-gene link, as all proxy-to-gene links fulfilling the abovementioned conditions were also reported.

Supplementary figures

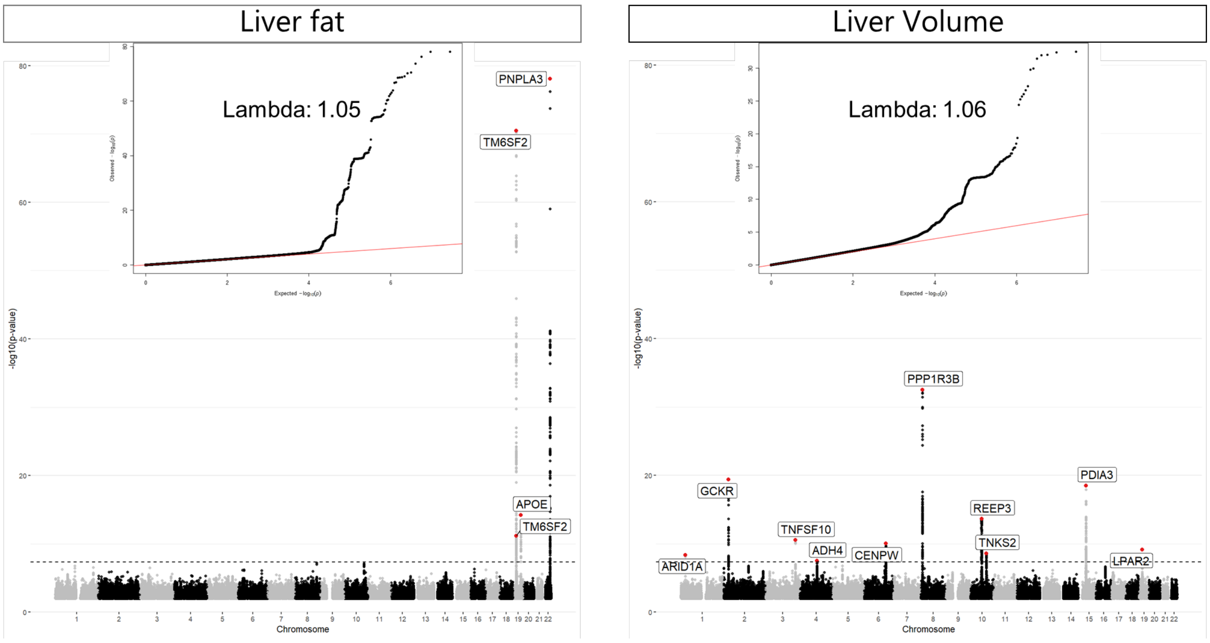

Fig. S1. Manhattan and Q-Q plots from GWAS of liver fat and liver volume in the UK Biobank cohort. Manhattan plot illustrating GWAS of liver fat (n=27,243) and liver volume (n=24,752) in the individuals from the UK Biobank. The left panel presents the Manhattan plot and Q-Q plot of liver fat GWAS, and the right panel presents the Manhattan and Q-Q plot of liver volume GWAS. In the Manhattan plots, the x-axis is the chromosomal position, and the y-axis is the significance of association for each variant in log10(*p*-values). Level of significance is set at *p*-value $<5\times10^{-08}$. The Q-Q plots of GWAS of liver fat and liver volume show the deviation from the diagonal. The genomic inflation factor was (λGC=1.05) and (λGC=1.06) for the liver fat and liver volume GWAS, respectively.

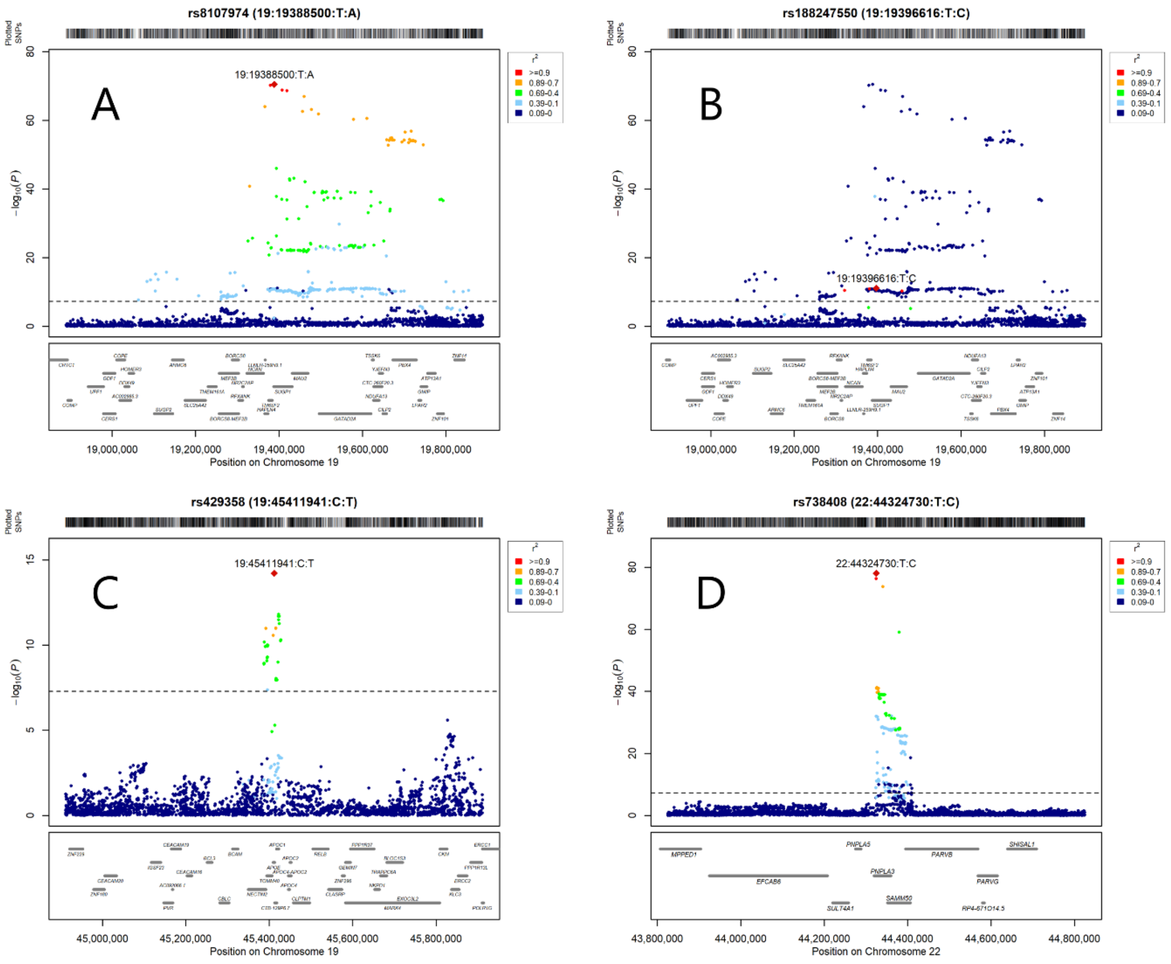

Fig. S2. Regional association plots for liver fat loci. The nearest gene and variant identification are presented for liver fat-associated genetic variants. *TM6SF2* rs8107974 (A), *TM6SF2* rs188247550 (B), *APOE* rs429358 (C), and *PNPLA3* rs738408 (D). The correlation strength between proxies and lead SNPs (R^2) are indicated for each variant in log10(*p*-values) along the chromosome.

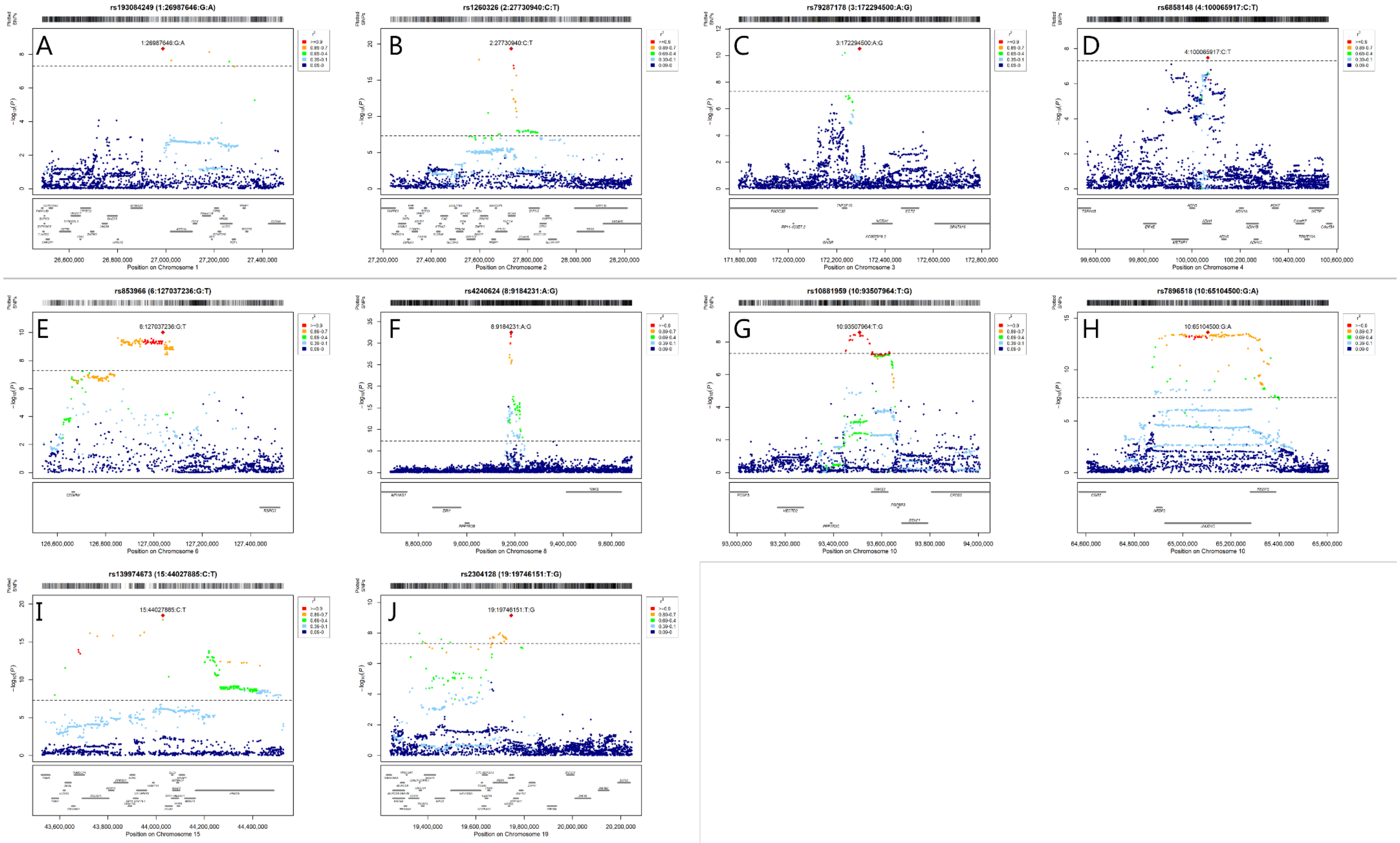

Fig. S3. Regional association plot of liver volume loci. The nearest gene and variant identification are presented for liver volume-associated genetic variants. *ARID1A* rs193084249 (A), *GCKR* rs1260326 (B), *TNFSF10* rs79287178 (C), *ADH4* rs6858148 (D), *CENPW* rs853966 (E), *PPP1R3B* rs4240624 (F), *TNKS2* rs10881959 (G), *REEP3* rs7896518 (H), *PDIA3* rs139974673 (I), and *LPAR2* rs2304128 (J). The correlation strength between proxies and lead SNPs (R^2) are indicated for each variant in log10(*p*-values) along the chromosome.

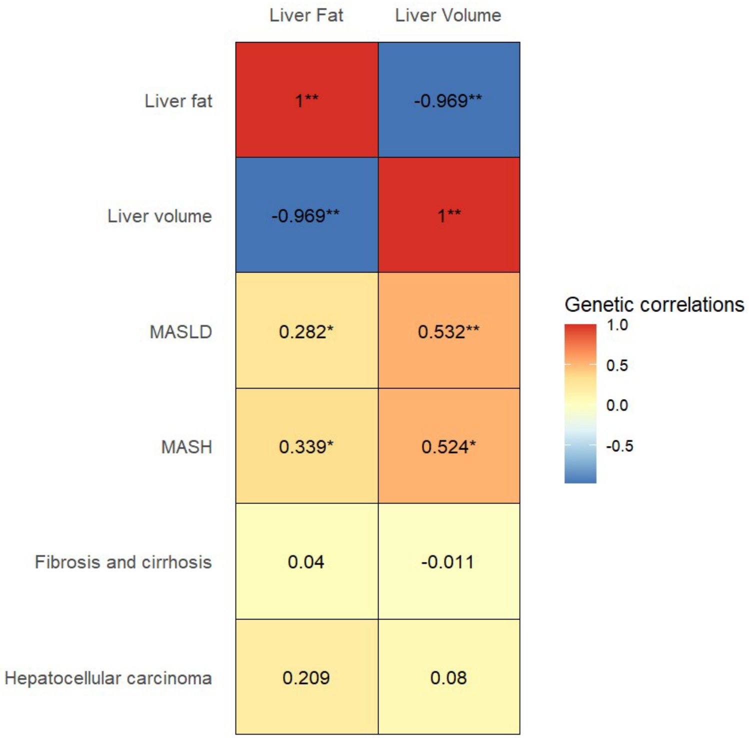

Fig. S4. Genetic correlation for liver fat and liver volume across liver diseases. A single asterisk (*) indicates a significant genetic correlation at *p*-value < 0.05. Double asterisks (**) indicate a significant genetic correlation at the Bonferroni-corrected *p*-value < 0.0042. Blue shading represents negative genetic correlations, and red shading represents positive correlations, with increasing color intensity reflecting increasing correlation strength.

Gene	*TM6SF2* (rs8107974)				*TM6SF2* (rs188247550)				*APOE* (rs429358)				*PNPLA3* (rs738408)			
**Volume or fat fraction**	**Volume**		**Fat fraction**		**Volume**		**Fat fraction**		**Volume**		**Fat fraction**		**Volume**		**Fat fraction**	
**sex**	**Men**	**Women**	**Men**	**Women**	**Men**	**Women**	**Men**	**Women**	**Men**	**Women**	**Men**	**Women**	**Men**	**Women**	**Men**	**Women**
**Adipose tissue**																
Thorax SAT		+ *														
Abdominal anterior SAT		+ *														
Abdominal posterior SAT		- *														
VAT																
Gluteal SAT		- *				- *								+ *		
Thigh SAT	-- *					--*							--*			
**Organs**																
Lungs																
Heart																
Liver			+++	++			+	+			+	+			++	++
Kidneys																
Other																
**Skeleton**																
Spine																
Pelvis																
Femur																
**Skeletal muscle**																
Thorax																
Abdomen																
Gluteus																
Thigh										+ *						
**Specific notes and captions**	* Regional				* Regional				* Regional				* Regional			

Color scale

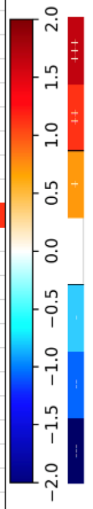

Fig. S5. Summary findings for liver fat genetic variants across neck-to-knee voxel maps for tissue volume and fat fraction. The nearest gene and the variant identification are presented. Tissue volume and fat fraction are presented separately for men and women for different parts of the body. SAT, subcutaneous adipose tissue. The +, ++, +++ and -- indicate the effect size. The * indicates regional associations. Significant associations (p-value <2.8×10^{-3}) are color coded from negative (blue) to positive (red).

Gene	*ARID1A* (rs193084249)				*GCKR* (rs1260326)				*TNFSF10* (rs79287178)				*ADH4* (rs6858148)				*CENPW* (rs853966)			
Volume or fat content	Volume		Fat fraction		Volume		Fat fraction		Volume		Fat fraction		Volume		Fat fraction		Volume		Fat fraction	
Sex	Men	Women	Men	Women	Men	Women	Men	Women	Men	Women	Men	Women	Men	Women	Men	Women	Men	Women	Men	Women
**Adipose tissue**																				
Thorax SAT																				
Abdominal anterior SAT																				
Abdominal posterior SAT																				
VAT														++*						
Gluteal SAT																				
Thigh SAT					–*															
**Organs**																				
Lungs																				
Heart										+*								+*		
Liver	+*				++		+*		+*	+*			+*					+*		
Kidneys																				
**Skeleton**																				
Spine																–*			-*	
Pelvis																			-*	
Femur																			–*	-*
**Skeletal muscle**																				
Thorax						–*											+*			
Abdomen													+*				+*			
Gluteus						–*		+*									++*	+*		
Thigh						–*				+*							+*	+*		
**Specific notes and captions**	* Regional				* Regional		* Regional		* Regional				* Regional		* Discs		* Regional		* Regional	

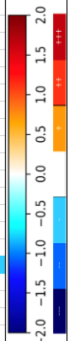

Gene	*PPP1R3B* (rs4240624)				*TNKS2* (rs10881959)				*REEP3* (rs7896518)				*PDIA3* (rs139974673)				*LPAR2* (rs2304128)			
Volume or fat content	Volume		Fat fraction		Volume		Fat fraction		Volume		Fat fraction		Volume		Fat fraction		Volume		Fat fraction	
Sex	Men	Women	Men	Women	Men	Women	Men	Women	Men	Women	Men	Women	Men	Women	Men	Women	Men	Women	Men	Women
**Adipose tissue**																				
Thorax SAT																	+*			
Abdominal anterior SAT																		+*		
Abdominal posterior SAT																				
VAT																				
Gluteal SAT														-*						
Thigh SAT																				
**Organs**																				
Lungs																				
Heart									++				+*							
Liver	++	++			++*				++	++			++	++*		+			++	++
Kidneys																				
**Skeleton**																				
Spine																				
Pelvis																				
Femur																				
**Skeletal muscle**																				
Thorax	+*																			
Abdomen																				
Gluteus	+*																			
Thigh																				
**Specific notes and captions**	* Regional				* Regional								* Regional				* Regional			

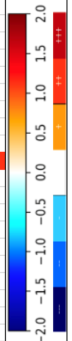

Fig. S6. Summary of findings for liver volume genetic variants across neck-to-knee voxel maps for tissue volume and fat fraction. The nearest gene and the variant identification are presented. Tissue volume and fat fraction are presented separately for men and women for different parts of the body. SAT, subcutaneous adipose tissue. The +, ++, +++ and -- indicate the effect size. The * indicate regional associations. Significant associations (p-value $<2.8\times10^{-3}$) are color coded from negative (blue) to positive (red).

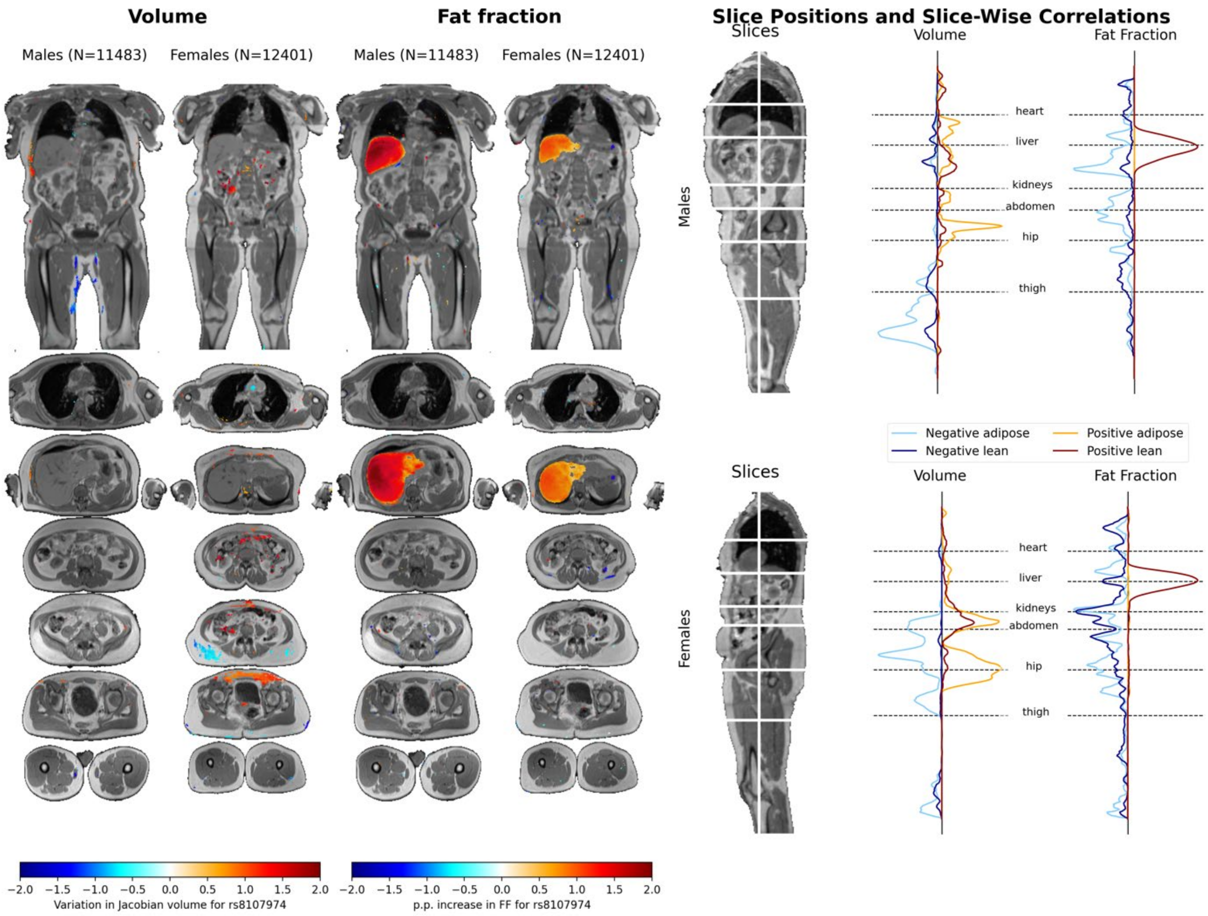

Fig. S7. Imiomics maps for the *TM6SF2* rs8107974 variant that quantifies its association to tissue volume and fat fraction voxel-wise throughout the neck-to-knee region. The resulting images show, from left to right, the associations to volume for males, the associations to volume for females, the associations to fat fractions for males, and the associations to fat fractions for females. Color mapping is used to visualize the quantified associations where significant (*p*-value $<2.8 \times 10^{-3}$). For visualization purposes clipped beta values (from the lower 1% to the highest 1%) were for each experiment linearly rescaled between -2 to +2 maintaining the sign of the association. Non-significant regions are not colored; but instead, a mix of the water and fat magnetic resonance images for the chosen male and female template subjects are shown to allow orientation in the body regions. The first row of images represents coronal slices of the maps, while the remaining rows represent axial slices of those maps in levels of locations of interest (heart, liver, kidneys, abdomen, hip, and thighs). The mid-sagittal slices on the right-hand side of the plots illustrate the visualized slices. The plots on the right-hand side of the collage figures represent the relative amount, from neck to knee, of significant positive and negative associations in the 3D data. The associations are reported separately from lean tissues (where the water signal is stronger than the fat signal) and from adipose tissue (where the fat signal is stronger than the water signal). Full 3D associations are visualized in **Movie S1** (axial and coronal planes).

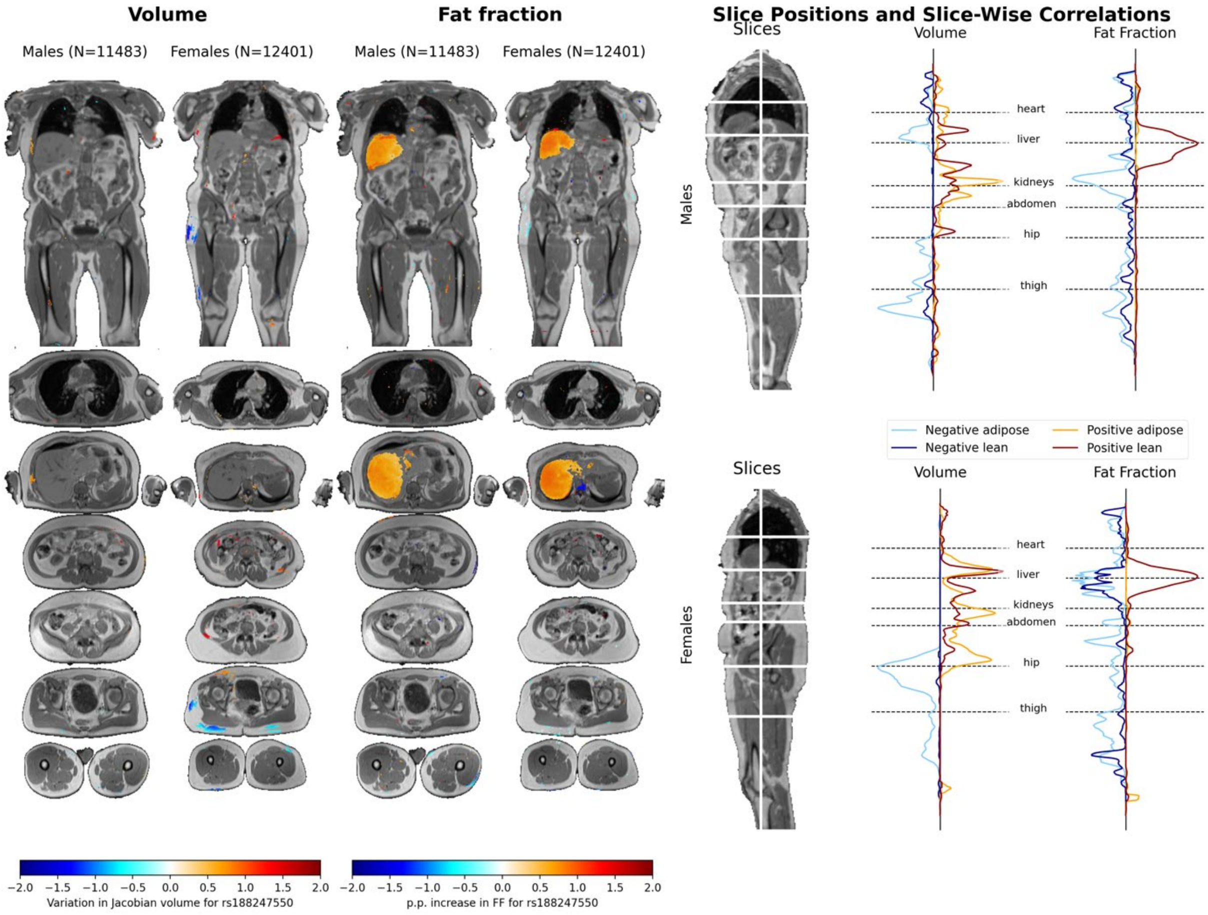

Fig. S8. Imiomics maps for the *TM6SF2* rs188247550 variant that quantifies its association to tissue volume and fat fraction voxel-wise throughout the neck-to-knee region. The resulting images show, from left to right, the associations to volume for males, the associations to volume for females, the associations to fat fractions for males, and the associations to fat fractions for females. Color mapping is used to visualize the quantified associations where significant (p-value <2.8×10^{-3}). For visualization purposes clipped beta values (from the lower 1% to the highest 1%) were for each experiment linearly rescaled between -2 to +2 maintaining the sign of the association. Non-significant regions are not colored; but instead, a mix of the water and fat magnetic resonance images for the chosen male and female template subjects are shown to allow orientation in the body regions. The first row of images represents coronal slices of the maps, while the remaining rows represent axial slices of those maps in levels of locations of interest (heart, liver, kidneys, abdomen, hip, and thighs). The mid-sagittal slices on the right-hand side of the plots illustrate the visualized slices. The plots on the right-hand side of the collage figures represent the relative amount, from neck to knee, of significant positive and negative associations in the 3D data. The associations are reported separately from lean tissues (where the water signal is stronger than the fat signal) and from adipose tissue (where the fat signal is stronger than the water signal). Full 3D associations are visualized in **Movie S2** (axial and coronal planes).

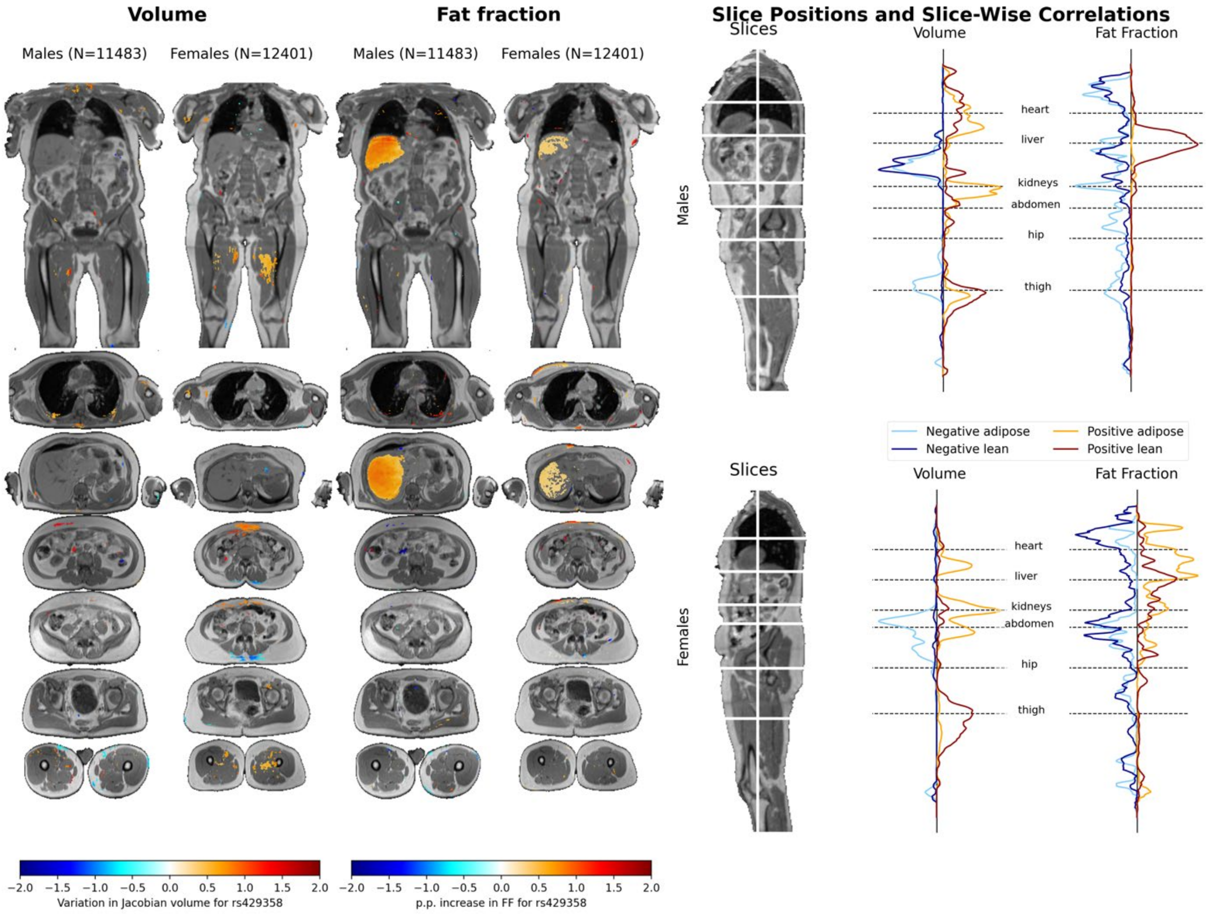

Fig. S9. Imiomics maps for the *APOE* rs429358 variant that quantifies its association to tissue volume and fat fraction voxel-wise throughout the neck-to-knee region. The resulting images show, from left to right, the associations to volume for males, the associations to volume for females, the associations to fat fractions for males, and the associations to fat fractions for females. Color mapping is used to visualize the quantified associations where significant (p-value $<2.8\text{x}10^{-3}$). For visualization purposes clipped beta values (from the lower 1% to the highest 1%) were for each experiment linearly rescaled between -2 to +2 maintaining the sign of the association. Non-significant regions are not colored; but instead, a mix of the water and fat magnetic resonance images for the chosen male and female template subjects are shown to allow orientation in the body regions. The first row of images represents coronal slices of the maps, while the remaining rows represent axial slices of those maps in levels of locations of interest (heart, liver, kidneys, abdomen, hip, and thighs). The mid-sagittal slices on the right-hand side of the plots illustrate the visualized slices. The plots on the right-hand side of the collage figures represent the relative amount, from neck to knee, of significant positive and negative associations in the 3D data. The associations are reported separately from lean tissues (where the water signal is stronger than the fat signal) and from adipose tissue (where the fat signal is stronger than the water signal). Full 3D associations are visualized in **Movie S3** (axial and coronal planes).

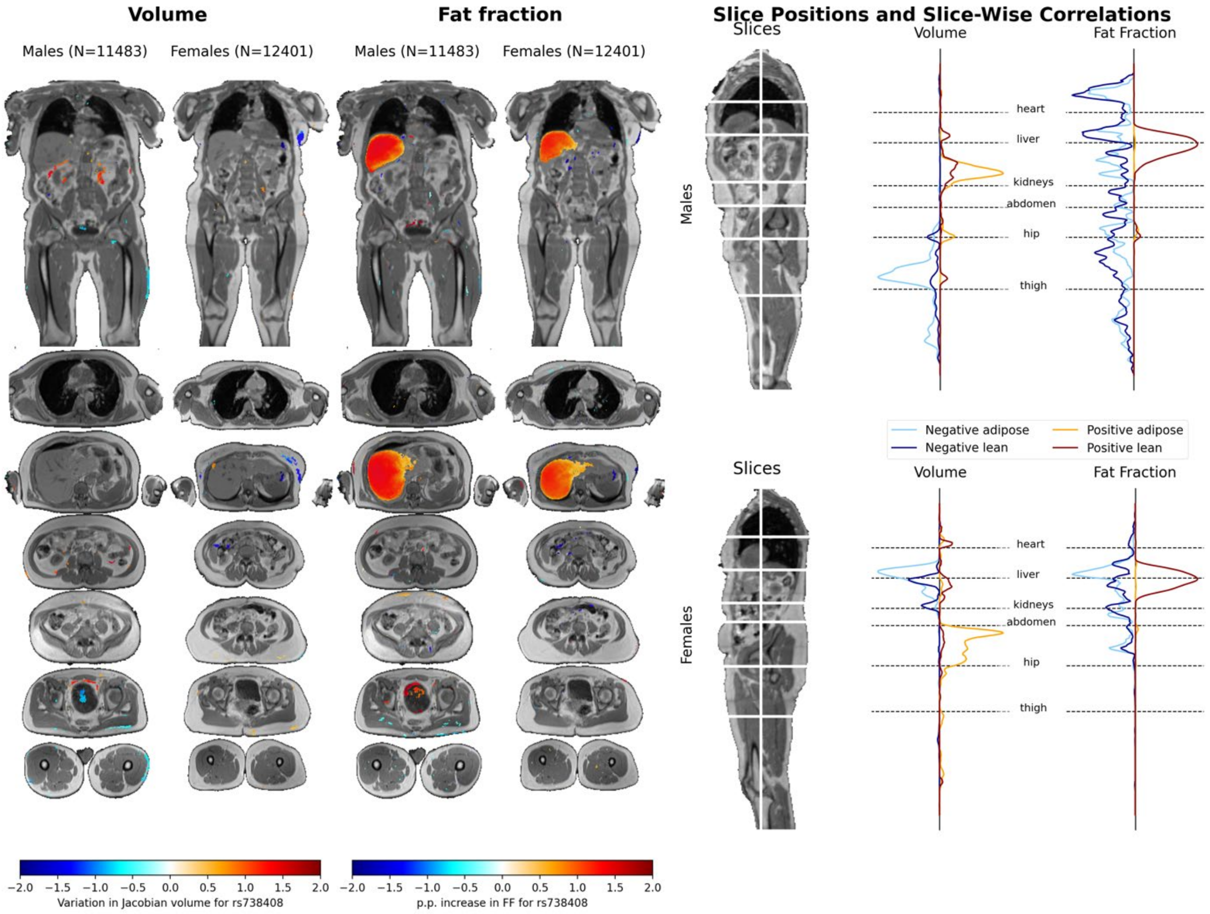

Fig. S10. Imiomics maps for the *PNPLA3* rs738408 variant that quantifies its association to tissue volume and fat fraction voxel-wise throughout the neck-to-knee region. The resulting images show, from left to right, the associations to volume for males, the associations to volume for females, the associations to fat fractions for males, and the associations to fat fractions for females. Color mapping is used to visualize the quantified associations where significant (*p*-value <2.8×10^{-3}). For visualization purposes clipped beta values (from the lower 1% to the highest 1%) were for each experiment linearly rescaled between -2 to +2 maintaining the sign of the association. Non-significant regions are not colored; but instead, a mix of the water and fat magnetic resonance images for the chosen male and female template subjects are shown to allow orientation in the body regions. The first row of images represents coronal slices of the maps, while the remaining rows represent axial slices of those maps in levels of locations of interest (heart, liver, kidneys, abdomen, hip, and thighs). The mid-sagittal slices on the right-hand side of the plots illustrate the visualized slices. The plots on the right-hand side of the collage figures represent the relative amount, from neck to knee, of significant positive and negative associations in the 3D data. The associations are reported separately from lean tissues (where the water signal is stronger than the fat signal) and from adipose tissue (where the fat signal is stronger than the water signal). Full 3D associations are visualized in **Movie S4** (axial and coronal planes).

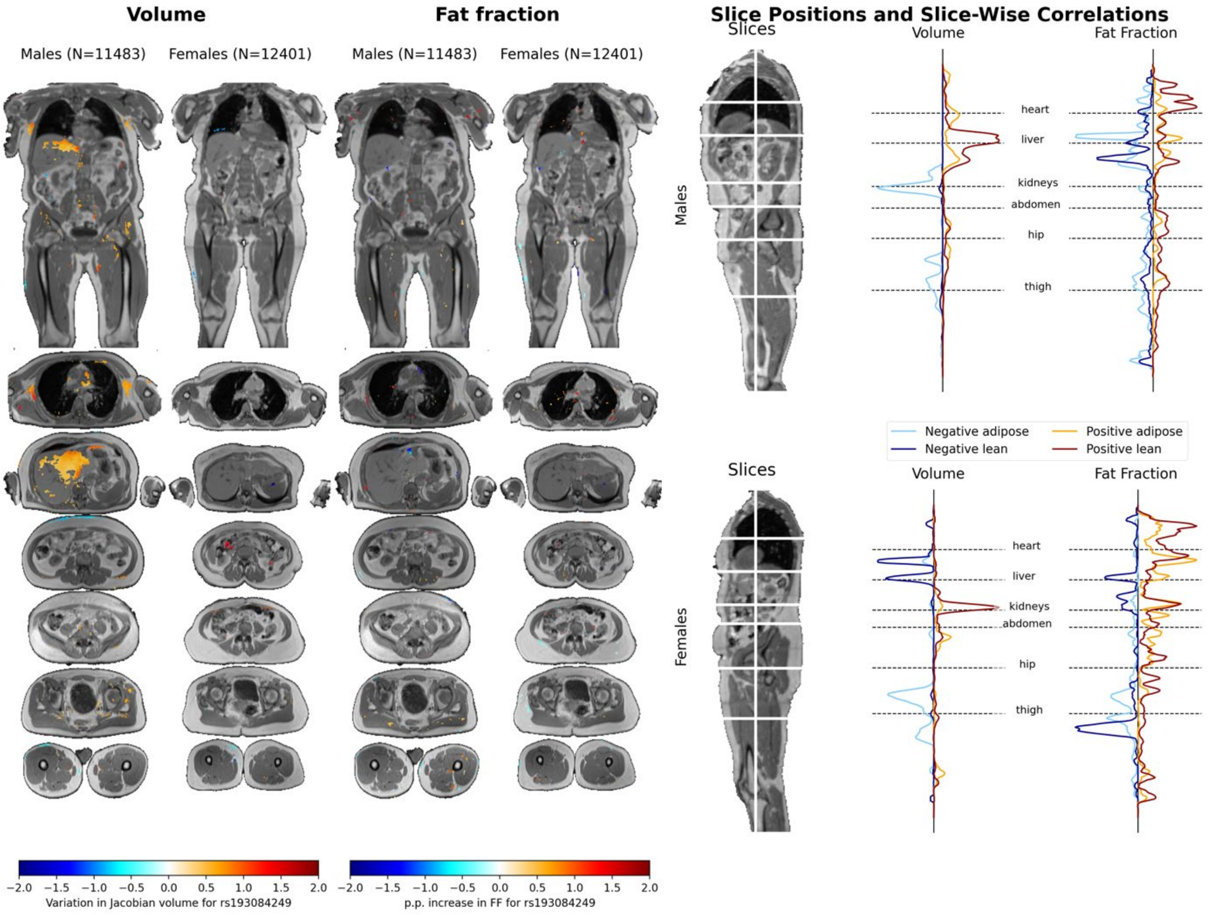

Fig. S11. Imiomics maps for the *ARID1A* rs193084249 variant that quantifies its association to tissue volume and fat fraction voxel-wise throughout the neck-to-knee region. The resulting images show, from left to right, the associations to volume for males, the associations to volume for females, the associations to fat fractions for males, and the associations to fat fractions for females. Color mapping is used to visualize the quantified associations where significant (p-value $<2.8\text{x}10^{-3}$). For visualization purposes clipped beta values (from the lower 1% to the highest 1%) were for each experiment linearly rescaled between -2 to +2 maintaining the sign of the association. Non-significant regions are not colored; but instead, a mix of the water and fat magnetic resonance images for the chosen male and female template subjects are shown to allow orientation in the body regions. The first row of images represents coronal slices of the maps, while the remaining rows represent axial slices of those maps in levels of locations of interest (heart, liver, kidneys, abdomen, hip, and thighs). The mid-sagittal slices on the right-hand side of the plots illustrate the visualized slices. The plots on the right-hand side of the collage figures represent the relative amount, from neck to knee, of significant positive and negative associations in the 3D data. The associations are reported separately from lean tissues (where the water signal is stronger than the fat signal) and from adipose tissue (where the fat signal is stronger than the water signal). Full 3D associations are visualized in **Movie S5** (axial and coronal planes).

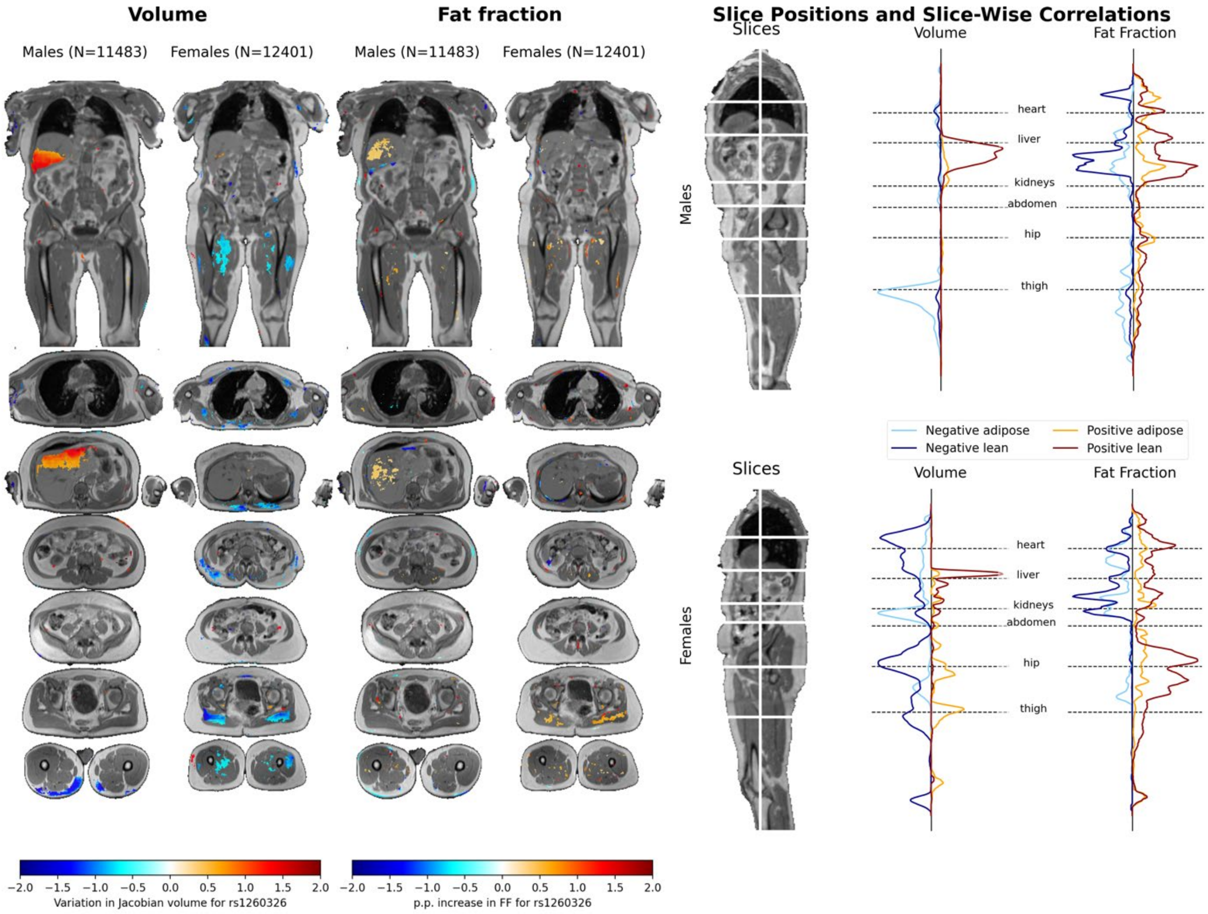

Fig. S12. Imiomics maps for the *GCKR* rs1260326 variant that quantifies its association to tissue volume and fat fraction voxel-wise throughout the neck-to-knee region. The resulting images show, from left to right, the associations to volume for males, the associations to volume for females, the associations to fat fractions for males, and the associations to fat fractions for females. Color mapping is used to visualize the quantified associations where significant (*p*-value <2.8×10^{-3} For visualization purposes clipped beta values (from the lower 1% to the highest 1%) were for each experiment linearly rescaled between -2 to +2 maintaining the sign of the association. Non-significant regions are not colored; but instead, a mix of the water and fat magnetic resonance images for the chosen male and female template subjects are shown to allow orientation in the body regions. The first row of images represents coronal slices of the maps, while the remaining rows represent axial slices of those maps in levels of locations of interest (heart, liver, kidneys, abdomen, hip, and thighs). The mid-sagittal slices on the right-hand side of the plots illustrate the visualized slices. The plots on the right-hand side of the collage figures represent the relative amount, from neck to knee, of significant positive and negative associations in the 3D data. The associations are reported separately from lean tissues (where the water signal is stronger than the fat signal) and from adipose tissue (where the fat signal is stronger than the water signal). Full 3D associations are visualized in **Movie S6** (axial and coronal planes).

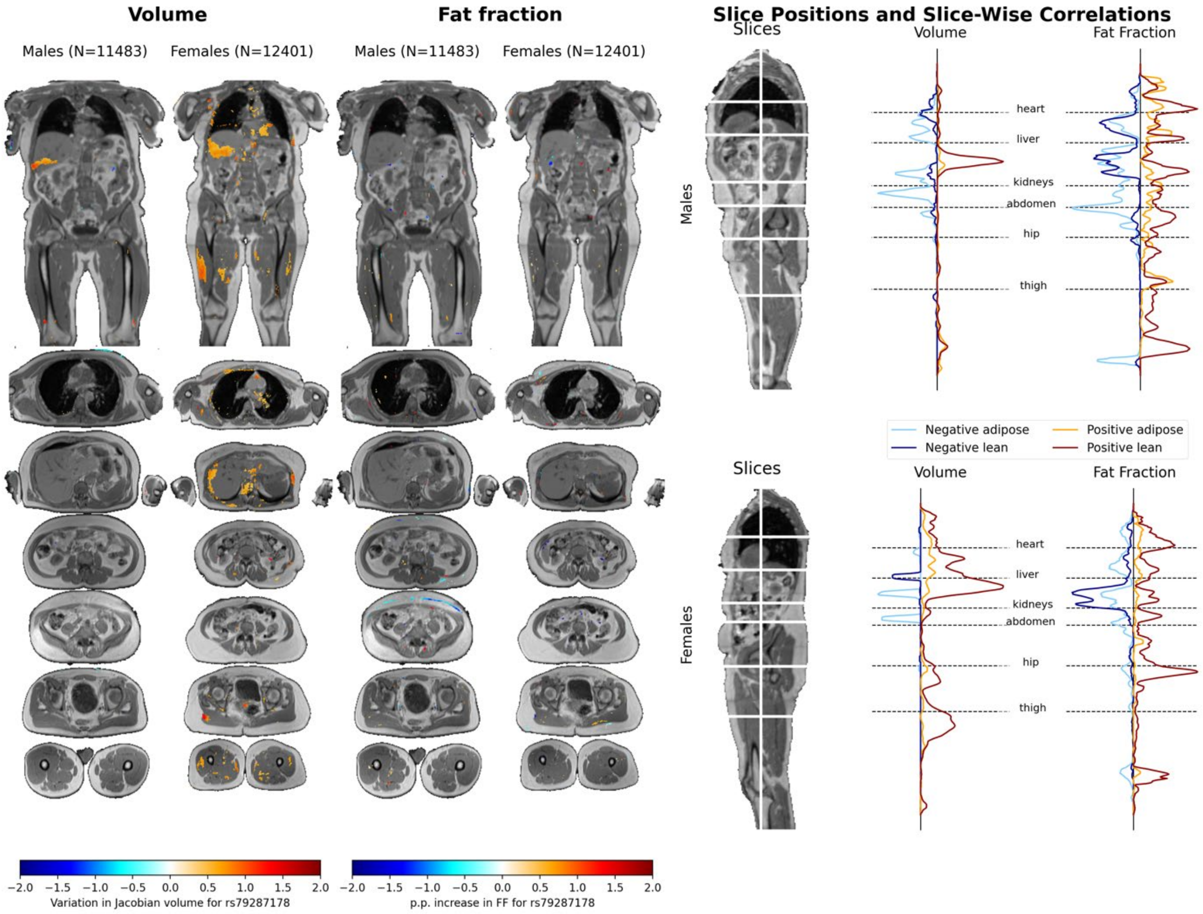

Fig. S13. Imiomics maps for the *TNFSF10* rs79287178 variant that quantifies its association to tissue volume and fat fraction voxel-wise throughout the neck-to-knee region. The resulting images show, from left to right, the associations to volume for males, the associations to volume for females, the associations to fat fractions for males, and the associations to fat fractions for females. Color mapping is used to visualize the quantified associations where significant (*p*-value $<2.8\text{x}10^{-3}$). For visualization purposes clipped beta values (from the lower 1% to the highest 1%) were for each experiment linearly rescaled between -2 to +2 maintaining the sign of the association. Non-significant regions are not colored; but instead, a mix of the water and fat magnetic resonance images for the chosen male and female template subjects are shown to allow orientation in the body regions. The first row of images represents coronal slices of the maps, while the remaining rows represent axial slices of those maps in levels of locations of interest (heart, liver, kidneys, abdomen, hip, and thighs). The mid-sagittal slices on the right-hand side of the plots illustrate the visualized slices. The plots on the right-hand side of the collage figures represent the relative amount, from neck to knee, of significant positive and negative associations in the 3D data. The associations are reported separately from lean tissues (where the water signal is stronger than the fat signal) and from adipose tissue (where the fat signal is stronger than the water signal). Full 3D associations are visualized in **Movie S7** (axial and coronal planes).

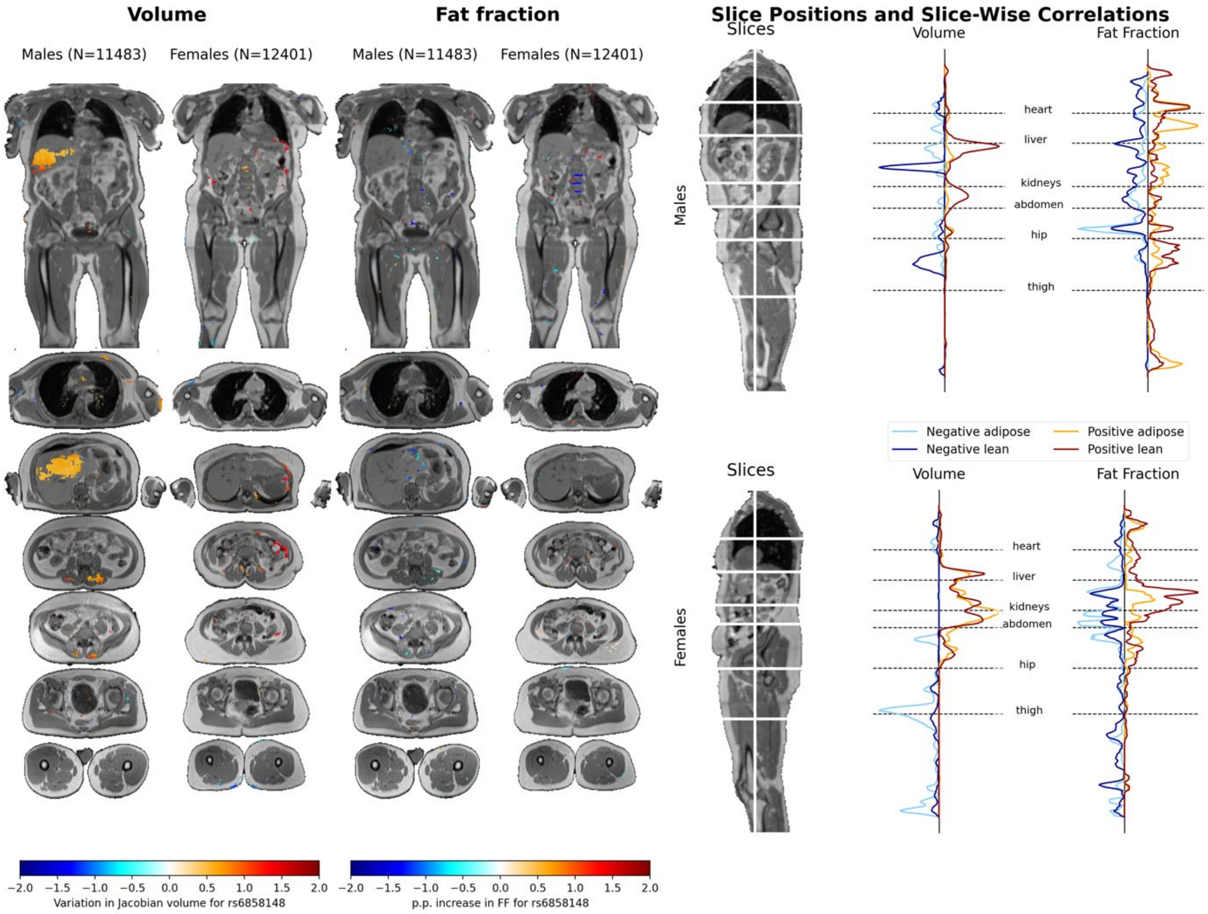

Fig. S14. Imiomics maps for the *ADH4* rs6858148 variant that quantifies its association to tissue volume and fat fraction voxel-wise throughout the neck-to-knee region. The resulting images show, from left to right, the associations to volume for males, the associations to volume for females, the associations to fat fractions for males, and the associations to fat fractions for females. Color mapping is used to visualize the quantified associations where significant (*p*-value $<2.8\text{x}10^{-3}$). For visualization purposes clipped beta values (from the lower 1% to the highest 1%) were for each experiment linearly rescaled between -2 to +2 maintaining the sign of the association. Non-significant regions are not colored; but instead, a mix of the water and fat magnetic resonance images for the chosen male and female template subjects are shown to allow orientation in the body regions. The first row of images represents coronal slices of the maps, while the remaining rows represent axial slices of those maps in levels of locations of interest (heart, liver, kidneys, abdomen, hip, and thighs). The mid-sagittal slices on the right-hand side of the plots illustrate the visualized slices. The plots on the right-hand side of the collage figures represent the relative amount, from neck to knee, of significant positive and negative associations in the 3D data. The associations are reported separately from lean tissues (where the water signal is stronger than the fat signal) and from adipose tissue (where the fat signal is stronger than the water signal). Full 3D associations are visualized in **Movie S8** (axial and coronal planes).

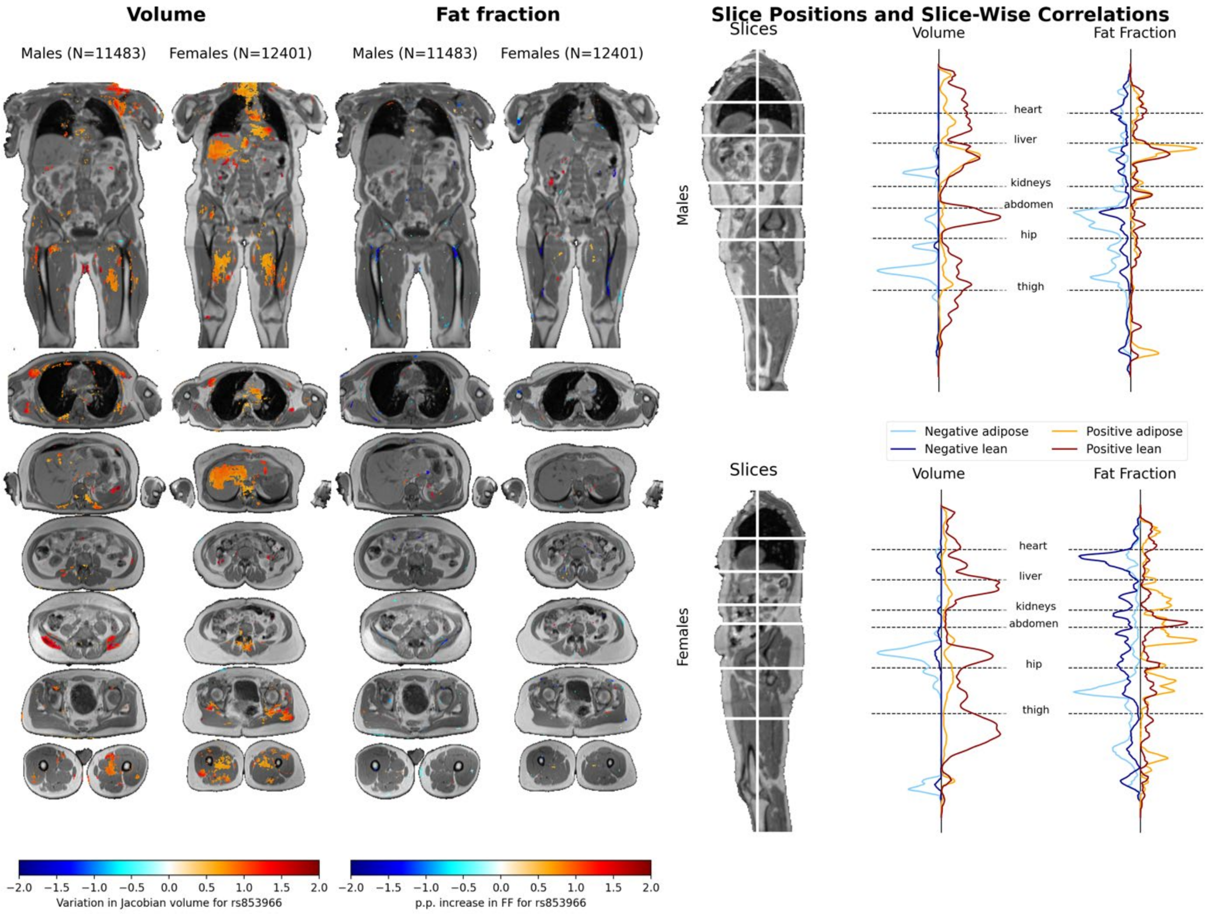

Fig. S15. Imiomics maps for the *CENPW* rs853966 variant that quantifies its association to tissue volume and fat fraction voxel-wise throughout the neck-to-knee region. The resulting images show, from left to right, the associations to volume for males, the associations to volume for females, the associations to fat fractions for males, and the associations to fat fractions for females. Color mapping is used to visualize the quantified associations where significant (p-value <$2.8x10^{-3}$). For visualization purposes clipped beta values (from the lower 1% to the highest 1%) were for each experiment linearly rescaled between -2 to +2 maintaining the sign of the association. Non-significant regions are not colored; but instead, a mix of the water and fat magnetic resonance images for the chosen male and female template subjects are shown to allow orientation in the body regions. The first row of images represents coronal slices of the maps, while the remaining rows represent axial slices of those maps in levels of locations of interest (heart, liver, kidneys, abdomen, hip, and thighs). The mid-sagittal slices on the right-hand side of the plots illustrate the visualized slices. The plots on the right-hand side of the collage figures represent the relative amount, from neck to knee, of significant positive and negative associations in the 3D data. The associations are reported separately from lean tissues (where the water signal is stronger than the fat signal) and from adipose tissue (where the fat signal is stronger than the water signal). Full 3D associations are visualized in **Movie S9** (axial and coronal planes).

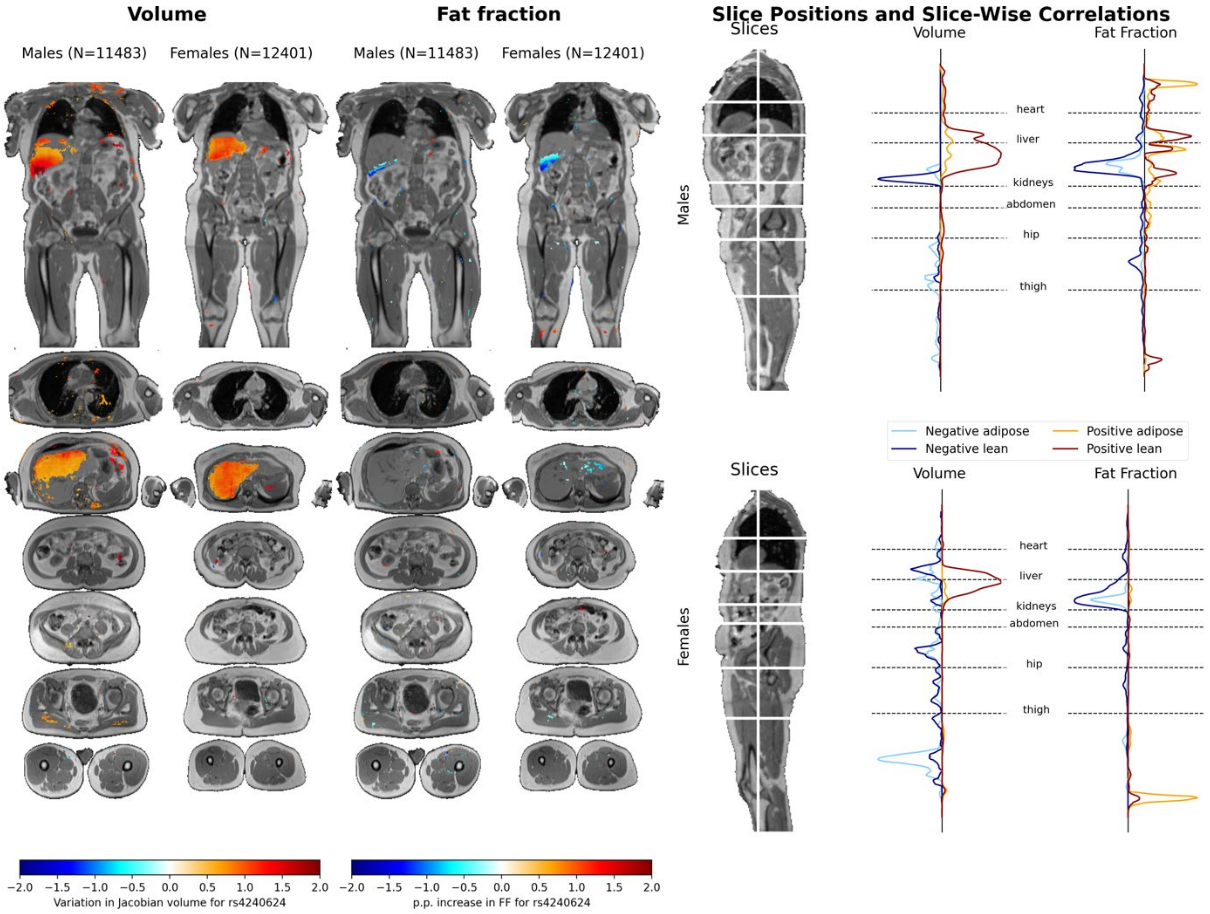

Fig. S16. Imiomics maps for the *PPP1R3B* rs4240624 variant that quantifies its association to tissue volume and fat fraction voxel-wise throughout the neck-to-knee region. The resulting images show, from left to right, the associations to volume for males, the associations to volume for females, the associations to fat fractions for males, and the associations to fat fractions for females. Color mapping is used to visualize the quantified associations where significant (*p*-value <2.8×10^{-3}). For visualization purposes clipped beta values (from the lower 1% to the highest 1%) were for each experiment linearly rescaled between -2 to +2 maintaining the sign of the association. Non-significant regions are not colored; but instead, a mix of the water and fat magnetic resonance images for the chosen male and female template subjects are shown to allow orientation in the body regions. The first row of images represents coronal slices of the maps, while the remaining rows represent axial slices of those maps in levels of locations of interest (heart, liver, kidneys, abdomen, hip, and thighs). The mid-sagittal slices on the right-hand side of the plots illustrate the visualized slices. The plots on the right-hand side of the collage figures represent the relative amount, from neck to knee, of significant positive and negative associations in the 3D data. The associations are reported separately from lean tissues (where the water signal is stronger than the fat signal) and from adipose tissue (where the fat signal is stronger than the water signal). Full 3D associations are visualized in **Movie S10** (axial and coronal planes).

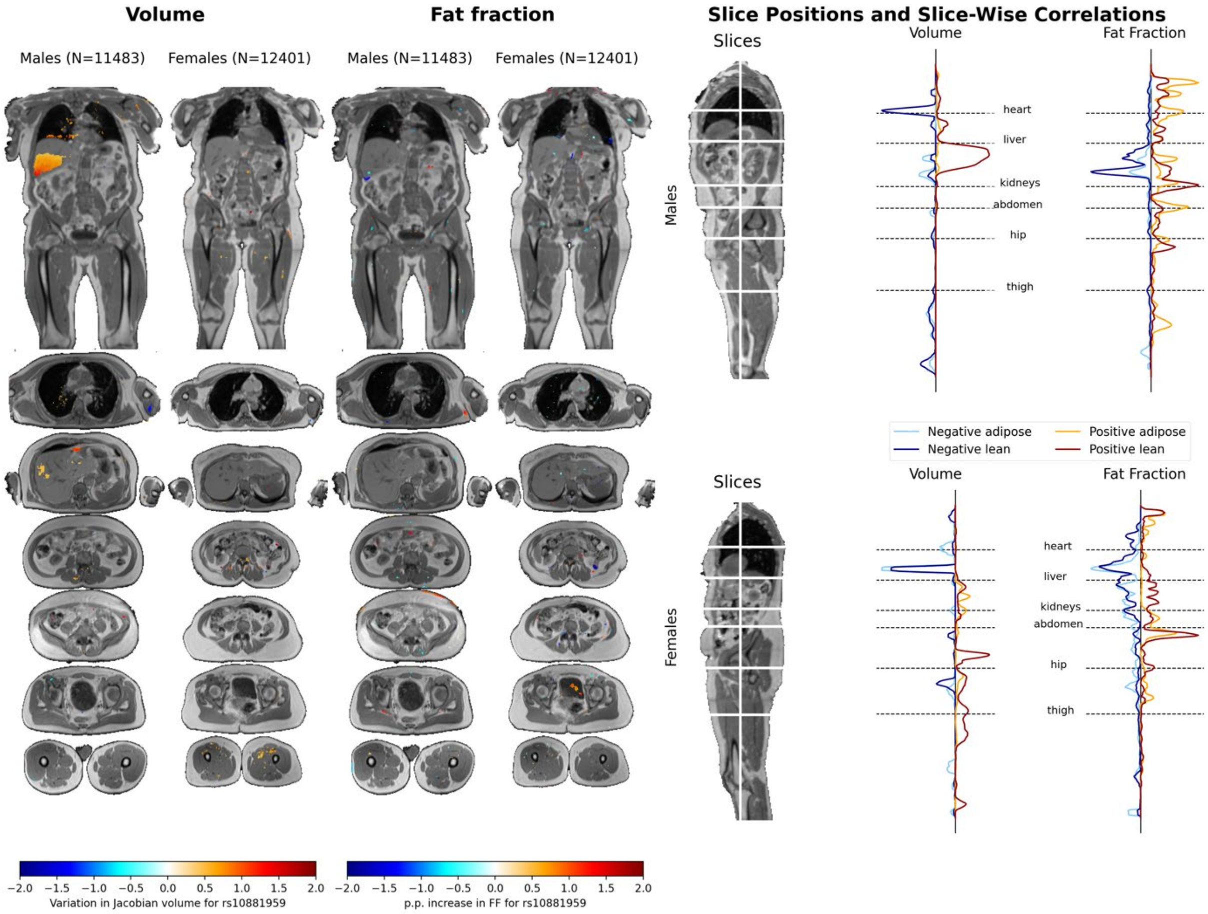

Fig. S17. Imiomics maps for the *TNKS2* rs10881959 variant that quantifies its association to tissue volume and fat fraction voxel-wise throughout the neck-to-knee region. The resulting images show, from left to right, the associations to volume for males, the associations to volume for females, the associations to fat fractions for males, and the associations to fat fractions for females. Color mapping is used to visualize the quantified associations where significant (p-value $<2.8\text{x}10^{-3}$). For visualization purposes clipped beta values (from the lower 1% to the highest 1%) were for each experiment linearly rescaled between -2 to +2 maintaining the sign of the association. Non-significant regions are not colored; but instead, a mix of the water and fat magnetic resonance images for the chosen male and female template subjects are shown to allow orientation in the body regions. The first row of images represents coronal slices of the maps, while the remaining rows represent axial slices of those maps in levels of locations of interest (heart, liver, kidneys, abdomen, hip, and thighs). The mid-sagittal slices on the right-hand side of the plots illustrate the visualized slices. The plots on the right-hand side of the collage figures represent the relative amount, from neck to knee, of significant positive and negative associations in the 3D data. The associations are reported separately from lean tissues (where the water signal is stronger than the fat signal) and from adipose tissue (where the fat signal is stronger than the water signal). Full 3D associations are visualized in **Movie S11** (axial and coronal planes).

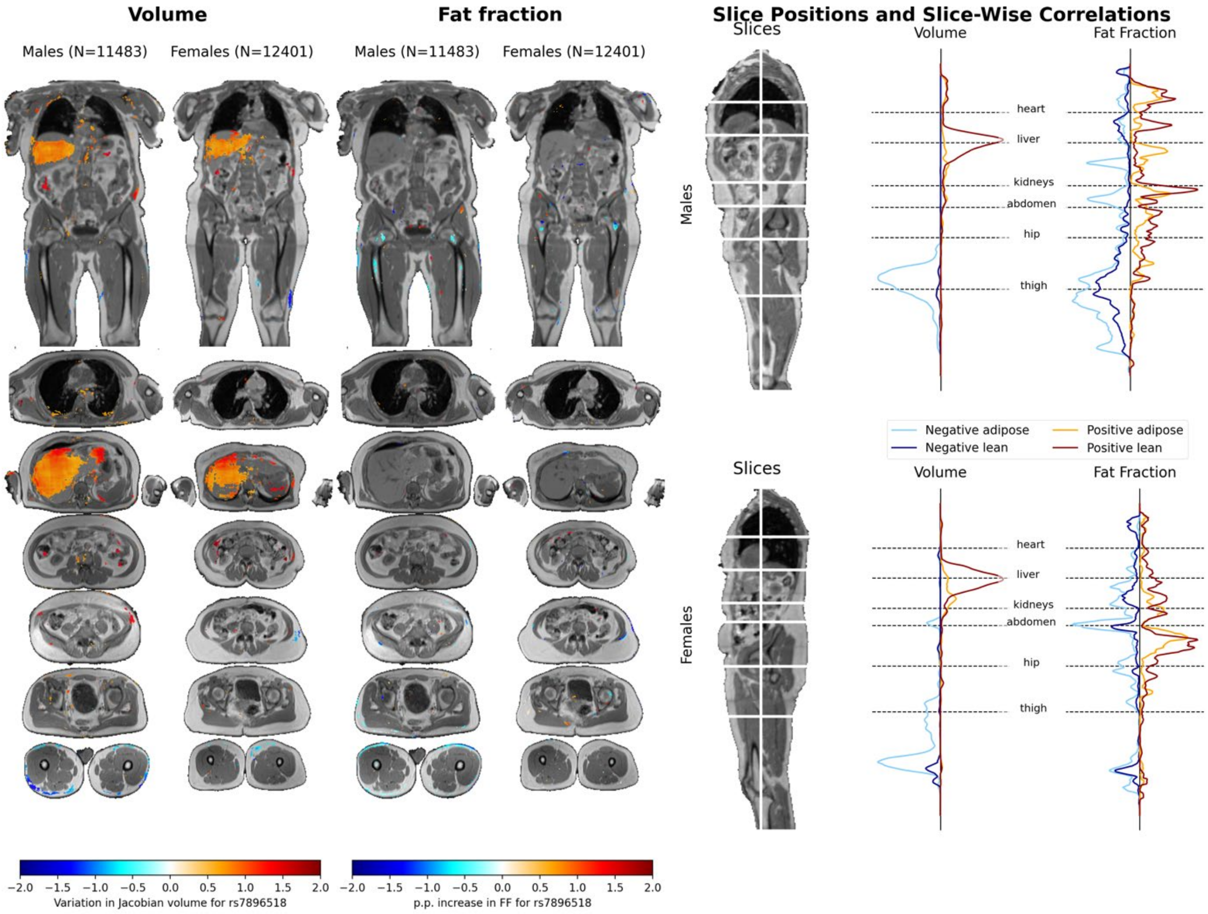

Fig. S18. Imiomics maps for the *REEP3* rs7896518 variant that quantifies its association to tissue volume and fat fraction voxel-wise throughout the neck-to-knee region. The resulting images show, from left to right, the associations to volume for males, the associations to volume for females, the associations to fat fractions for males, and the associations to fat fractions for females. Color mapping is used to visualize the quantified associations where significant (p-value <2.8×10^{-3}). For visualization purposes clipped beta values (from the lower 1% to the highest 1%) were for each experiment linearly rescaled between -2 to +2 maintaining the sign of the association. Non-significant regions are not colored; but instead, a mix of the water and fat magnetic resonance images for the chosen male and female template subjects are shown to allow orientation in the body regions. The first row of images represents coronal slices of the maps, while the remaining rows represent axial slices of those maps in levels of locations of interest (heart, liver, kidneys, abdomen, hip, and thighs). The mid-sagittal slices on the right-hand side of the plots illustrate the visualized slices. The plots on the right-hand side of the collage figures represent the relative amount, from neck to knee, of significant positive and negative associations in the 3D data. The associations are reported separately from lean tissues (where the water signal is stronger than the fat signal) and from adipose tissue (where the fat signal is stronger than the water signal). Full 3D associations are visualized in **Movie S12** (axial and coronal planes).

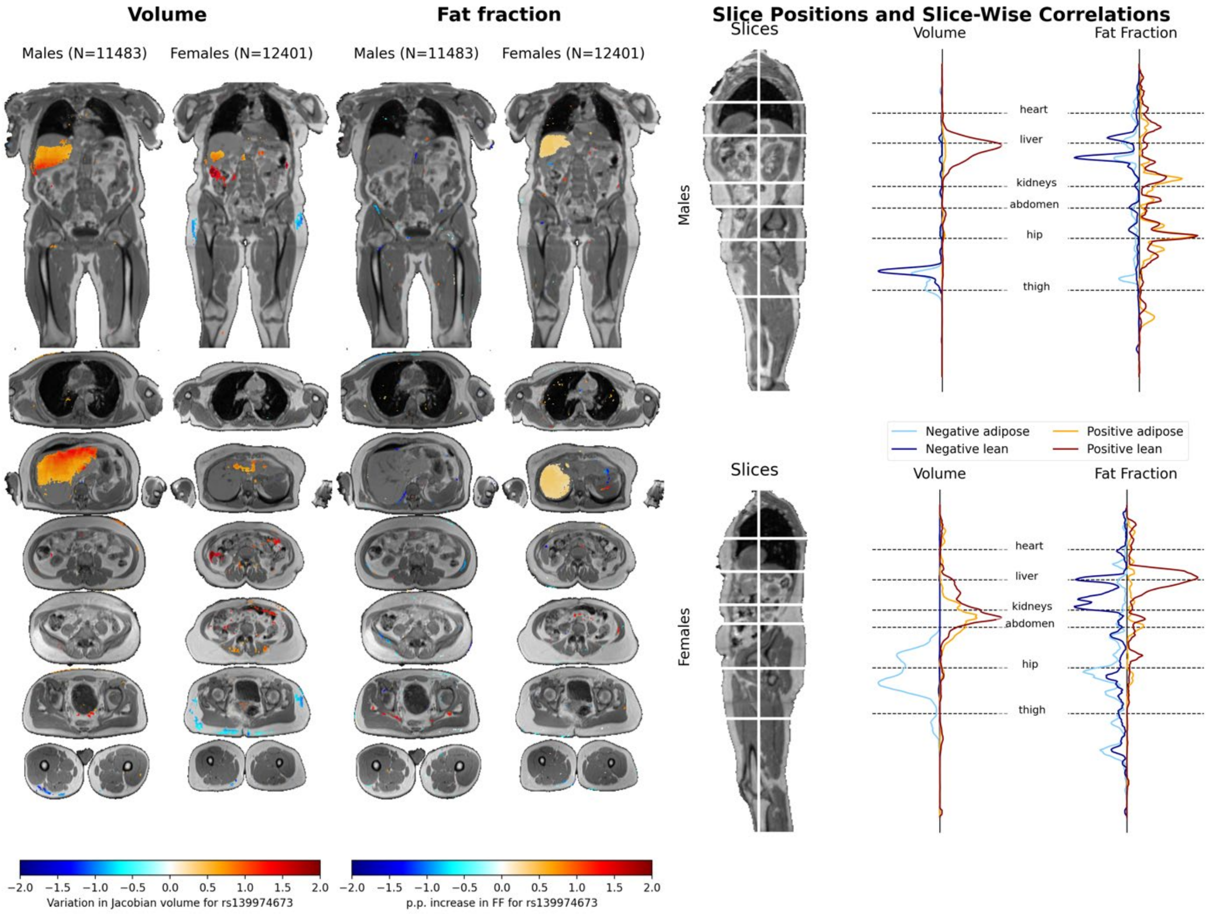

Fig. S19. Imiomics maps for the *PDIA3* rs139974673 variant that quantifies its association to tissue volume and fat fraction voxel-wise throughout the neck-to-knee region. The resulting images show, from left to right, the associations to volume for males, the associations to volume for females, the associations to fat fractions for males, and the associations to fat fractions for females. Color mapping is used to visualize the quantified associations where significant (p-value $<2.8\times10^{-3}$). For visualization purposes clipped beta values (from the lower 1% to the highest 1%) were for each experiment linearly rescaled between -2 to +2 maintaining the sign of the association. Non-significant regions are not colored; but instead, a mix of the water and fat magnetic resonance images for the chosen male and female template subjects are shown to allow orientation in the body regions. The first row of images represents coronal slices of the maps, while the remaining rows represent axial slices of those maps in levels of locations of interest (heart, liver, kidneys, abdomen, hip, and thighs). The mid-sagittal slices on the right-hand side of the plots illustrate the visualized slices. The plots on the right-hand side of the collage figures represent the relative amount, from neck to knee, of significant positive and negative associations in the 3D data. The associations are reported separately from lean tissues (where the water signal is stronger than the fat signal) and from adipose tissue (where the fat signal is stronger than the water signal). Full 3D associations are visualized in **Movie S13** (axial and coronal planes).

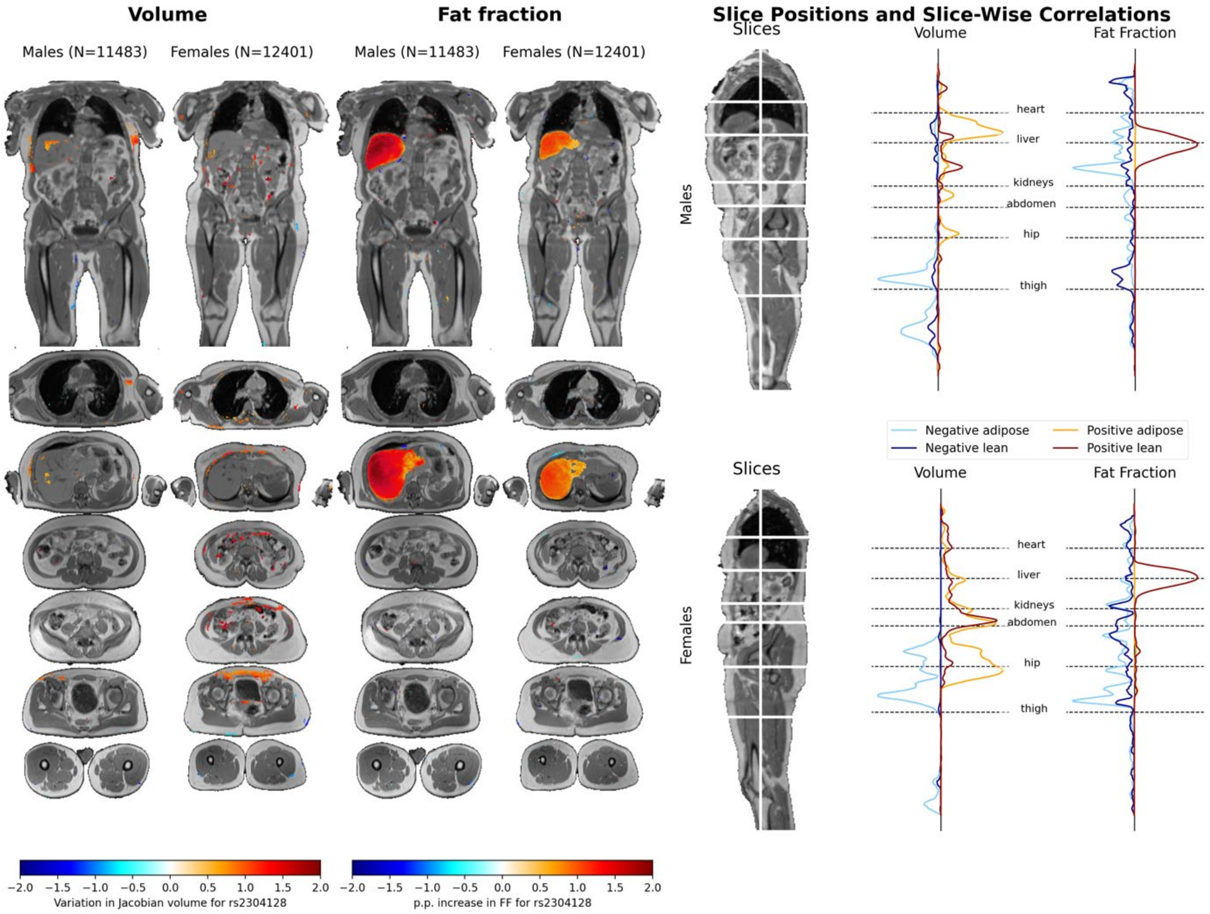

Fig. S20. Imiomics maps for the *LPAR2* rs2304128 variant that quantifies its association to tissue volume and fat fraction voxel-wise throughout the neck-to-knee region. The resulting images show, from left to right, the associations to volume for males, the associations to volume for females, the associations to fat fractions for males, and the associations to fat fractions for females. Color mapping is used to visualize the quantified associations where significant (p-value $<2.8x10^{-3}$). For visualization purposes clipped beta values (from the lower 1% to the highest 1%) were for each experiment linearly rescaled between -2 to +2 maintaining the sign of the association. Non-significant regions are not colored; but instead, a mix of the water and fat magnetic resonance images for the chosen male and female template subjects are shown to allow orientation in the body regions. The first row of images represents coronal slices of the maps, while the remaining rows represent axial slices of those maps in levels of locations of interest (heart, liver, kidneys, abdomen, hip, and thighs). The mid-sagittal slices on the right-hand side of the plots illustrate the visualized slices. The plots on the right-hand side of the collage figures represent the relative amount, from neck to knee, of significant positive and negative associations in the 3D data. The associations are reported separately from lean tissues (where the water signal is stronger than the fat signal) and from adipose tissue (where the fat signal is stronger than the water signal). Full 3D associations are visualized in **Movie S14** (axial and coronal planes).

Supplementary tables (provided in separate excel file)

Table S1. Variables used in the UK Biobank with their respective data-fields.

Table S2. Fine mapping and gene prioritization of liver fat and liver volume genetics variants.

Table S3. Cross-trait associations of liver fat and liver volume genetic variants and their replication in a previous UK Biobank GWAS.

Table S4. Liver fat- and liver volume-associated genetic variants in relation to visceral adipose tissue (VAT) and abdominal subcutaneous adipose tissue (SAT).

Table S5. Genome-wide association analysis of liver volume unadjusted for height (n=24,752).

Supplementary movies (provided in separate files)

Movies S1-S4: 3D visualization of associations in the axial plane and coronal plane for each liver fat genetic variant.

Movies S5-S14: 3D visualization of associations in the axial plane and coronal plane for each liver volume genetic variant.

Supplementary references

[1] Fry D, Almond R, Moffat S, et al. UK Biobank Biomarker Project Companion Document to Accompany Serum Biomarker Data. http://biobankndphoxacuk/showcase/showcase/docs/serum_biochemistrypdf 2019.

[2] Peila R, Rohan TE. Diabetes, Glycated Hemoglobin, and Risk of Cancer in the UK Biobank Study. Cancer Epidemiol Biomarkers Prev 2020.

[3] Censin JC, Peters SAE, Bovijn J, et al. Causal relationships between obesity and the leading causes of death in women and men. PLoS Genet 2019;15:e1008405.

[4] Strand R, Malmberg F, Johansson L, et al. A concept for holistic whole body MRI data analysis, Imiomics. PLoS One 2017;12:e0169966.

[5] Ekstrom S, Malmberg F, Ahlstrom H, et al. Fast graph-cut based optimization for practical dense deformable registration of volume images. Comput Med Imaging Graph 2020;84:101745.

[6] Clarke TK, Adams MJ, Davies G, et al. Genome-wide association study of alcohol consumption and genetic overlap with other health-related traits in UK Biobank (N=112 117). Mol Psychiatry 2017;22:1376-1384.

[7] Howe LJ, Lawson DJ, Davies NM, et al. Genetic evidence for assortative mating on alcohol consumption in the UK Biobank. Nat Commun 2019;10:5039.

[8] Kurki MI, Karjalainen J, Palta P, et al. FinnGen provides genetic insights from a well-phenotyped isolated population. Nature 2023;613:508-518.

[9] Bulik-Sullivan BK, Loh PR, Finucane HK, et al. LD Score regression distinguishes confounding from polygenicity in genome-wide association studies. Nat Genet 2015;47:291-295.

[10] Feizi A, Ray K. otargen: GraphQL-based R package for tidy data accessing and processing from Open Targets Genetics. Bioinformatics 2023;39.

[11] Gazal S, Weissbrod O, Hormozdiari F, et al. Combining SNP-to-gene linking strategies to identify disease genes and assess disease omnigenicity. Nat Genet 2022;54:827-836.

[12] Yang Z, Wang C, Liu L, et al. CARMA is a new Bayesian model for fine-mapping in genome-wide association meta-analyses. Nat Genet 2023;55:1057-1065.
